# G3BP1 inhibits Cul3^SPOP^ to amplify AR signaling and promote prostate cancer

**DOI:** 10.1038/s41467-021-27024-x

**Published:** 2021-11-18

**Authors:** Chandrani Mukhopadhyay, Chenyi Yang, Limei Xu, Deli Liu, Yu Wang, Dennis Huang, Lesa Dayal Deonarine, Joanna Cyrta, Elai Davicioni, Andrea Sboner, Brian. D. Robinson, Arul M. Chinnaiyan, Mark A. Rubin, Christopher E. Barbieri, Pengbo Zhou

**Affiliations:** 1 Department of Pathology and Laboratory Medicine, Weill Medical College of Cornell University, New York, NY 10065 USA; 2 Sandra and Edward Meyer Cancer Center, Weill Cornell Medicine, New York, NY 10065 USA; 3 Department of Urology, Weill Cornell Medicine, New York, NY 10065 USA; 4 HRH Prince Alwaleed Bin Talal Bin Abdulaziz Alsaud Institute for Computational Biomedicine, Weill Cornell Medical College, New York, NY 10065 USA; 5 GenomeDx Bioscience, Vancouver, BC Canada; 6 Englander Institute for Precision Medicine of Weill Cornell Medicine and New York-Presbyterian Hospital, New York, NY 10065 USA; 7 Michigan Center for Translational Pathology, University of Michigan, Ann Arbor, MI USA; 8 Department for Biomedical Research, University of Bern, 3008 Bern, Switzerland

**Keywords:** Prostate cancer, Ubiquitin ligases

## Abstract

SPOP, an E3 ubiquitin ligase, acts as a prostate-specific tumor suppressor with several key substrates mediating oncogenic function. However, the mechanisms underlying SPOP regulation are largely unknown. Here, we have identified G3BP1 as an interactor of SPOP and functions as a competitive inhibitor of Cul3^SPOP^, suggesting a distinctive mode of Cul3^SPOP^ inactivation in prostate cancer (PCa). Transcriptomic analysis and functional studies reveal a G3BP1-SPOP ubiquitin signaling axis that promotes PCa progression through activating AR signaling. Moreover, AR directly upregulates G3BP1 transcription to further amplify G3BP1-SPOP signaling in a feed-forward manner. Our study supports a fundamental role of G3BP1 in disabling the tumor suppressive Cul3^SPOP^, thus defining a PCa cohort independent of SPOP mutation. Therefore, there are significantly more PCa that are defective for SPOP ubiquitin ligase than previously appreciated, and these G3BP1^high^ PCa are more susceptible to AR-targeted therapy.

## Abstract (web summary)

SPOP functions as a tumour suppressor in prostate cancer but how the protein is regulated is unclear. Here, the authors identify G3BP1 as a competitive inhibitor of SPOP and show that G3BP1-SPOP axis activates androgen signalling to drive tumorigenesis.

## Introduction

The androgen receptor (AR) is a critical driver of PCa pathophysiology, regulating proliferation, migration and metabolism; it is also a validated therapeutic target^1,2^. Importantly, Speckle-type POZ protein (SPOP) acts as a tumor suppressor through its function as a substrate receptor of the Cullin 3 (CUL3)-based ubiquitin ligase^3–6^ and directs ubiquitin-proteasomal degradation of key regulators, (e.g., AR, SRC3, TRIM24, and DEK)^7–10^. As such, SPOP plays an unique role in maintaining a steady-state level of AR signaling in prostate epithelial cells^7,8^ and regulating oncogenic transcription, DNA damage repair, and tumor cell migration^11–13^. SPOP mutations occur 10–15% of Pca (prostate adenocarcinoma)^11,14–16^, which have been found to be present predominantly in the substrate-binding MATH domain; such mutations inactivate SPOP’s function by disrupting SPOP-substrate interactions, thereby altering the steady-state levels of key components in the AR signaling pathway and contributing to PCa development^8,17^. SPOP mutations have also been found outside of the MATH domain, but functional significance has yet to be demonstrated.

Although multiple downstream effectors of SPOP have been defined, little is known about upstream regulatory mechanisms that may modulate the tumor suppressive function of SPOP. Here, we show that GTPase Activating Protein (SH3 Domain) Binding Protein 1 (G3BP1) interacts with the CUL3^SPOP^ complex and competes with SPOP substrates for binding to the CUL3^SPOP^ ubiquitin ligase. As a cytosolic protein, G3BP1 plays role in protecting mRNAs through the formation of stress granules; such protection is thought to be involved in prostate tumorigenesis and response to therapy^18–22^. G3BP1 also acting as a DNA helicase in the nucleus^23,24^; however, function of G3BP1 in nucleus is less clear. G3BP1 is an established oncogene in breast, head and neck, colon, thyroid, and pancreatic cancer^25,26^. G3BP1 is potentially capable of enhanced tumor formation with highly proliferative phenotypes in other cancers^24,27–29^. Recent studies suggest that G3BP1 acts both in stress-dependent and in stress-independent manner as switch for the formation of different macromolecular complexes^30–32^. Our studies reveal a unique oncogenic role of stress responsive protein G3BP1 that negatively regulates tumor-suppressive SPOP ubiquitin ligase, leading to upregulation of AR signaling and prostate tumorigenesis. The study reveals an upstream regulatory pathway of SPOP inactivation and sheds light on another intervention strategy against prostate cancer.

## Results

### G3BP1 is a competitive inhibitor of SPOP ubiquitin ligase

**Fig. 1:**
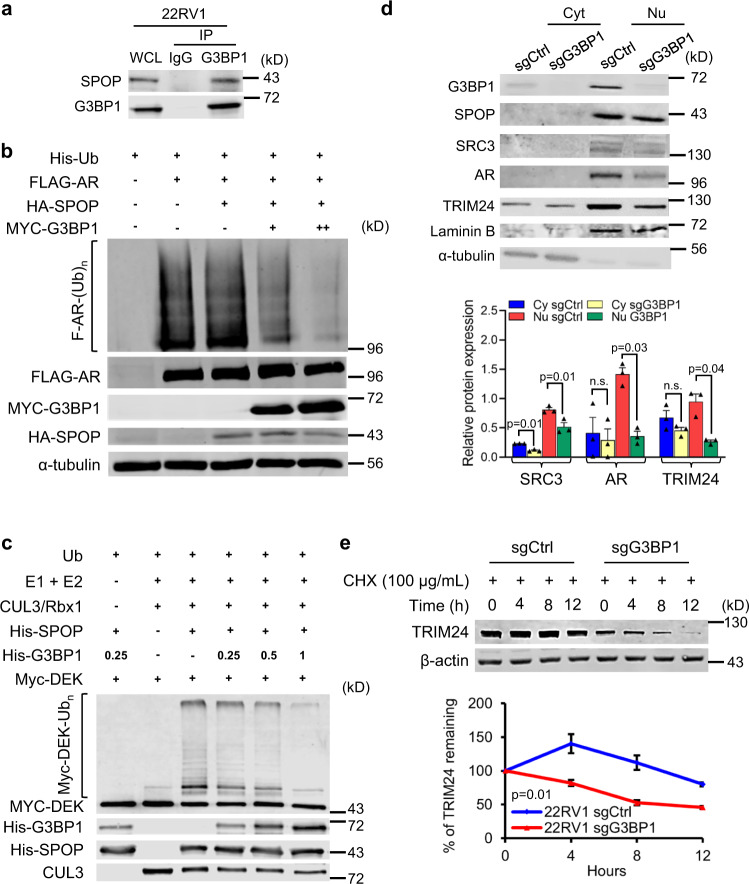
G3BP1 interacts with SPOP and acts as a negative regulator of SPOP ubiquitin ligase. **a** 22Rv1 cell lysates were subjected to immunoprecipitation (IP) with IgG and anti-G3BP1 antibody and immunoblotted with anti-SPOP and anti-G3BP1 antibodies. *n* = 3. **b** HEK 293T cells were transiently transfected with His-Ub and the indicated construct(s) for 48 h. Cell lysates were subjected to His-Ub pulldown using nickel-NTA beads under denaturing conditions, followed by SDS–PAGE, and immunoblotting with the anti-FLAG antibody. Expression of FLAG-AR, MYC-G3BP1, and HA-SPOP was detected by immunoblotting. α-tubulin served as an internal loading control. *n* = 3. **c** DEK ubiquitination (Ub) was reconstituted in vitro using affinity-purified recombinant proteins as indicated. Purified proteins and ubiquitinated DEK were subjected to immunoblotting with the indicated antibodies. All protein concentrations were in nM except Ub, which was in µM. *n* = 3. **d** Immunoblotting of cytosolic and nuclear fraction of LNCaP-sgCtrl and LNCaP-sgG3BP1 cells with the indicated antibodies. Results from three independent experiments were quantitated by densitometry and relative protein expression of SRC3, AR, and TRIM24 in LNCaP-sgG3BP1 cells relative to those of LNCaP-sgCtrl were plotted. Error bars, ±S.E.M. Paired *t*-test. **e** Cycloheximide (CHX)-chase analysis to determine the half-life of endogenous TRIM24 in 22Rv1-sgCtrl and 22Rv1-sgG3BP1 cells. The percentage of TRIM24 remaining was graphed at the time points indicated. *n* = 3 biologically independent experiments. Error bars, ±S.E.M. Paired *t*-test. *p* value is indicated in figure. Source data are provided as a Source Data file. WCL whole cell lysate, Cyt cytoplasmic extract, Nu nuclear extract.

To gain a comprehensive understanding of the SPOP signaling network, we conducted tandem affinity purification combined with mass spectrometry (TAP-MS) to identify SPOP interactor(s) (Supplementary Fig. 1A). We used N-terminal substrate-binding MATH domain of SPOP (SPOP^MATH^) that cannot assemble with CUL3-Rbx1, thereby preventing subsequent ubiquitin-proteasomal degradation and enriching the SPOP-substrate interactions. The PCa-associated mutant SPOP^MATH(F102C)^, which is defective for substrate binding, was used as a control. G3BP1 was found to bind to SPOP^MATH^ but not SPOP^MATH(F102C)^ in 22Rv1 (AR^+^) PCa cells (see below). Association of endogenous G3BP1 and SPOP was validated through co-immunoprecipitation (Fig. 1a and Supplementary Fig. 1B) and proximity ligation assay (Supplementary Fig. 1C). In this regard, Theurillat and colleagues identified G3BP1 in an SPOP-based ubiquitylome analysis^10^.

As a cytosolic protein, G3BP1 is involved in the formation of stress granules when cells are under stress conditions^25^, but it also translocate to the nucleus^33^, where its function is less clear. Using confocal immunofluorescence, we found that G3BP1 co-localizes with SPOP in the nucleus (Supplementary Fig. 1D). Next, we sought to understand the biochemical consequences of G3BP1 and SPOP interaction. Silencing of SPOP led to upregulation of its substrate TRIM24 (Supplementary Fig. 1E)^9^, yet had no effect on G3BP1 levels (Supplementary Fig. 1E). Upon MG132 treatment there is an increased accumulation of SPOP substrates TRIM24 and SRC3 but not G3BP1 in 22Rv1 cells (Supplementary Fig. 1H). This suggests that unlike conventional SPOP substrates G3BP1 is not subjected to SPOP mediated proteasomal degradation.

Next, we investigated the role of G3BP1 in modulating SPOP’s ubiquitin ligase function. Increased expression of G3BP1 resulted in reduced ubiquitination of SPOP substrates such as AR in HEK 293T cells (Fig. 1b). Consistent with this result, loss of G3BP1 in 22Rv1 cells upon CRISPR-mediated deletion led to a concomitant reduction of steady-state levels of SPOP substrates, suggesting that G3BP1 suppresses SPOP’s ubiquitin ligase function. To test this hypothesis, we generated LNCaP-sgG3BP1 and 22Rv1-sgG3BP1 cell lines in which the endogenous G3BP1 was deleted by CRISPR-Cas9 (Supplementary Fig. 1C, inset; Supplementary Fig. 1L). Deletion of G3BP1 restored CUL3^SPOP^ E3 ubiquitin ligase activity, as reflected by decreased levels of the CUL3^SPOP^ substrates TRIM24, AR, and SRC3 in the nuclear fraction of LNCaP cells (Fig. 1d) and accelerated turnover rates of TRIM24 protein with no change at transcript level (Fig. 1e and Supplementary Fig. 1I). Conversely, enforced expression of G3BP1 effectively elevated the levels of SPOP substrates, e.g., TRIM24, AR, and SRC3 (Supplementary Fig. 1K).

To further prove molecular mechanism underlying G3BP1-mediated suppression of CUL3^SPOP^ ubiquitin ligase complex, we reconstituted an in vitro DEK ubiquitination reaction using affinity-purified E1, UBCH5C, CUL3/Rbx1, SPOP, and DEK. As shown in Fig. 1c and Supplementary Fig. 1J, G3BP1 effectively reduced polyubiquitination of DEK in a dose-dependent manner. Notably, under this condition, DEK is robustly polyubiquitinated whereas G3BP1 is monoubiquitinated (Supplementary Fig. 1F, lane 4). Moreover, using in vitro binding assay with affinity-purified proteins, we further observed that increasing doses of G3BP1 directly competed with DEK for binding to SPOP (Supplementary Fig. 1M), suggesting that G3BP1 functions by excluding SPOP substrates resulting in reduced ubiquitination. In addition, in SPOP-null C4-2B prostate cancer cells, the turnover rate (half-life) of G3BP1 remains unchanged compared with the parental SPOP-wt C4-2B cells (Supplementary Fig. 1G). Taken together with the absence of consensus SPOP substrate-binding motif Φ−π-S/T-S/T-S/T (Φ: nonpolar residues, π: polar residues) these findings demonstrate that G3BP1 acts as a competitive inhibitor of CUL3^SPOP^ ubiquitin ligase and stabilizes substrates of SPOP, including AR and the AR co-factors (TRIM24 and SRC3), which are drivers of prostate tumorigenesis and its progression.

### Specific domains are necessary for the crosstalk between G3BP1 and SPOP

**Fig. 2:**
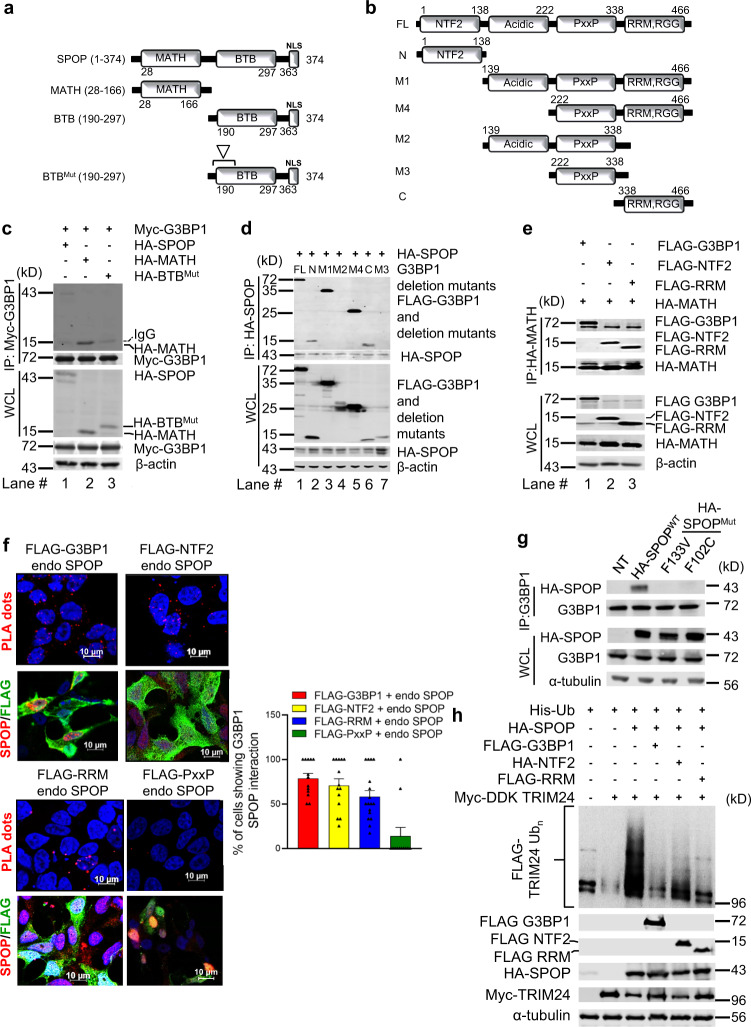
Molecular determinants of crosstalk between G3BP1 and SPOP. **a**, **b** Schematic illustration of a series of SPOP deletion mutants and G3BP1 deletion mutants used in this study. “Inverted triangle” shows SPOP dimerization sites and mutations (L186D, L190D, L193D, I217K). **c**, **d** Immunoprecipitation and immunoblotting of WCL derived from HEK 293T cells transfected with the indicated full length (FL) or (**c**) SPOP or (**d**) G3BP1 deletion constructs. β-actin served as the loading control. HA-MATH: HA-tagged MATH domain of SPOP; HA-BTB^Mut^: HA-tagged BTB domain of SPOP with mutated dimerization sites; N: NTF2 (1-138 aa), M1: G3BP1 (139-466 aa), M4: G3BP1 (222-466 aa), M2: G3BP1 (139-338 aa), M3: G3BP1 (222-338 aa), C: RRM (338-466 aa). *n* = 3. **e** Immunoprecipitation and immunoblotting of WCL derived from 22Rv1 cells transfected with the indicated construct(s). *n* = 3. **f** PLA assay for the FLAG–G3BP1 and its deletion mutants with endogenous SPOP. Each red dot represents an interaction (scale bar, 10 μm). Representative immunofluorescence images of 22Rv1 cells transfected with FLAG-G3BP1, FLAG-NTF2, FLAG-RRM, or FLAG-PxxP. A graph showing percentage of cells with G3BP1 and SPOP interaction. *n* = 3 biologically independent experiments. Error bars, ±S.E.M. **g** Parental 22Rv1 cells were transfected with either HA-SPOP^WT^ or HA-SPOP^F133V^ or HA-SPOP^F102C^. After 48 h, cell lysates were immunoprecipitated with anti-G3BP1 antibody and immunoblotted with either anti-HA-SPOP or anti-G3BP1 antibody. NT no transfection. *n* = 3. **h** HEK 293T cells were transiently transfected with the indicated construct(s) for 48 h. Ni-NTA pull-down products or WCL were blotted for the indicated proteins. α-tubulin serves as the loading control. *n* = 3. Source data are provided as a Source Data file. WCL whole cell lysate.

Both SPOP and G3BP1 proteins comprise multiple functional domains (Fig. 2a-b). To define the structural determinants for G3BP1-mediated suppression of SPOP ubiquitin ligase, we constructed a series of deletion mutants of G3BP1 and SPOP. SPOP consists of the N-terminal substrate-binding MATH domain and the C-terminal CUL3-binding BTB domain; the latter also contains the homo-dimerization module of SPOP (Fig. 2a)^34^. Immunofluorescence and confocal imaging of 22Rv1 cells transfected with either SPOP or its deletion mutants (MATH and BTB) showed predominantly nuclear localization with limited cytoplasmic distribution of MATH domain of SPOP (Supplementary Fig. 2C). Initial co-immunoprecipitation analysis showed that both SPOP^MATH^ and SPOP^BTB^ were capable of binding to G3BP1. However, the BTB domain can dimerize with endogenous SPOP, which in turn bridges the interaction of SPOP^BTB^ with proteins (e.g., G3BP1 or SPOP substrates) indirectly. Indeed, SPOP^BTB^ with mutated dimerization site(s) (L186A, L190A, L193A, and I217A) is incapable of binding to endogenous SPOP and showed no binding with G3BP1 (Fig. 2c). Therefore, G3BP1 interacts with SPOP through its substrate-binding MATH domain.

G3BP1 contains four conserved domains (Fig. 2b) known as nuclear transporter factor 2 (NTF2), proline-rich motif (PxxP), acid-rich domain (acidic), and RNA-recognition module (RRM) RGG (arginine–glycine–glycine) motif^25^. Immunofluorescence and fractionation analysis showed that full-length G3BP1 or various G3BP1 deletion constructs present both in the cytoplasm and nucleus (Supplementary Fig. 2D, E). Here, N-terminal NTF2 of G3BP1 co-immunoprecipitated with SPOP, and so did the C-terminal RRM-RGG domain (Fig. 2d). The above findings were confirmed by reverse IP with anti-FLAG antibody (Supplementary Fig. 2A). G3BP1^RRM-RGG^ has been identified as ribonucleic acid (RNA) binding motif; however, its binding to the SPOP does not require an association with RNA molecule (Supplementary Fig. 2F).

Interestingly, the N-terminal G3BP1^NTF2^ domain has a dimerization site^35^ that may form dimer with endogenous G3BP1, which in turn bridges binding to SPOP. In LNCaP-sgG3BP1 cell line, FLAG-G3BP1^NTF2^ was still capable of co-immunoprecipitating with HA-SPOP, indicating that G3BP1^NTF2^ interacts with SPOP independent of G3BP1 homo-dimerization (Supplementary Fig. 2B). Furthermore, both the NTF2 and RRM-RGG domains of G3BP1 bind to the MATH domain of SPOP (Fig. 2e). In fact, several clinically relevant SPOP mutants have been identified and we observed that G3BP1 can interact with SPOP^WT^ but not with SPOP^F133V^ and SPOP^F102C^ (Fig. 2g).

Next, we performed proximity ligation assay (PLA) to investigate the interaction of identified domains of G3BP1 with SPOP in the nuclei of 22Rv1 cells. As shown in Fig. 2f, ectopically expressed G3BP1, G3BP1^NTF2^, and G3BP1^RRM-RGG^ showed 80%, 75%, and 70% PLA positive signals, whereas FLAG-G3BP1^PxxP^ had only a 10% signal. Thus, G3BP1, G3BP1^NTF2^, and G3BP1^RRM-RGG^ are in close proximity with SPOP in the nuclei of 22Rv1 cells, consistent with their physical associations (Fig. 2e). Immunofluorescence analysis confirmed the expression (FLAG-tagged) of ectopically expressed G3BP1 and its various deletion constructs (Fig. 2f). Collectively, PLA and the co-immunoprecipitation studies demonstrated that G3BP1, through the NTF2 and/or RRM-RGG domains, interacts with the MATH domain of SPOP.

To begin to understand the biochemical mechanisms underlying the negative regulation of SPOP ubiquitin ligase activity by G3BP1, we assessed whether physical binding is required for G3BP1-dependent suppression of SPOP. As shown in Fig. 2h, the G3BP1^NTF2^ or G3BP1^RRM-RGG^ domains were sufficient to reduce ubiquitination of TRIM24, similar to that of full-length G3BP1 (Fig. 1b). Overall, these studies provide compelling evidence of a distinctive regulatory G3BP1-SPOP ubiquitin signaling axis that restricts the threshold activity of the SPOP ubiquitin ligase.

### Increased accumulation of G3BP1 corelates with tumor aggressiveness

**Fig. 3:**
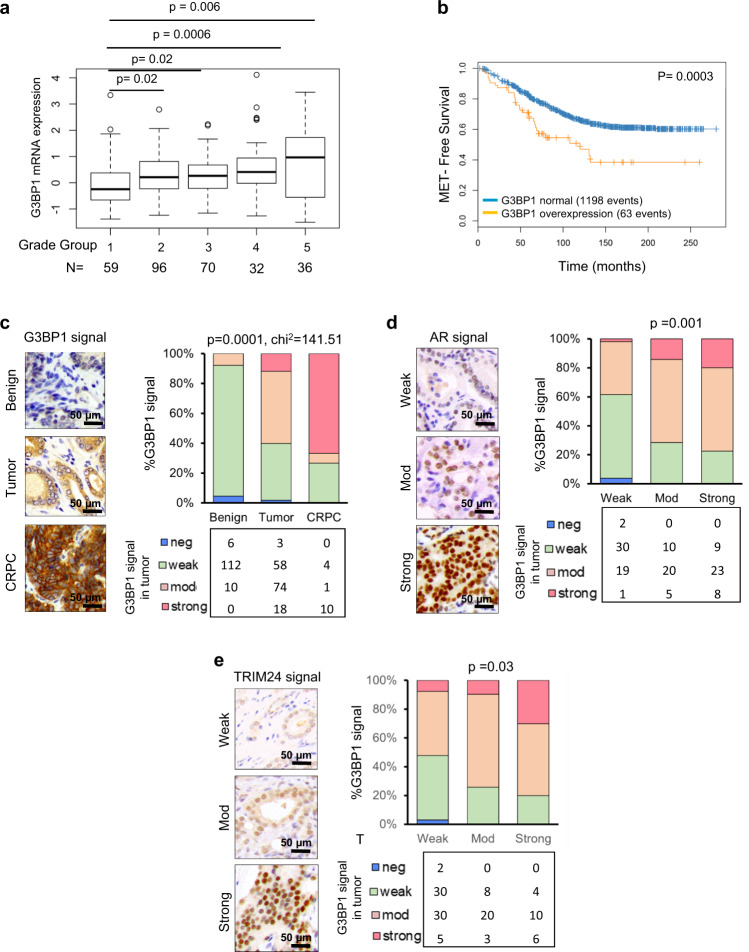
G3BP1 overexpression is associated with aggressive PCa and correlated with increased accumulation of AR and TRIM24. **a** Stepwise upregulation of G3BP1 mRNA levels with increasing tumor group grade in the TCGA cohort patients^15^. Unpaired *t*-test. In box plots, the center line represents median value, box limits represent 25% and 75% quantiles, and the top and bottom lines represent minimal and maximal values, respectively. **b** Kaplan–Meier metastasis-free survival analysis of PCa patients. Patients were stratified as low and high G3BP1 expression (log rank test, *p* = 0.0003). PCa patients undergoing radical prostatectomy and reaching metastasis postoperatively were used for analysis^37^. **c** Expression of G3BP1 in benign (*n* = 128), primary PCa tumors (Tumor) (*n* = 153), and CRPC (*n* = 15). The scale bar represents 50 µm. Paired *t-*test. *p* value is indicated in figure. **d**, **e** Correlation of G3BP1 protein and nuclear staining of AR (**d**) and TRIM24 (**e**). Paired *t-*test. The scale bar represents 50 µm. For **c**–**e**, 60 benign prostate tissues, 73 localized prostate cancers, and 16 metastatic castration-resistant prostate cancer tissues were used.

To evaluate the expression and contributions of G3BP1 in prostate cancer tissue, we analyzed TCGA RNA sequencing (RNA-seq) data from 498 patient samples and observed that G3BP1 expression is high in higher prostate cancer Grade Group^36^ (Fig. 3a). We evaluated the probability of metastasis-free survival of primary 1626 PCa patients stratified based on low or high G3BP1 group using genome-wide microarray gene expression data from a clinically available prognostic assay (Decipher; GenomeDx Biosciences, Vancouver, BC, Canada)^37^. Patients with “high” G3BP1 expression showed a lesser chance of metastasis-free survival (Fig. 3b).

Given the fact that G3BP1 is an SPOP-interacting protein, we evaluated the prognostic impact of G3BP1 in benign, primary Pca and castration-resistant prostate cancer (CRPC) by tissue microarray (TMA) and immunohistochemistry in 153 independent cases collected at the Weill Cornell Pathology tumor bank. G3BP1 expression gradually increased in tissue samples of benign to primary PCa tumors and was likely most abundant in CRPC (Fig. 3c; *p* < 0.001, *χ*^2^ test) suggesting that G3BP1 overexpression is associated with more aggressive disease across the clinical spectrum of prostate cancer. Notably, there were only a limited number of CRPC cases available in our PCa TMAs as acquiring CRPC samples often requires metastatic biopsy, thus, future study will assess G3BP1 expression in larger cohort of CRPC as prospective collection becomes more standard. Consistent with these findings, an increased G3BP1 protein levels in TMAs significantly correlated with the SPOP substrates AR (*p* = 0.001) and its co-activator TRIM24 (*p* = 0.03) (Fig. 3d, e respectively). Notably, G3BP1 protein levels were found to be high in PCa tumors featuring either SPOP^WT^ or SPOP^Mut^ (Supplementary Fig. 3), indicating that PCa-associated G3BP1 overexpression is independent of SPOP mutation status. Overall, our findings confirm that G3BP1 is abundantly expressed in aggressive PCa samples and strongly associated with the accumulation of SPOP substrates AR and TRIM24.

### G3BP1 deregulates oncogenic pathways and activates AR signaling

**Fig. 4:**
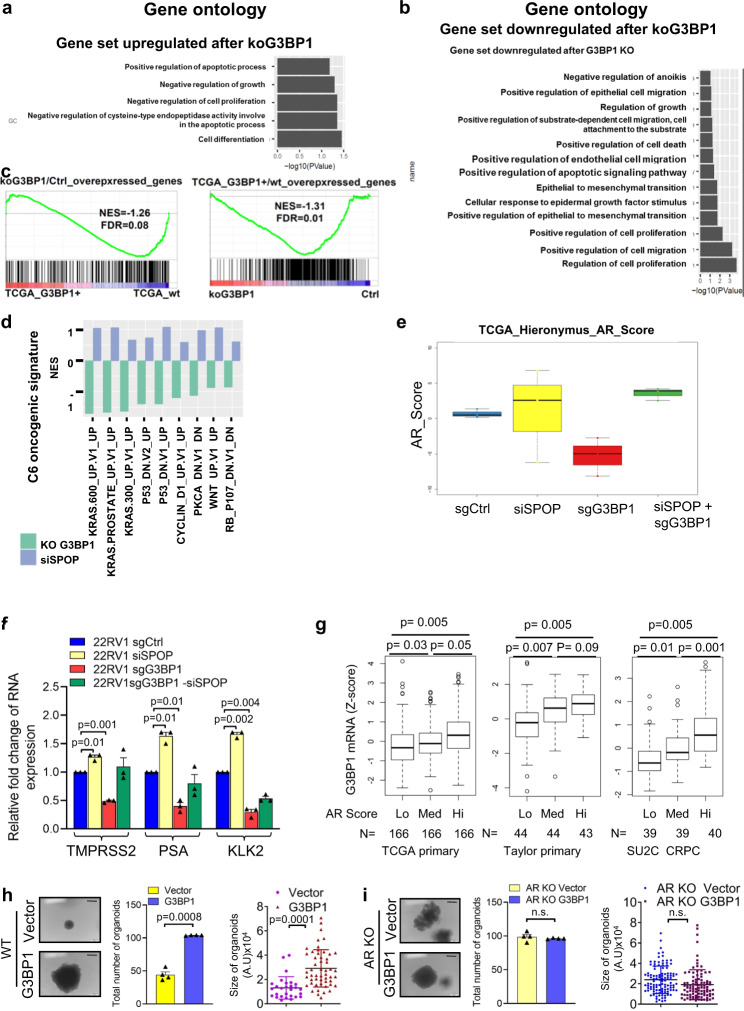
G3BP1 deregulates potential oncogenic pathways and activates AR-mediated signaling. **a**, **b** Enriched signaling pathways of upregulated genes (**a**) and downregulated genes (**b**) after G3BP1 knockout (KO) by Gene Ontology analysis. **c** GSEA analysis of G3BP1 transcriptional signature from current study and TCGA cohort. **d** Distinct enriched oncogenic signatures between G3BP1 KO and siSPOP samples compared to normal samples via GSEA. **e**, **f** 22Rv1-sgCtrl and 22Rv1-sgG3BP1 cells were transfected with non-targeted or SPOP siRNA and designated as sgCtrl, siSPOP, sgG3BP1, and sgG3BP1 + siSPOP. **e** Inverse correlation and complementation of AR score^39^ in distinct genotypes. *n* = 3 biologically independent samples. In box plots, the center line represents median value, box limits represent 25% and 75% quantiles, and the top and bottom lines represent minimal and maximal values, respectively. **f** RT-qPCR for AR target genes, TMPRSS2, PSA, and KLK2 as indicated. Error bars ±S.E.M Paired *t*-test. **g** G3BP1 mRNA levels showed a stepwise upregulation with increasing AR output scores in different PCa cohort patients [TCGA, Provisional 2019^77^; Taylor et al.^64^; SU2C, Robinson et al.^15^]. Unpaired *t*-test. *p* values are indicated in figure. In box plots, the center line represents median value, box limits represent 25% and 75% quantiles, and the top and bottom lines represent minimal and maximal values, respectively. **h** DOX-inducible empty vector (Ctrl) and pCW57.1-FLAG-G3BP1 (overexpressed) stable lines were generated from wild-type mPECs. *n* = 28 (Ctrl) and *n* = 52 (G3BP1 overexpressed) organoids examined over three independent experiments. Paired *t*-test (**i**) DOX-inducible empty vector (Ctrl) and pCW57.1-FLAG-G3BP1 (overexpressed) stable lines were generated from AR KO mPECs. *n* = 109 (Ctrl) and *n* = 109 (G3BP1 overexpressed) organoids examined over three independent experiments. Photomicrographs of representative mouse prostate organoids (scale bar, 200 µm) from the indicated mouse genotype following quantification of organoids (number and size). Unpaired *t*-test, Data are average of three independent experiments. Error bars, ±S.E.M. Source data are provided as a Source Data file.

To provide further insight into G3BP1-mediated deregulation of cellular signaling pathways, we performed RNA-seq of 22Rv1 cells after G3BP1 knockout or transient SPOP knockdown and compared them with control cells to identify changes in transcriptional programs. We used siRNA to silence SPOP transiently (Supplementary Fig. 4A), as 22Rv1 cells could not survive following constitutive knockout or knockdown of SPOP. In Heatmap (Supplementary Fig. 4B), control sets showed reduced number of downregulated genes upon G3BP1 KO or SPOP KD. Also, control sets showed enhanced number of upregulated genes upon G3BP1 KO or SPOP KD. A fraction of these genes exhibits inverse correlation when analyzed G3BP1 KO and SPOP KD as shown in Fig. 4d supporting that G3BP1 is a negative regulator of SPOP ubiquitin ligase. Gene ontology analysis using DAVID^38^ revealed that upon G3BP1-KO, genes that were downregulated included those for cell migration, proliferation, epithelial to mesenchymal transition, and cellular response to epidermal growth factor stimulus and growth regulation. Conversely, several genes involved in apoptosis were upregulated upon G3BP1-KO, indicating potentially important roles of G3BP1 in PCa (Fig. 4a, b). Here, we also observed that gene sets involved in a G3BP1-overexpressed TCGA cohort^16^ showed significantly negative enrichment in 22Rv1-sgG3BP1 cells, indicating the existence of similar transcriptional features (Fig. 4c). An unbiased GSEA analysis of G3BP1-knockout data nominated multiple deregulated pathways (Fig. 4d), and these pathways further confirmed an inverse correlation between G3BP1-KO and SPOP-KD. Overall, these findings indicate that multiple oncogenic pathways are deregulated upon G3BP1 depletion and establish an inverse correlation between G3BP1 and SPOP.

Next, we determined AR signaling using a previously defined AR target gene set^39^, which we found to be decreased in G3BP1-KO cells and restored upon SPOP-KD (Fig. 4e). Further, we found that gene sets involved in AR downregulation showed significant enrichment (FDR = 0.04) in 22Rv1-sgG3BP1 cells (Supplementary Fig. 4D). Expression of AR target genes, such as TMPRSS2^40^, KLK2, and KLK3 (i.e., PSA) (Fig. 4f and Supplementary Fig. 4E), was reduced in G3BP1-KO (Supplementary Fig. 4C) cells but was restored by SPOP-KD (Supplementary Fig. 4C). We analyzed three PCa data sets and consistently observed that G3BP1 expression directly correlated with AR signaling (Fig. 4g). These data support the conclusion that G3BP1 restrains SPOP function and thus activates its substrate AR. To avoid misinterpretation due to multiple pre-existing genetic alterations, present in PCa cell lines and patient samples, we performed genetic ablation of G3BP1 in primary mPECs (murine prostate epithelial cells). Here, knockdown of G3BP1 in primary mPECs showed downregulated FKBP5 transcripts, but upregulated IGFBP3 (Supplementary Fig. 4F), a marker of decreased AR activity^41^. These results validated the stimulatory effects of G3BP1 and repressive effects of SPOP, respectively, on AR signaling.

Immunoblot analysis of organoids cultured at different concentrations (0, 1, and 10 nM) of dihydrotestosterone (DHT) revealed that knockdown of G3BP1 led to reduced levels of AR, TRIM24 and FKBP5 at 0 nM and 1 nM DHT (Supplementary Fig. 4G). Interestingly, increased expression of G3BP1, AR, TRIM24, and FKBP5 at 10 nM DHT in G3BP1-KO mPECs indicates that DHT induces increased expression of G3BP1 and its downstream AR signaling (Supplementary Fig. 4G). The observed data thus validates the activation of AR-driven signaling by the G3BP1-SPOP axis. Furthermore, consistent with earlier findings, G3BP1 overexpression in WT mPECs showed significantly (*p* < 0.001) enhanced organoid formation (Fig. 4h). To ascertain that AR is the main target deregulated by the G3BP1**-**SPOP ubiquitin signaling axis, we overexpressed G3BP1 in AR-knockout mPECs (AR-KO G3BP1), and its overexpression was confirmed by immunoblot analysis (Supplementary Fig. 4H, I). Importantly, we observed no change in size or number (*p* = n.s) of organoids between the AR-KO and AR-KO G3BP1 mPECs (Fig. 4i), which establishes a role for AR in G3BP1-SPOP ubiquitin signaling. Overall, these results provide compelling evidence that AR plays a major role in G3BP1-mediated downstream signaling in prostate epithelial cells.

### AR activates G3BP1 transcription to amplify AR signaling

**Fig. 5:**
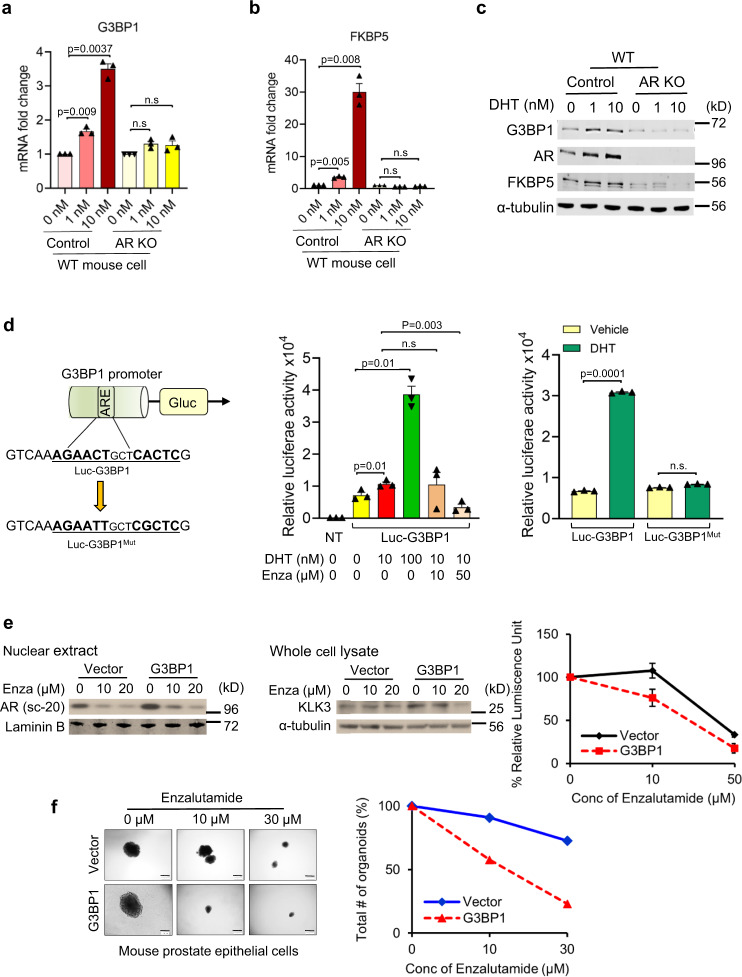
AR activates G3BP1 transcription to maintain a feed-forward amplification of AR signaling, resulting in sensitization of G3BP1^high^ PCa cells to AR-targeted drugs. **a**, **b** Control and AR KO mPEC-derived organoids were treated with DHT at the indicated concentrations. Expression of G3BP1 (**a**) and the AR-target gene FKBP5 (**b**) was quantitatively measured by RT-qPCR. *n* = 3 biologically independent experiments. Error bars, ±S.E.M. *p* values are indicated in the figure. **c** Immunoblotting of WCL derived from control and AR KO organoids treated with vehicle or DHT for G3BP1, AR, FKBP5, and α-tubulin (loading control). *n* = 3. (**d** left) Schematic diagram of luciferase reporter constructs showing sequence of wild-type (in Luc-G3BP1) and mutant ARE (in Luc-G3BP1^Mut^) in G3BP1 promoter region. (**d**, middle) 22Rv1 cells were transfected with G3BP1 promoter luciferase reporter constructs and treated with DHT alone or in combination with enzalutamide (Enza) as indicated. Gaussia Luciferase activity was determined and normalized against secreted Alkaline Phosphatase activity. NT, no transfection. (**d** right) 22Rv1 cells were transfected with G3BP1 promoter luciferase reporter (Luc-G3BP1) and G3BP1 promoter luciferase reporter where ARE site is mutated (Luc-G3BP1^Mut^) constructs and treated with 100 nM DHT. Gaussia Luciferase activity was determined and normalized against secreted Alkaline Phosphatase activity. *n* = 3 biologically independent experiments. *p* value is indicated in figure. Error bars, ±S.E.M. **e** Immunoblots of nuclear extract (left) and whole cell lysate (middle) derived from DOX-inducible empty vector (Ctrl) and pCW57.1-FLAG G3BP1 (overexpressed) 22Rv1 cells treated with enzalutamide for 24 h with the indicated concentrations for the indicated proteins. *n* = 3. Detection of growth inhibition of Ctrl and G3BP1 overexpressed 22Rv1 cells (right) in the presence of enzalutamide with the indicated concentrations using Cell Titer Glow assay. *n* = 3 biologically independent experiments. Error bars, ±S.E.M. **f** Photomicrographs of representative mouse prostate organoids (scale bar, 200 µm) from the indicated mouse genotype treated with enzalutamide with the indicated concentrations following quantification of organoid number. A representative result from more than three independent experiments is shown. Source data are provided as a Source Data file. WCL whole cell lysate.

Our data showed that DHT induces increased expression of G3BP1 and its downstream AR signaling (Supplementary Fig. 4G), which indicates a potential feed-forward amplification of the AR-G3BP1-AR loop. Notably, like the AR target gene FKBP5 (Fig. 5b), increased expression of G3BP1 was detected with an increased dose of DHT in WT, but not in AR KO mPECs at both the transcript (Fig. 5a) and protein (Fig. 5c) levels, suggesting the potential feed-forward amplification loop. Interestingly, we found the presence of an AR binding site within the 1600 bp G3BP1 promoter region. As shown in Fig. 5d left, we constructed the luciferase reporter constructs driven by either a 1600 bp region of wild-type G3BP1 promoter or the one in which ARE (androgen-responsive element) site (ARE^Mut^) was mutated (AGAA**C**TgctC**A**CTCG has been changed to AGAA**T**TgctC**G**CTCG). WT and ARE^Mut^ luciferase reporters were transfected into 22Rv1 cells and treated with DHT or vehicle for 24 h (Fig. 5d middle and right). As predicted, DHT treatment significantly transactivated the wild type, but not the ARE^Mut^, promoter, suggesting that DHT induced G3BP1 transcription at least in part through this ARE site. Overall, these results demonstrate that G3BP1 is an AR regulated gene.

Enzalutamide, a second-generation antiandrogen, binds to AR and blocks its nuclear translocation and DNA binding^42^. As predicted, treatment with enzalutamide significantly decreased G3BP1 promoter activity in a dose-dependent manner (Fig. 5d middle), suggesting that ARE-mediated AR binding to the G3BP1 promoter activated G3BP1 transcription. Given the findings that AR is a major signaling pathway upregulated by the G3BP1-SPOP axis, we wanted to investigate the impact of enzalutamide on G3BP1-overexpressed cells. Here, we generated G3BP1-overexpressing 22Rv1 (22Rv1-G3BP1) cells (Supplementary Fig. 5A). Our data showed that AR and KLK3 are downregulated in 22Rv1-G3BP1 (vs 22Rv1-vector control) cells treated with enzalutamide (Fig. 5e) and apalutamide (Supplementary Fig. 5C), whereas the effects of bicalutamide (Supplementary Fig. 5B) were minimal. This finding is because of functional differences between enzalutamide or apalutamide and bicalutamide. Binding of enzalutamide or apalutamide to AR causes inefficient nuclear translocation of AR, completely inhibit DNA binding and coactivators recruitment; whereas binding of bicalutamide to AR preserves nuclear translocation of AR; and DNA binding and coactivators recruitment occurs when AR is overexpressed^43,44^.

### G3BP1 sensitizes prostate cancer cells to AR-targeted drugs

We sought to define the survival of G3BP1-overexpressed cells in the presence of AR antagonists. 22Rv1-vector and 22Rv1-G3BP1 cells were incubated without or with 10 or 50 μM of each AR antagonist for 6 days. 22Rv1-G3BP1 cells showed a dose-dependent reduction in viability following treatment with androgen receptor inhibitors (Fig. 5e right and Supplementary Fig. 5B, C). In this regard, we also observed similar dose-dependent reduction in viability of G3BP1-overexpressed C4-2B and LNCaP-95 cells upon treatment with enzalutamide (Supplementary Fig. 5E, F). Interestingly, we observed that the suppressive effect of enzalutamide and apalutamide was more robust than bicalutamide. No appreciable cell death (7–10%) was seen after only 24 hours of treatment with enzalutamide under similar experimental conditions (Supplementary Fig. 5D), indicating no immediate cellular toxicity. An organoid formation assay using mPECs revealed that a reduced amount of enzalutamide effectively inhibits organoid formation of G3BP1-overexpressed cells relative to the control (Fig. 5f). Overall, these results demonstrated that AR enhances G3BP1 expression to maintain a feed-forward amplification of AR signaling and sensitizes G3BP1^high^ PCa to AR-targeting drugs. Importantly, these findings aid in our current knowledge that G3BP1 overexpression may offer a prognostic means for the effectiveness of enzalutamide treatment.

### G3BP1-SPOP axis controls cellular migratory and invasive potential

**Fig. 6:**
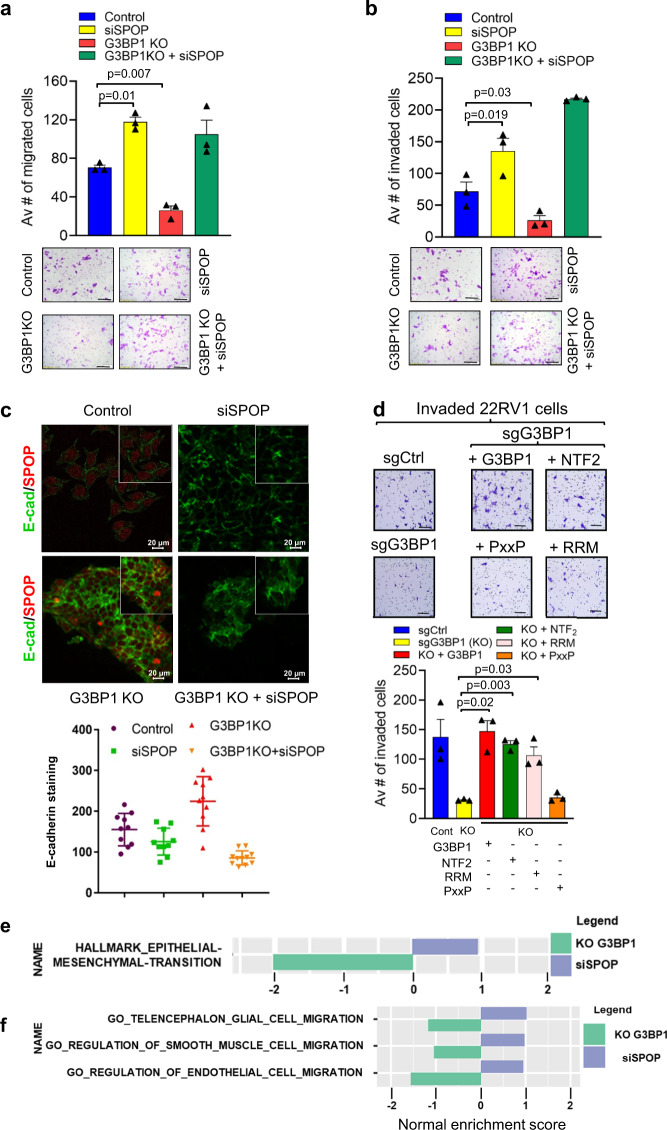
G3BP1-SPOP ubiquitin signaling axis controls migratory and invasive potential of prostate cancer cells. **a**, **b** 22Rv1-sgCtrl or 22Rv1-sgG3BP1 cells were transfected with non-targeted or SPOP siRNA and designated as Ctrl, siSPOP, G3BP1 KO (sgG3BP1), and G3BP1 KO + siSPOP (sgG3BP1**-**siSPOP). Quantification and representative images (×10 magnification) of **a** migrated and **b** invaded cells. Error bars, ±S.E.M. *n* = 3 biologically independent experiments. Paired *t*-test (**c**) representative immunofluorescence images of E-cadherin expression in 22Rv1 cells. scale bar, 20 µm. Quantification of E-cadherin staining represented as arithmetic mean intensity. *n* = 3 biologically independent experiments. Error bars, ±S.E.M. **d** Representative images (×10 magnification) of invasive 22Rv1-sgG3BP1 cells transfected with the indicated G3BP1 constructs in matrigel invasion assays. Quantification of data averaged on *n* = 6 biologically independent experiments. Error bars, ±S.E.M. Paired *t*-test. *p* value is indicated in figure. **e**, **f** GSEA analysis of G3BP1 KO and siSPOP cells compared to control samples based on epithelial and mesenchymal transition-related signatures (**e**), and migration-related signatures (**f**). Source data are provided as a Source Data file.

In agreement with a previous report that SPOP suppresses invasive potential^11^, we found that the transient knockdown of SPOP significantly increased invasion (by 87%, *p* = 0.009) and migration (by 89%, *p* = 0.03) (Fig. 6b, a, yellow bar) compared to control 22Rv1 cells (Fig. 6b, a, blue bar). Next, we tested whether negative regulation of SPOP ubiquitin ligase activity by G3BP1 would promote migration and invasion of PCa cells. Upon knockout of G3BP1, we detected significantly reduced migratory (by 61%, *p* = 0.01) and invasive (by 71%, *p* = 0.01) potential of 22Rv1 cells (Fig. 6a, b, red bar) as compared to the control (Fig. 6a, b, blue bar). Silencing of SPOP in 22Rv1-sgG3BP1 cells effectively reversed the migration and invasion deficiency, indicating that G3BP1-dependent cell migration and invasion are mediated by SPOP (Fig. 6a, b, green bar). A similar phenotype was detected after transient knockdown of G3BP1 and/or SPOP in 22Rv1 cells, confirming that the observed results are not influenced by a clonal effect of 22Rv1-sgG3BP1 stably transfected cells (Supplementary Fig. 6A–C).

**Fig. 7:**
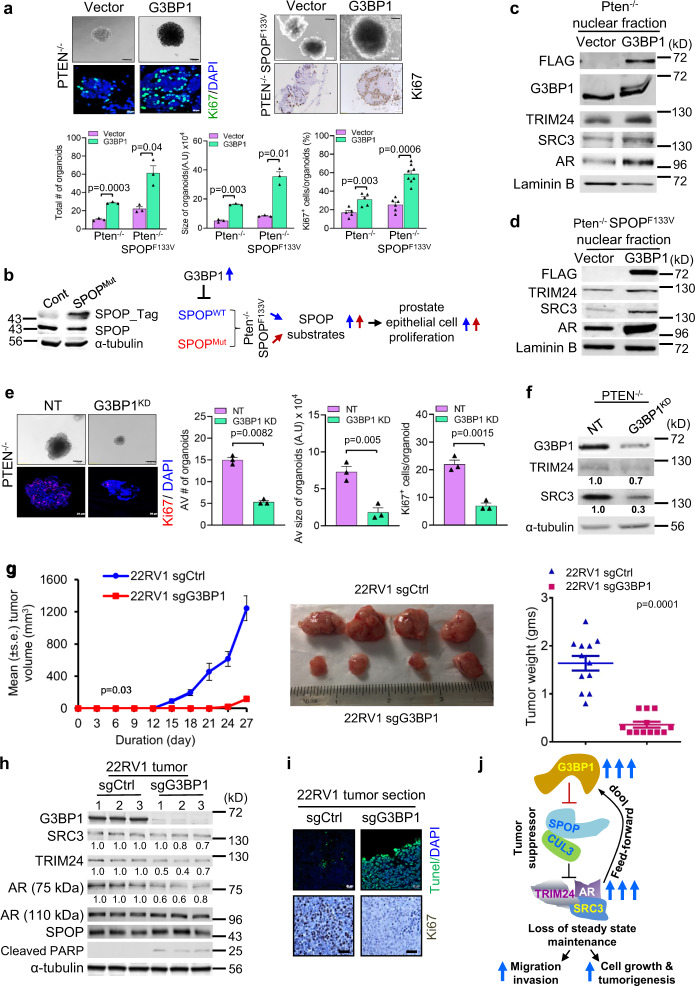
G3BP1 suppresses SPOP function to facilitate organoid formation and tumorigenesis. **a** DOX-inducible empty vector (Ctrl) and G3BP1 (overexpressed) stable lines were generated from *Pten*^*−/−*^ (upper left) and *Pten*^*−/−*^*SPOP*^*F133V*^ (upper right) mPECs. Photomicrographs of representative mouse prostate organoids (scale bar, 200 μm) and Ki67 (scale bars; left 20 μm, right, 200 μm) staining from the indicated mouse genotype following quantification of organoids number (lower left *n* = 3) and size (lower middle, *n* = 3) and Ki67 positive cells (lower right) *n* = 3 biologically independent experiments. Error bars, ±S.E.M. Paired *t*-test. (**b** left) Immunoblot analysis showing WT-SPOP and FLAG**-**SPOP^F133V^ expression. α**-**tubulin served as the loading control. *n* = 3. (**b** right) Schematic representation of SPOP^F133V^ mPECs showing the expression of SPOP^F133V^ and WT SPOP. Upon G3BP1 overexpression, SPOP^F133V^ mPECs exhibit an additive effect on the oncogenic phenotype mediated by SPOP^F133V^ (defective G3BP1 binding) and the G3BP1**-**SPOP axis. **c**, **d** Immunoblot analysis of SPOP substrates in control and G3BP1-overexpressed *Pten*^*−/−*^ (**c**, *n* = 3) or *Pten*^*−/−*^*SPOP*^*F133V*^. (**d**, *n* = 3) mPECs. **e** Photomicrographs of representative mouse prostate organoids (scale bar, 200 µm) and Ki67 (scale bars, 20 μm) staining from the indicated mouse genotype [DOX-inducible non-targeted (NT) and G3BP1 shRNA (G3BP1^KD^)] following quantification of organoids (number and size) and Ki67 positive cells *n* = 3 biologically independent experiments. Error bars, ±S.E.M. Paired t-test. **f** Representative immunoblots showing SPOP substrates in *Pten*^*−/−*^ mPECs. α-tubulin served as loading control. *n* = 3. **g** 22Rv1-sgCtrl or 22Rv1-sgG3BP1 cells were injected into the flanks of nude mice. (left) Relative tumor growth as fold increase in volume relative to time. (middle) Representative photographs of xenograft tumors. (right) Tumor weights at 8-weeks. *n* = 12 biologically independent mice. Error bars, ±S.E.M. Paired *t*-test. **h** Immunoblots of tumor lysates in triplicates from sgCtrl and sgG3BP1 groups of the indicated proteins. α-tubulin served as the loading control. *n* = 3. **i** Photomicrographs of Ki67 and TUNEL staining of sgCtrl and sgG3BP1 xenograft tumors. *n* = 3. **j** Proposed role of G3BP1 that restricts the threshold activity of the Cul3^SPOP^ ubiquitin ligase and feed forward amplification of G3BP1-SPOP-AR ubiquitin signaling axis. All *p* values are indicated in figure. Source data are provided as a Source Data file.

It is noteworthy that migration and invasion assays were carried out within 48 h, and we did not observe significant changes in the proliferation detected by cell cycle analysis and CFSE (Carboxyfluorescein succinimidyl ester) assays (Supplementary Fig. 6D), nor in cell viability as detected by apoptosis assays (Supplementary Fig. 6E-F) due to shG3BP1 induction. However, initiation of apoptosis^45^ was confirmed by significantly increased caspase 3/7 activity in 22Rv1-shG3BP1 after 48 h (Supplementary Fig. 6E); this increase supports the finding shown in Fig. 7i: that 22Rv1-sgG3BP1 tumors exhibit more TUNEL-positive cells. These results indicate that genetic ablation of G3BP1 initiates the apoptotic program as early as 48 h; however, it triggers cell death during a later time (week 3–4). E-cadherin is an epithelial cell marker that acts as a potent suppressor of invasion^46^. Immunofluorescence analysis revealed enhanced expression of E-cadherin in 22Rv1-sgG3BP1 cells compared with control cells (Fig. 6c). Silencing of SPOP in 22Rv1-sgG3BP1 cells reduced E-cadherin accumulation, indicating that G3BP1 plays a role in suppressing epithelial phenotypes (Fig. 6c).

As expected, trans-well migration (Supplementary Fig. 6H) and matrigel invasion (Fig. 6d) of 22Rv1-sgG3BP1 cells (vs 22Rv1-sgCtrl) were significantly decreased by 75% (*p* = 0.03) and 77% (*p* = 0.02) respectively. Consistent with the biochemical findings described earlier (Fig. 2d, f, g), here, we observed that overexpression of G3BP1 or the G3BP1^NTF2^ or G3BP1^RRM-RGG^ domains in 22Rv1-sgG3BP1 cells (Supplementary Fig. 6G) restored the migratory potential by 71% (*p* < 0.001), 67% (*p* = 0.009) and 57% (*p* = 0.004), respectively (Supplementary Fig. 6H), and the invasive potential by 79% (*p* = 0.002), 75% (*p* < 0.001), and 71% (*p* = 0.005) respectively (Fig. 6d). These findings confirmed that the NTF2 and RRM-RGG domains of G3BP1 interact with and negatively regulate the ubiquitin ligase function of endogenous SPOP, and that this interaction is sufficient for enhanced cellular migration and invasion of PCa cells. However, G3BP1^PxxP^ and G3BP1^Ac-PXXP^ do not bind to SPOP and expression of these domains in the 22Rv1-sgG3BP1 cells did not rescue migratory or invasive property indicating that domains that are incapable of binding to SPOP are not involved in G3BP1 driven cell migration and invasion (Supplementary Fig. 6H and Fig. 6d).

We performed RNA-seq in 22Rv1-sgG3BP1 (vs control) and 22Rv1-siSPOP (vs control). Gene set enrichment analyses (GSEA) identified a negative association of silencing SPOP with G3BP1 loss when compared hallmark EMT gene sets (Fig. 6e) and migratory potential (Fig. 6f). Taken together, these findings clearly demonstrated that G3BP1 regulates a molecular program where the SPOP ubiquitin ligase function is compromised, resulting in increased accumulation of SPOP substrates that drive migration and invasion of PCa cells.

### G3BP1-SPOP axis controls organoid formation of primary prostate cells

Organoids from mPECs recapitulate features of prostate histology^12^, including growth as multilayered structures with expression of CK5 in basal and CK8 in luminal layers of cells (Supplementary Fig. 7A)^12,33,41,47^. As shown in Fig. 7a upper left, G3BP1-overexpressing *Pten*^*−/−*^ cells (Supplementary Fig. 7B) displayed organoids that were significantly larger in size (*p* < 0.001) and greater in numbers (*p* < 0.001) than those from cells with the empty vector control, indicating that they are more proliferative (Fig. 7a lower left and lower middle). Similar to *Pten*^*−/−*^ mPECs, G3BP1 overexpression in WT mPECs showed significantly (*p* < 0.001) enhanced organoid formation (Fig. 4h).

G3BP1^high^ occurs in both wild type and SPOP-mutated prostate cancers. To assess G3BP1-SPOP signaling in SPOP-mutant PCa, we generated *Pten*^***−/−***^*-SPOP*^*F133V*^ mPECs (Supplementary Fig. 7C) with doxycycline (DOX)-inducible G3BP1 as a model of G3BP1^high^ SPOP^F133V^ PCa. Here, we used primary *Pten*^*−/−*^ mPECs that express the SPOP^F133V^ transgene, which were shown to drive invasive PCa in vivo^12^. Importantly, *Pten*^***−/−***^*-SPOP*^*F133V*^ cells express endogenous SPOP^WT^ and mutant SPOP^F133V^ protein in a 1:1 ratio (Fig. 7b left), recapitulating what is seen with SPOP-mutated human prostate cancer^12^. It is noteworthy that G3BP1 can interact with SPOP^WT^ but not with SPOP^F133V^ (Fig. 2g). A more profound effect was observed upon G3BP1 overexpression in *Pten*^*−/−*^*-SPOP*^*F133V*^ (Fig. 7a upper right). Here, we observed formation of organoids ~4 times greater in size and ~1.6 times greater in number with *Pten*^*−/−*^*-SPOP*^*F133V*^-G3BP1 than *Pten*^*−/−*^-G3BP1 (Fig. 7a lower left and lower middle). These findings confirm that G3BP1 overexpression facilitates a severe-disease phenotype by adding the functional inhibitory effect of G3BP1 on the SPOP^WT^ allele on top of the existing tumor-promoting effect of G3BP1-resistant SPOP^F133V^ (Fig. 7b right). As expected, an increase in Ki67^+^ cells were observed in organoids generated from *Pten*^*−/−*^*-*G3BP1 (38%) when compared with controls (18%) (Fig. 7a upper left). A further increase in Ki67^+^ cells (60%) (Fig. 7a upper right) was observed in the organoids generated from *Pten*^*−/−*^*-SPOP*^*F133V*^-G3BP1 cells when compared with *Pten*^*−/−*^-G3BP1 (Fig. 7a lower right). In organoids generated from each cell type, G3BP1 overexpression (Fig. 7c, d) resulted in upregulation of the SPOP substrates AR, TRIM24, and SRC3 (Fig. 7c, d and Supplementary Fig. 7D). We conclude that G3BP1^high^ and SPOP mutations are two oncogenic means that function either independently or jointly to disable the tumor-suppressive role of SPOP and promotes prostate tumor progression.

Next, we attempted to determine whether silencing of G3BP1 can reinvigorate the SPOP function, so that SPOP can degrade its substrates that are responsible for PCa progression. An organoid formation assay revealed that stable knockdown of G3BP1 in *Pten*^*−/−*^ mPECs (Fig. 7e) significantly reduced organoid formation efficiency (by 67%, *p* < 0.0001) and size (by 72%, *p* < 0.001) compared to the control (Fig. 7e). These results were corroborated with Ki67 staining, as G3BP1 knockdown organoids showed 63% fewer Ki67^+^ cells than the control (Fig. 7e). Knockdown of G3BP1 did not change G3BP2 at the RNA (Supplementary Fig. 7F) and protein (Supplementary Fig. 7E, H) levels, thus confirming the absence of compensation. As expected, immunoblot analysis showed that TRIM24 and SRC3 were downregulated upon knockdown of G3BP1 (Fig. 7f). Similarly, organoids from stable G3BP1 knockdown in WT mPECs showed reduced (~50%) Ki67^+^ cells (Supplementary Fig. 7G); decreased expression of TRIM24 and SRC3 (Supplementary Fig. 7H); and development of a significantly lower number (*p* = 0.03) and smaller size (*p* < 0.0001) of organoids as compared to the WT mPEC control (Supplementary Fig. 7G). Thus, the observed effect is due to G3BP1 knockdown, rather than from downregulation of the activated PI3K pathway resulting from the *Pten*^*−/−*^ background. These results confirm that G3BP1 induces a highly proliferative phenotype by suppressing CUL3^SPOP^ function, and depletion of G3BP1 partially reversed this phenotype.

### Silencing of G3BP1 restores SPOP function and reduces tumor formation

As silencing of G3BP1 was observed to reduce migration, invasion, and organoid formation, we sought to investigate the tumorigenic potential of G3BP1. Non-targeted (sgCtrl) and G3BP1 knockout (sgG3BP1) 22Rv1 cells were implanted subcutaneously into the flanks of two groups of male nude mice (*n* = 12). sgCtrl cells formed detectable tumors at week two, while tumors resulting from sgG3BP1 cells were detectable only after 3 weeks (Fig. 7g, left). The tumor volume generated from sgG3BP1 cells was significantly (*p* < 0.0001) smaller than tumors obtained from sgCtrl cells (Fig. 7g, left). At 4 weeks, the excised tumors generated from sgG3BP1 cells had significantly (*p* < 0.0001) reduced weight (0.36 ± 0.06 gm) compared with tumors (1.64 ± 0.15 gm) obtained from sgCtrl cells (Fig. 7g, right). In vivo G3BP1 expression was confirmed at the protein level in excised tumors generated from control cells and was undetectable in excised tumors generated from 22Rv1-sgG3BP1 cells (Fig. 7h). Immunoblot analysis of excised tumors showed reduced expression of SRC3 and TRIM24 in the sgG3BP1 tumors compared to the sgCtrl (Fig. 7h). As expected, enhanced ubiquitination of TRIM24 and SRC3 was observed in sgG3BP1 22Rv1 cells. This data corroborates well with the reduced expression of TRIM24 and SRC3 in sgG3BP1 xenograft tumor samples (Supplementary Fig. 7I). AR (110 kDa) did not show a significant change in the tumor samples analyzed; however, we observed reduced expression of the AR variant (75 kDa) in excised tumors generated from sgG3BP1 cells (Fig. 7h), suggesting reduced AR signaling, possibly because of reduced formation of the AR variant heterodimer. These results well correlated with reduced proliferation as detected by Ki67^+^ cells (Fig. 7i), enhanced cellular death as detected by TUNEL-positive staining (Fig. 7i) and the appearance of cleaved PARP (Fig. 7h) in 22Rv1-sgG3BP1 tumor samples. Overall, these results confirm that silencing of G3BP1 restores SPOP’s ubiquitin ligase function and inhibits prostate tumorigenesis.

## Discussion

In this study, we identified the G3BP1 oncoprotein as a negative regulator of CUL3^SPOP^ ubiquitin ligase. This finding uncovered a previously unrecognized means of SPOP inactivation that is independent of SPOP gene mutations and identified a unique G3BP1-SPOP-AR ubiquitin signaling axis for upregulation of the AR transcriptional network. Notably, our findings revealed a feed-forward amplification of G3BP1-SPOP-AR ubiquitin signaling via AR-dependent transcriptional activation of G3BP1, resulting in hyper-activation of the AR transcriptome (Fig. 7j). We propose that G3BP1^high^ may represent a PCa patient cohort characterized by compromised tumor-suppressive SPOP ubiquitin ligase and hyper-activated AR.

It is noteworthy that SPOP mutations are found at higher frequency in prostate adenocarcinomas (Pca) than CRPCs or NEPCs^48^. G3BP1 begins to accumulate in primary Pca, and to higher levels in CRPCs (Fig. 3c). In this regard, SPOP mutants exhibit activities in both loss-of-function (e.g., no substrate recruitment) and gain-of-function (e.g., inhibiting the remaining wild-type allele or recruitment of neo-substrates)^7–11,14–16,49^, whereas G3BP1^high^ functions to block SPOP ubiquitin ligase. While somatic SPOP mutations at the substrate-binding MATH domain are primarily prostate cancer-specific, G3BP1 overexpression (G3BP1^high^) is a much broader oncogenic signature in a wide spectrum of tumor types^25,26,50^, suggesting that suppression of SPOP by G3BP1 is likely more prevalent (20% high and 40% moderate) and is the underlying mechanism of SPOP inactivation in other tumor types. Future studies should address whether it is the G3BP1-mediated suppression of SPOP or other SPOP-independent G3BP1 functions, such as stress granule formation that facilitates PCa progression to higher grade tumors.

We identified G3BP1 as an upstream regulator of CUL3^SPOP^ ubiquitin ligase. It is conceivable that binding of G3BP1 to CUL3^SPOP^ may preclude substrate binding or result in conformational change of SPOP that subsequently interferes with or blocks transfer of ubiquitin to substrates. Interestingly, we observed monoubiquitination of G3BP1 by SPOP (Supplementary Fig. 1F, lane 4). We have further demonstrated that both the N-terminal NTF2 and C-terminal RRM-RGG domain of G3BP1 are capable of interacting with the MATH domain of SPOP and exerting the function of G3BP1 to confer on cells an oncogenic phenotype. To this end, SPOP functions as a dimer; and a higher order configuration consisting of oligomerized SPOP has also been identified, suggesting concerted action of multivalent CRL3^SPOP^ macromolecular machinery in ubiquitinating SPOP substrates^34,51,52^. Two binding domains at the N- and C-terminus of G3BP1 would enable the assembly of a multivalent complex that restricts the threshold activity of the dimeric or oligomeric SPOP ubiquitin ligase machinery.

Our investigation of global changes in the cellular transcriptome revealed that the G3BP1-SPOP axis mainly accelerates AR signaling and enhances AR target gene expression. Overexpressing G3BP1 stimulated organoid formation in AR^+^ prostate epithelial cells (Fig. 4h), but not AR knockout cells (Fig. 4i), indicating that G3BP1 acts primarily by activating the AR signaling pathway. We propose that G3BP1 overexpression defines a PCa patient cohort that exhibits high AR signaling and sensitivity to AR-targeted therapy. These findings provide a further rationale for testing antiandrogen drugs on G3BP1^high^ PCa cells for better sensitivity and improvement of therapeutic efficacy. G3BP1 expression promotes sensitivity to enzalutamide and is also associated with disease progression. The process of disease progression, especially in earlier, untreated disease, is often fundamentally distinct from therapeutic resistance. Importantly, certain biomarkers and disease can be prognostically unfavorable while still being predictive for response to specific therapeutic regimens. Recent reports have shown that SPOP mutations may be associated with improved response to androgen targeting therapies in metastatic prostate cancer^53^, despite the fact that this is a cohort of patients that had already progressed to universally lethal disease. Given our data that G3BP1 expression may partially phenocopy SPOP mutation, it may drive a similar aggressive but androgen-responsive phenotype. It is noteworthy that the oncogenic role of G3BP1 in tumorigenesis is not confined to AR signaling, as G3BP1 also restricts the function of SPOP ubiquitin ligase, which regulates several other signaling molecules, including BRD^54^, PD1^55^, and ERG^56^, that warrant further investigation.

Our investigation has revealed the inverse correlation of G3BP1 and SPOP in AR signaling, proliferation, migration, invasion, and tumor growth of PCa, indicating that G3BP1 regulates a molecular program in which SPOP ubiquitin ligase function is compromised, thus increasing the abundance of SPOP substrates that influence cellular functions. The importance of the G3BP1-SPOP axis was further highlighted by the use of conditional *Pten*^*−/−*^ *SPOP*^*F133V*^ mouse model systems^12^. Here, upon G3BP1 overexpression, *Pten*^*−/−*^*-SPOP*^*F133V*^ cells showed severe phenotypic abnormalities. Given that in PCa the SPOP mutations are hemizygous in nature and G3BP1 binds to wild-type SPOP but not SPOP^F133V^ mutant, our results suggest that G3BP1 adds another layer of negative regulation of remaining SPOP^WT^ allele on top of the existing tumor-promoting effect of SPOP^F133V^. Notably, the SPOP^F133V^ mutant is refractory to G3BP1 inhibition due to loss of biding. Altogether, our findings show the feasibility of exploiting the G3BP1-mediated suppression of SPOP’s ubiquitin ligase function for intervention against prostate tumorigenesis. Future investigation will also shed light on SPOP independent function of G3BP1. We posit that ablation of G3BP1 would free SPOP to perform its regular function of restricting the oncogenic potential. As expected, knockout of G3BP1 freed SPOP to perform its regular function, which reduced oncogenic potential as detected by reduced formation of organoids and tumors. In line with these findings, multiple independent reports also demonstrated that G3BP1 is potentially capable of enhanced tumor formation with highly proliferative phenotypes in other cancers^24,27–29^.

Moreover, G3BP1 contributes to stress granule formation to protect mRNAs under adverse conditions; such protection is thought to be involved in PCa tumorigenesis and response to therapy^18–22^. Interestingly, we did not see co-localization of G3BP1 with SPOP in cytosolic stress granules under stress conditions (Supplementary Fig. 7J). Recent studies suggest that G3BP1 acts both in stress-dependent and independent manner as switch for the formation of different macromolecular complexes^30–32^. Collectively, a combination therapy that targets both AR and other downstream signaling pathways is expected to provide a full therapy benefit for G3BP1^high^ tumors

In summary, our current finding offers an insight into the oncogenic role of G3BP1 as an upstream regulator of the tumor suppressor SPOP. Moreover, AR directly upregulates G3BP1 transcription to further amplify G3BP1-SPOP signaling in a feed-forward manner and potentiates AR signaling and promote prostate tumorigenesis (Fig. 7j). Thus, this G3BP1^high^ constitutes a PCa patient cohort and provides an opportunity for precision therapy. Our findings will aid in improving our current understanding and will provide a rationale for targeting G3BP1 in these settings.

## Methods

### Experimental model and subject details

#### Human cell lines

HEK 293T, LNCaP, C4-2B, and 22Rv1 cells were purchased from the American Type Culture Collection (ATCC- Item # CRL-1740, CRL-3315, CRL-2505) grown on either poly-L-lysine coated plates or regular tissue culture coated plates in 5% or 10% Fetal Bovine Serum (FBS) containing RPMI-1640 and incubated at 37 °C and 5% CO_2_. LNCaP-95 cells were kindly provided by Dr. Loda’s laboratory (WCMC, NY, US) (RRID:CVCL_ZC87). Cells were passaged twice weekly or once cultures reached 75% confluency. All cultured cells were Confirmed for free of mycoplasma monthly via the highly sensitive PCR-based kit from Sigma (Cat # MP0025). Where applicable, cell line identity was validated yearly though the Human STR profiling cell authentication service provided by ATCC. Primary WT mPECs, *Pten*^*−/−*^ mPECs and *Pten*^*−/−*^*-SPOP*^*F133V*^ mPECs were generated in Dr. Barbieri’s laboratory (WCMC, NY, US). All primary mPECs were maintained in medium, and culture conditions were as previously described^12,47^.

#### CRISPR model generation

LNCaP and 22Rv1 cells (<passage 10) were transfected with All-In-One pLentiCRISPR v2/sgRNA plasmids containing either control or G3BP1 specific sgRNAs (AACGTTTGTCCTTGCTCCTG) custom designed from GenScript. Cells were selected with puromycin until resistant populations emerged, and then assayed for G3BP1 depletion using immunoblot.

#### Engineered G3BP1 overexpression model

Stable pooled populations of 22Rv1, LNCaP-95, C4-2B control, and G3BP1 overexpressed [pCW57.1-FLAG G3BP1 (overexpressed)]. cells were generated by selection using 3 μg/mL puromycin. Levels of mRNA transcripts, expression of protein, and the phenotype of cells were analyzed.

#### Engineered mouse prostate epithelial cells

Primary mouse (C57BL/6) prostate epithelial cells were isolated by dissecting 6-10-week-old WT, *Pten*^*−/−*^, and *Pten*^*−/−*^ *SPOP*^*F133V*^ mouse prostates. Cells were infected with SMARTvector inducible plasmid either with non-targeted control (Dharmacon, Catalog#: VSC11531) or G3BP1 specific shRNA (ATTCCGAGACACCAAACGC)(Dharmacon Cat# V3SM11253-234970007). ARKO (knockout) cells were generously provided by Dr. Barbieri. For overexpression, WT, *Pten*^*−/−*^, *Pten*^*−/−*^ *SPOP*^*F133V*^, and ARKO cells were infected with DOX-inducible empty vector (control) and pCW57.1-FLAG G3BP1 (overexpressed) lenti-virus. Cells were selected with puromycin until resistant populations emerged, and then treated with 1 μg/mL DOX for 72 h followed by RFP positive cell sorting. G3BP1 stable knockdown or overexpression was confirmed by immunoblot analysis.

#### Transient models

22Rv1 cells were transfected with either non-targeted control, ON-TARGETplus smart pool or G3BP1 siRNA (Dharmacon Cat# D-001810-10-05, L-012099-00-0005), and siGenome smartpools to target SPOP (M-017919-02-0005), using Lipofectamine RNAiMAX (Thermo Fisher Scientific Cat# 13778150). Twenty-four hours post-transfection, cells were plated onto migration and invasion chambers (corning Cat# 3462 and 354480) and assessed for migratory and invasive cells.

#### Generation of expression construct

Using Phusion High-Fidelity DNA Polymerase and PCR primers, FLAG-G3BP1^Acidic (139^^–^^338)^ and FLAG-G3BP1^PxxP (222^^−^^338)^ were constructed by insertion of the PCR-amplified G3BP1^Acidic (139^^−^^338)^ and FLAG-G3BP1^PxxP (222^^−^^338)^ sequences into the mammalian expression vector pFLAG-CMV. In vitro site-directed mutagenesis was used to obtain the pcDNA3.1-HA-BTB^L186D^, pcDNA3.1-HA-BTB^L190D^, pcDNA3.1-HA-BTB^L193D^, and pcDNA3.1-HA-BTB^I217K^ mutant (BTB^mut^) by consecutive two-PCR amplifications using pcDNA3.1-HA-BTB as the template. The PCR-amplified DNAs coding for mutated BTB were inserted into pcDNA3.1 Hygro (+) to generate the corresponding mammalian expression vectors: pcDNA3.1-BTB^mut^. Lentiviral, doxycycline-inducible expression vectors for G3BP1^WT^ were generated by the restriction enzyme cloning technique using the lentiviral vector pCW57-RFP-P2A-MCS [(Addgene plasmid# 78933), a kind gift from Dr. Adam Karpf, University of Nebraska Medical Center]. Briefly, using Phusion High-Fidelity DNA Polymerase and PCR primers, FLAG-G3BP1^WT^ were constructed by insertion of the PCR-amplified G3BP1^WT^ sequences into the mammalian expression vector pCW57-RFP-P2A-MCS. In vitro site-directed mutagenesis was used to generate G3BP1^Mut^ construct using the Luc-G3BP1 as a template. For all constructs, correct insertion was confirmed by Sanger sequencing. The PCR primer sequences used to generate these constructs are in Supplementary Table 1.

#### Human pathology review

All sections were reviewed by a board-certified genitourinary pathologist with expertize in human prostate cancer (B.D.R). Reviews were performed independently.

### Method details

#### Mass spectrometry

The samples were treated with SDS–PAGE loading buffer supplied with 10 mM DTT for 5 min at 85 °C. The proteins were alkylated by the addition of iodoacetamide to the final concentration of 15 mM. The samples were subjected to SDS–PAGE and the whole lanes were cut out and digested with trypsin in-gel for 2 h. The resulting peptides were extracted, dried, and resuspended in 0.1% formic acid with 5% acetonitrile prior to loading onto a trap EASY-column (Thermo Scientific) coupled to an in-house-made nano HPLC column (20 cm × 75 μm) packed with LUNA C18 media. Analysis was performed on a Velos Pro mass spectrometer (Thermo Scientific) operated in data-dependent mode using 90-min gradients in an EASY-LC system (Proxeon) with 95% water, 5% acetonitrile (ACN), 0.1% formic acid (FA) (solvent A), and 95% ACN, 5% water, 0.1% FA (solvent B) at a flow rate of 220 nl/min. The acquisition cycle consisted of a survey MS scan in the normal mode followed by twelve data-dependent MS/MS scans acquired in the rapid mode. Dynamic exclusion was used with the following parameters: exclusion size 500, repeat count 1, repeat duration 10 s, and exclusion time 45 s. Target value was set at 10^4^ for tandem MS scan. The precursor isolation window was set at 2 *m*/*z*. The complete analysis comprised two independent biological replicates.

### Fluorescence activated cell sorting

CFSE dye dilution and CellTrace Violet cell proliferation assays: 22Rv1shCtrl and 22Rv1shG3BP1 (500,000 cells/ml) were stained with CFSE or CellTrace Violet according to the manufacturer’s instructions for 48 h. Dilution of CFSE or CellTrace Violet fluorescence as an indicator of cell division was assessed using fluorescence activated cell sorting analysis. Data were analyzed using FCS express version 6 software to delineate the percentage of cells that had undergone increasing number of divisions to determine the proliferation index.

Cell cycle analysis: cell cycle analysis was performed by flow cytometry using standard procedures as described previously^57^. Briefly, 22Rv1shCtrl and 22Rv1shG3BP1 (2 × 10^6^/ml) cells were fixed with 70% ethanol followed by treatment with RNase A for 10 min and then stained with DAPI (DAPI 1 μg/mL, Triton X100 0.1%) for 30 min in the dark. Analysis was performed on a flow cytometer Fortessa, and data analysis was conducted using FCS express version 6 software. All samples were analyzed in triplicate, and the data presented are the average of three independent experiments.

Annexin-V-PI staining: Occurrence of apoptosis was measured using the Annexin-V-FITC Apoptosis Detection Kit according to the manufacturer’s protocol. Briefly, 22Rv1shCtrl and 22Rv1shG3BP1cells were suspended with binding buffer and incubated with Annexin-V-FITC and propidium iodide in binding buffer for 10 min at room temperature. Analysis was performed on a flow cytometer Fortessa, and data analysis was conducted using FCS express version 6 software. All samples were analyzed in triplicate, and the data presented are the average of the three independent experiments.

#### Co-immunoprecipitation

Coimmunoprecipitation was conducted according to a standard protocol described previously^8^ with minor modification. Briefly, HEK 293T cells were transfected with HA-SPOP (full length or its deletion mutants HA-SPOP^MATH^ or HA-SPOP^BTB^) and Myc-G3BP1 or HA-SPOP-full length and FLAG-G3BP1 [Full length and different G3BP1 deletion mutants FLAG-G3BP1^(1^^−^^138)^, FLAG-G3BP1^(139^^–^^466)^, FLAG-G3BP1^(222^^–^^466)^, FLAG-G3BP1^(338^^–^^466)^, FLAG-G3BP1^(139^^–^^338)^, and FLAG-G3BP1^(222^^–^^338)^ respectively]. Two days after transfection, cell lysates were prepared by lysing cells in buffer containing 50 mM Tris (pH 7.5), 120 mM NaCl, 1% NP-40, 5 mM EDTA supplemented with protease inhibitor cocktail, sodium fluoride, and activated sodium vanadate. In all, 500 μg of proteins were incubated with respective antibodies HA, MYC, or FLAG overnight at 4 °C. The next day, lysates were incubated with Protein G sepharose beads for 2 h and washed three times using lysis buffer. Input, IgG, and IP were next resolved through SDS–PAGE, transferred onto a PVDF membrane and incubated overnight at 4 °C with primary antibodies. Odyssey® immunoblot developer was used to develop immunoblots. Briefly, after transfer the membranes were first blocked with Odyssey® Blocking Buffer (PBS) (LI-COR) for 1 h, then washed with wash buffer (2 M Tris pH 7.5, 0.5 M EDTA pH 8.0, 5 M NaCl, Tween 20, and water) three times for 5 mins each. Then secondary antibody (mouse or rabbit IR-Dye either 800CW,or 680RD) was added at 1:20,000 for 1 h in Odyssey® Blocking Buffer (PBS). Afterward, the membrane was washed with wash buffer three times for 5 mins each. Finally, the membrane was washed with PBS and scanned on an Odyssey^®^ CLx. For antibody use and details please see Supplementary Table 2.

#### Immunoblot assays

Extraction of protein from the cell: For protein extraction, cell pellets were collected in cold PBS, and RIPA buffer (150 mM NaCl, 1% NP-40, 0.5% sodium deoxycholate, 0.1% SDS and 50 mM Tris pH 8.0) was used for extraction.

Extraction of protein from the xenografted tumor tissue: Fresh tissue was homogenized, and proteins were extracted with RIPA buffer.

Extraction of nuclear protein from the cell: Nuclear extraction of protein was carried out using the Active Motif kit according to the manufacture’s protocol.

For immunoblot analysis, protein lysates were separated by SDS–PAGE and transferred onto nitrocellulose membranes. Immunoblotting was conducted using protein-specific antibodies. Odyssey® immunoblot developer was used to develop immunoblots as described above. For antibody use and details see Supplementary Table 2.

For MG132 treatment, cells were treated with 10 µM MG132 for 6 h and harvested using RIPA lysis buffer followed by immunoblot analysis.

#### Analysis of protein stability

Analysis of protein stability by cycloheximide chase assay was performed using standard procedures as described previously^58^. Briefly, 22Rv1sgCtrl and 22Rv1sgG3BP1, C4-2B non-targeted and C4-2B SPOP KO cells were incubated with 100 µM of cycloheximide for various times as indicated in the figures. Cell lysates were prepared and the expression of TRIM24 and G3BP1 were analyzed by immunoblot.

#### Drug sensitivity assay

Cell viability and drug sensitivity were measured using a CellTiter-Glo® Cell Viability Assay Kit (Promega) according to the manufacturer’s protocol. Briefly, cells were cultured in sextuplicate in 96-well plates (∼250 cells per well) and incubated for 24 h to allow cell attachment on the surface of the wells in charcoal-stripped serum. Then, cells were exposed to different concentration of drugs. The effects on cell viability were tested when drugs present in the culture medium. On day six, cell viability was assessed by adding 100 μL per well of cell titer glow (Promega) followed by a 10 min incubation at 37 °C and measured by the amount of luminescence using a 96-well plate luminometer (GlowMax; Promega). Background was subtracted using the medium-only control wells.

#### In situ proximity ligation assay

Parental 22Rv1 cells were transfected with FLAG-G3BP1, FLAG-G3BP1^NTF2^, FLAG-G3BP1^RRM^, or FLAG-G3BP1^PxxP^. Half of the transfected cells were used for immunofluorescence analysis and half of the transfected cells were used for the proximity ligation assay (PLA) using Duolink II Kit (Millipore-Sigma). Immunofluorescence microscopic analysis was performed as previously described^59^. Briefly, after fixation, permeabilization, and blocking, cells were incubated with primary antibodies against FLAG-M2 and SPOP overnight at 4 °C at 1:1000 and 1:200 dilutions in 1% goat serum containing PBS. The next day, after three washes with PBS, anti-rabbit IgG Alexa Fluor 488, and anti-mouse IgG Alexa Fluor 594 were added at 1:500 dilutions in 1% goat serum containing PBS and incubated at room temperature for 1 h. After washes in PBS and in water, nuclei were visualized with DAPI by mounting with vectashield Hard Set mounting medium (Vector Laboratories). For the PLA assay, fixed and permeabilized cells were incubated with antibodies against FLAG-M2 (Millipore-Sigma) and SPOP (rabbit polyclonal antibody, Proteintech cat#16750-1-AP. Interactions were revealed using secondary antibodies coupled to specific PLA DNA probes that hybridized and were enzymatically joined when located in close proximity. After rolling circle amplification, each interaction generated a fluorescent spot that was analyzed by confocal microscopy (LSM 880 Carl Zeiss). For enumeration, more than ten fields were randomly selected and a total of 130-450 cells were visually scored for each sample.

#### Migration and invasion assay

The siRNA oligonucleotides were transfected with RNAiMAX (Invitrogen) at a final concentration of 50–100 pmol. 24 h after transfection, cells were plated on the migration and invasion chamber. In total, 15 × 10^4^ (for migration) or 7.5 × 10^4^ (for invasion) transfected 22Rv1 cells with control, SPOP siRNA or G3BP1 siRNA or SPOP siRNA and G3BP1 siRNA were suspended in 0.5 ml of RPMI-1640 medium containing 1% FBS and placed into the top chamber of without and with Matrigel-coated 8 μm Trans-well inserts (BD Falcon). The bottom wells contained RPMI supplemented with 10% FBS. After 48 h, the filters were fixed and stained with 0.2% crystal violet for 5 min, and cells on the upper surface of the filters were removed with a cotton swab. Migrated and invaded cells were quantified by counting the numbers of cells that penetrated the membrane in four microscopic fields (viewed at ×10 magnification) per filter. All experiments were performed in triplicate; results are the average of three independent experiments.

#### In vivo tumorigenesis study

All procedures involving mice were approved by the Institutional Animal Care and Use Committee (approval number 2017-0008) at Weill Cornell Medicine and complied with all relevant ethical regulations. Mice were maintained in micro-isolators in the USDA (U.S. Department of Agriculture)/AALAC (American Association of Laboratory Animal Science) accredited facility at the Weill Cornell Medicine on a 12 h light, 12 h dark cycle at 64 °F. The mice were allowed food and acidified water ad libitum. 3–4-week-old male NU/J mice (Jackson Laboratories, Bar Harbor, Maine) were used. To evaluate the role of G3BP1 in tumor formation, 2 million 22Rv1 cells stably expressing non-targeted and sgG3BP1were harvested using 0.05% trypsin-EDTA (Gibco BRL) washed twice and suspended in HBSS (Gibco BRL), without serum, immediately prior to mixing with Matrigel (BD Biosciences). The cell concentrations were adjusted to deliver the desired number of cells in a total volume of 0.05 ml with a 1:1 dilution of Matrigel. In all, 2 × 10^6^ cells were injected subcutaneously into the dorsal flank of the mice (*n* = 12). Tumor size was measured twice a week, tumor volumes were estimated using the formula (*π*/6) (*L* × *W*2), where *L* = the length of tumor and *W* = the width, and tumor weight was measured after the mice were euthanized. After the endpoint was reached, the mice were euthanized, and tumors were excised for subsequent analysis. The maximal tumor size/burden permitted is 2 cm^3^ by Institutional Animal Care and Use Committee. The maximal tumor size/burden was not exceeded.

#### Organoid formation assay

Organoid formation assays were performed as previously described^12,47^. Briefly, cells were plated in Matrigel (BD 356231) and covered with media containing 5 ng/mL EGF. Cells were cultured with 1 μg/mL Doxycycline to induce shRNA or FLAG-G3BP1 expression. For the “organoid formation assay” 10 single cells were plated per well (total of 24 wells) on day 1. The number of formed organoids was counted on days 7 and 14 post-plating. The size of organoids was measured on day 14 with ImageJ2 (NIH, US).

#### Quantitative real-time PCR

RNA extraction of cell pellets was done using the RNeasy Plus Kit (Qiagen) following the manufacturer’s protocol. cDNA synthesis was performed using a qScript cDNA synthesis Kit (Quanta Bioscience) following the manufacturer’s protocol. Quantitative PCR was done on the Roche Light Cycler 480 using SYBR Green 1 Master (Applied Biosystems). Sequences of the primers used are listed in Supplementary Table 1.

#### Tissue staining

For deparaffinization, tissues were baked in an oven for 10–20 min at 60 °C, then treated with xylene twice for 10 min each. Rehydration was carried out in, successively, 100% ethanol, 95% ethanol, 70% ethanol, 50% ethanol, and distilled water for 2 min each. For antigen retrieval, slides were immersed in boiling citrate buffer, pH 6.0 for 30–40 min, immediately followed by cooling the slides under running tap water for 20 mins, then washing twice (5 min each) with wash buffer (dako-S3006). Permeabilization was accomplished by incubating each section in 0.5% wash buffer for 15 min followed by two washes with wash buffer (5 min each). For serum blocking, a 60 min incubation in 10% goat serum in wash buffer was used. Primary antibodies addition was performed in an overnight incubation of the section in antibody diluted in 5% goat serum in wash buffer, followed by washing three times in wash buffer (5 min each). Secondary antibodies were added in a 60 min incubation with fluorescent conjugated secondary antibodies (Alexa fluor 488 and 594) at 1:500 dilution in a solution of 5% goat serum and 1% BSA wash buffer at room temperature for 1 h. This was followed by three wash steps with wash buffer (5 min each). Sections were mounted with fluorescent mounting medium with DAPI (vectashield with DAPI) and covered with coverslips. DNA fragmentation was detected with an In-Situ Cell Death Detection Kit TMR Red (Roche Diagnostics) according to the manufacturer’s protocol. For antibody use and details see Supplementary Table 2.

#### In vivo ubiquitylation assay

HEK 293T cells were transiently co-transfected with various plasmid constructs as mentioned in the respective figures. Forty-eight hours post-transfection, cells were treated with 10 µM MG132 for 5 h and harvested under denaturing conditions, as described previously^10^. His-Ub–conjugated cellular proteins were purified by Ni-NTA agarose resin. Ubiquitinated proteins were detected using SDS–PAGE and immunoblotting with the respective antibodies. Immunoblot analysis was conducted to detect respective proteins as mentioned in the figures.

#### In vitro ubiquitylation assay

Each reaction was performed with 100 nM DEK (OriGene) in 20 μL of ubiquitylation buffer (50 mM Tris-HCl, 200 mM NaCl, 1 mM DTT, 10 mM MgCl_2_) containing 4 mM ATP, 5 nM SPOP (Novus Biologicals), 10 nM Cul3/Rbx1 (Ubiquigent), 500 nM UbcH5b, 100 nM UbE1 (Boston Biochem, Inc.), and 25 μM ubiquitin (Boston Biochem, Inc.), without or with various concentration (0.25, 0.5, and 1 nM) of G3BP1 (Prospec). The reactions were incubated at 37 °C for 1 h and stopped by the addition of SDS sample buffer. Samples were separated by 10% SDS–PAGE and analyzed for the respective antibodies mentioned in the figure.

#### Binding assay

In total, 4 µg His-SPOP and different amount (0.125, 0.25, or 0.75 µg) of His G3BP1 proteins were mixed in 1 ml pulldown buffer at 4 °C for 60 min. Then add 0.56 µg Myc-DEK protein into the tube. For pre-clearing, the protein mixtures were incubated with 30 µl protein G agarose (GE, 17-0618-01) at 4 °C for 60 min. After centrifugation, the supernatant was transfer to new tube and add anti-SPOP (Proteintech cat#16750-1-AP) at 4 °C for overnight. Then add 40 µl protein G agarose to each sample and incubated on the rotated shake at 4 °C for 2 h. The protein G agarose was washed three times with a buffer containing 50 mM Tris (pH 7.5), 200 mM NaCl and proteinase inhibitor cocktails. After washing, 30 µl of 2x SDS loading buffer was added and boiled at 95 °C for 5 min. For immunoblot analysis, equal amounts of protein samples were loaded into a 9% SDS–PAGE gels and probed with anti-SPOP, anti-G3BP1, and anti-DEK antibodies.

#### Caspase 3/7 activity assay

Caspase-3 and caspase-7 activity was determined after 48 h induction of shRNA using the Caspase–Glo 3/7 assay kit (catalog number G8091) following the manufacturer’s instructions. Briefly, Caspase–Glo 3/7 reagent was added to each well in a 1:1 ratio and incubated with gentle shaking for 30 min at room temperature before measuring luminescence using GlowMax luminometer.

#### RNase treatment and immunoprecipitation assay

RNase treatment and immunoprecipitation assay were performed using standard procedures as described previously^60^. Full-length G3BP1 and G3BP1^RRM^ were transfected in 22Rv1 cells. 48 h after transfection, cells were lysed with NP40 lysis buffer. Lysates of G3BP1 and G3BP1^RRM^ transfected cells were treated without or with 100 U RNase ONE (Promega #M4261) for 1 h at 37 °C. Efficient RNase ONE treatment was confirmed by running samples into Agilent bioanalyzer. RNase ONE treated and untreated lysates were immunoprecipitated and immunoblotted with a specific antibody as described above.

#### Luciferase reporter assay

PCa cells were transfected with 1.0 µg full-length human G3BP1 promoter construct ligated to secreted and robust Gaussia Luciferase (GLuc) as the reporter (GeneCopoeia cat#HPRM46317-PG04) using X-tremeGENE™ HP DNA transfection reagent following manufacturer’s protocol. The G3BP1 promoter construct contains a 1.6 kb insert, corresponding to the 5′-flanking promoter sequence located ~1.6 kb upstream and up to 200 bp downstream of the transcription start site (TSS) of a specific human G3BP1gene. Using site-directed mutagenesis kit, we generated a luciferase reporter mutant construct driven by 1.6 kb region containing (AGAA**C**TgctC**A**CTCG has been changed to AGAA**T**TgctC**G**CTCG) the mutation in ARE site (ARE^Mut^). The dual-reporter system uses GLuc as the promoter reporter and SEAP (secreted Alkaline Phosphatase) as the internal control for signal normalization. Six hours after transfection, cells were rinsed with PBS and fresh medium was added for recovery. Cells were treated with enzalutamide for 48 h and DHT for 24 h respectively. Reporter activities were then measured using the Secrete-Pair Dual Luminescence Assay Kit (GeneCopoeia cat# LF032).

#### Stress granule formation assay

Stress granule formation assay was performed using standard procedures as described previously^61^. Stress was induced by treating LNCaP cells with 0.5 mM sodium arsenite for 30 min followed by immunofluorescence detection of G3BP1 and SPOP as described above.

### Quantification and statistical analysis

#### MS data analysis

MS spectrum files were transformed into MGF format by MSConvert software v1.0 and interrogated by a MASCOT 2.4 search engine using human UniProt database version 15 concatenated with reverse sequences for estimation of false discovery rate (FDR) and with a list of common contaminants. The search parameters were as follows: full tryptic search, 2 allowed missed cleavages, peptide charges +2 and +3 only, MS tolerance 1 Da, and MS/MS tolerance 0.5 Da. The only permanent post-translational modification was cysteine carbamidomethylation. Variable post-translational modifications were protein N-terminal acetylation, Met oxidation and N-terminal Glutamine to pyro-Glutamate conversion. The remaining analysis was performed as in^62^. To summarize, the minimal ion score threshold was chosen such that a peptide false discovery rate (FDR) below 1% was achieved. The peptide FDR was calculated as: 2 × (decoy_hits)/(target + decoy hits). Spectral counts for all detected proteins were assembled using a Python script written in-house. The adjustment of spectral counts was done by the same script as in^62^. The mass spectrometry proteomics data have been deposited to the ProteomeXchange Consortium via the PRIDE^63^ partner repository with the dataset identifier PXD029120.

#### Analysis of human TMAs (protein)

In order to analyze G3BP1 protein expression in a larger number of different prostate cancer patient samples, Weill Cornell Medicine constructed tissue microarrays (TMAs) containing 153 formalin-fixed, paraffin-embedded, prostate cancer tissues. The specimens were collected between 2001 and 2011 in Weill Cornell Medicine, NY, USA. Hematoxylin- and eosin-stained slides of all specimens were re-evaluated by one experienced pathologist (B.D.R.) to identify representative areas. Patients had an average age of 58 years (range: 37–76). The local scientific ethics committees and Weill Cornell Medicine approved the protocol (Protocol # 1007011157) and the cohort. This cohort was used to correlate G3BP1 protein expression determined by IHC with TRIM24 and AR.

#### Global transcriptome analysis

All analysis of human prostate cancer data was conducted using previously published datasets of The Cancer Genome Atlas (TCGA) cohort^16^, Taylor cohort^64^, and PCF/SU2C^15^, which can be explored in the cBioPortal for Cancer Genomics (http://www.cbioportal.org). The AR output score of different cohorts were calculated by following a strategy similar to that of the TCGA study^16^. Specifically, the AR output score was derived from the mRNA expression of 20 genes that were experimentally validated AR transcriptional targets from the LNCaP cell line^39^. Here the Z-score for the expression of each gene in each sample was calculated and the AR score for each sample was then computed as the sum of the *Z*-scores of 20 AR signaling genes. Statistics: For comparison of pooled data between two different groups, unpaired t tests were used to determine significance. For Kaplan–Meier analysis of MET-free survival rates, expression profiles of retrospective (*n* = 1626) cohorts were derived from the Decipher Genomics Resource Information Database (GRID) registry (ClinicalTrials.gov identifier: NCT02609269). The retrospective GRID cohort was pooled from seven published microarray studies: Cleveland Clinic (CCF)^65^, Erasmus MC^66^, Johns Hopkins (JHMI)^67^, Memorial Sloan Kettering (MSKCC)^68^, Mayo Clinic (Mayo I and Mayo II)^69^, and Thomas Jefferson University (TJU)^70^. Associated accession numbers are: GSE79957, GSE72291, GSE62667, GSE62116, GSE46691, GSE41408, and GSE21032^71^.

#### RNA-seq analysis

22Rv1 control, 22Rv1 siSPOP, 22Rv1 G3BP1 KO, and 22Rv1 G3BP1 KO plus siSPOP (3 replicates) were prepared for RNA sequencing using TruSeq RNA Library Preparation Kit v2. Each sample was sequenced with the HiSeq 2500 to generate 51 bp paired-end reads. Reads (FASTQ files) were mapped to the human reference genome sequence (hg19/GRCh37 http://hgdownload.cse.ucsc.edu/downloads.html) using STAR v2.4.0j^72^, and the resulting BAM files were subsequently converted into mapped-read format (MRF) using RSEQtools v0.6^73^. The read count of each gene was calculated via HTSeq^74^ using GENCODE as the reference gene–annotation set. Quantification of gene expression was performed using RSEQtools with GENCODE as the reference gene–annotation set (http://www.gencodegenes.org/human/release_19.html). Expression levels (RPKM) were estimated by counting all nucleotides mapped to each gene and were normalized by the total number of mapped nucleotides (per million) and the gene length (per kb). Differential expression analyses were performed using DESeq2 (v1.20.0)^75^ based on the gene read count data. Multiple-hypothesis testing was considered by using the Benjamini-Hochberg (BH; FDR) correction. Heatmap and hierarchical clustering were performed via using correlation distance and Ward’s method. GSEA^76^ was performed using the JAVA program and run in pre-ranked mode to identify enriched signatures. We used the gene sets in the Molecular Signature Database (MSigDB)^76^. The GSEA plot, normalized enrichment score and *p*-values were derived from GSEA output for each MSigDB hallmark signature. The AR output scores of our samples were calculated by following a strategy similar to that of the TCGA study^16^. Specifically, the AR output score was derived from the mRNA expression of 20 genes that were experimentally validated AR transcriptional targets from the LNCaP cell line^39^. Here the *Z*-score for the expression of each gene in each sample was calculated, and the AR score for each sample was then computed as the sum of the *Z*-scores of 20 AR signaling genes. By following a similar strategy^12^, we developed the G3BP1 overexpression transcriptional signature, which includes 498 overexpressed genes from samples with G3BP1 that was overexpressed compared with wild-type samples from TCGA prostate cancer RNA-seq data. Specifically, we identified significantly differentially expressed genes by comparing G3BP1 overexpression and wild-type cases as determined from genomic analyses among TCGA samples with ETS family gene fusions (ERG, ETV1, ETV4, and FLI1), using DESeq2 (v1.20.0)^75^ and controlled for false discovery using Benjamini–Hochberg adjustment (FDR ≤ 0.05).

#### Ki67 quantification

Three independent, representative images were taken at ×20 magnification for each mouse prostate. The percentage of Ki67 positive epithelial cells was calculated for each image and the mean and standard error of all images for each genotype was determined. A Student’s two-sided *t*-test was used to determine statistical differences between two genotypes.

#### Tumor-xenograft volume quantification

Volume (mm^3^) for each xenograft was calculated and plotted. The difference in the mean volume between xenograft lines was determined using a two-sided Student’s *t*-test.

#### PLA quantification

A minimum of 200 cells per condition were quantified in a single plane for detectable foci. The number of foci per cell per condition is reported. Differences in the mean numbers of foci between conditions were determined using a 2-sided Student’s t-test.

### Reporting summary

Further information on research design is available in the Nature Research Reporting Summary linked to this article.

## Supplementary information

Supplementary Information

Reporting Summary

## Data Availability

The RNA-seq data generated in this study have been deposited in the NCBI Gene Expression Omnibus (GEO) database under the accession code GSE 138612. Original/source data for Fig. 3a of this study is available in Cell 163, 1011–1025 (2015)^16^. Original/source data for Fig. 3b of this study is available in Decipher; GenomeDx Biosciences, Vancouver, BC, Canada^37^. Original/source data for Fig. 4g of this study is available in refs. ^15,16,64^. All data supporting the findings of this study are available within the article and its Supplementary Information files. A reporting summary for this article is available as a Supplementary Information file. Source data are provided with this paper. The data that support the finding in Supplementary Fig. 1A of this study is deposited in proteomeXchange accession no. PXD029120. Source data are provided with this paper.

## Source data

Source Data
