# Supplementary material for: G3BP1 inhibits Cul3SPOP to amplify AR signaling and promote prostate cancer
Source: Nat Commun. 2021 Nov 18;12:6662. doi: 10.1038/s41467-021-27024-x (PMC8602290; doi:10.1038/s41467-021-27024-x)

File Name: Supplementary Information
Description: Supplementary Figures 1 to 7 with figure legends and Supplementary Tables 1 and 2

File Name: Source Data
Description: Quantitative data of the figures, uncropped blots and FACS plots for gating strategy

Supplementary Table 1: Detailed primer list

**Oligonucleotides**		
Primers used to generate constructs		
Forward-Ac-PxxP	This Paper	TCTGCG GCC GC TGGGTTTGTCACTGAGCCTCA
Forward-PxxP	This Paper	TCTGCG GCC GC T GCC CCT GAG GAT GCT CAG
Reverse-Ac-PxxP	This Paper	GCGGATCC TTA GTCAGGGTGTCTCACCATTC
Forward primer for generating inducible mouse G3BP1 overexpression construct	This Paper	GCCTGCAGGCCACCATGGACTACAAGGACGACGAT GACAAAGATGTTATGGAGAAGCCTAG
Reverse primer for generating inducible mouse G3BP1 overexpression construct	This Paper	GCGGATCCTTACTGCCT TGG AGTTGTAA
Mutation primer SPOP L186D	This Paper	p-CCTGAGTGCCGGGACGCAGATGAGTTAGGAGGA
Mutation primer SPOP I217K	This Paper	p-CAGGCTCACAAGGCTAAATTAGCAGCTCGTTCTCCG
Mutation primer SPOP L190D	This Paper	p-CGGCTGGCAGATGAGGATGGAGGAGACTGGG AGAATTCCCGG
Mutation primer Luc-G3BP1^{MUT}	This Paper	p-GTCACATGCCTGGAACGAGAATAAACCAGGGCGTG
qRT-PCR primers		
G3BP1 Human Forward	This Paper	GTCCAGAGTCTAAGCCTGAATC
G3BP1 Human Reverse	This Paper	ATGTCACCTTGCTCACCAG
KLK3 Human Forward	Fong et al., 2018	GTTGTCTTCCTCACCCTGTCC
KLK3 Human Reverse	Fong et al., 2018	GGTTGGGAATGCTTCTCG
TMPRSS2 Human Forward	Fong et al., 2018	GGACAGTGTGCACCTCAAAGAC
TMPRSS2 Human Reverse	Fong et al., 2018	TCCCACGAGGAAGGTCCC
SPOP Human Forward	This Paper	TGACCACCAGGTAGACAGCG
SPOP Human Reverse	This Paper	CCCGTTTCCCCCAAGTTA
KLK2 Human Forward	Fong et al., 2018	GAA CCA GAG GAG TTC TTG CG
KLK2 Human Reverse	Fong et al., 2018	CCC AGA ATC ACC CCC ACA A
β-actin Human Forward	Groner et al., 2016	AGAGCTACGAGCTGCCTGAC
β-actin Human Reverse	Groner et al., 2016	AGCACTGTGTTGGCGTACAG
G3BP1 Mouse Forward	This Paper	TGGTGTGGCTAACTGAATA
G3BP1 Mouse Reverse	This Paper	TAGACATTGACAGGCTACAT
FKBP5 Mouse Forward	Blattner et al., 2017	TGA GGG CAC CAG TAA CAA TGG
FKBP5 Mouse Reverse	Blattner et al., 2017	CAA CAT CCC TTT GTA GTG GAC AT
Igfbp3 Mouse Forward	Chua et al., 2014	CCA GGA AAC ATC AGT GAG TCC
Igfbp3 Mouse Reverse	Chua et al., 2014	GGA TGG AAC TTG GAA TCG GTC A
GAPDH Mouse Forward	Mukhopadhyay et al., 2016	CCTGGAGAAACCTGCCAAGTATG
GAPDH Mouse Reverse	Mukhopadhyay et al., 2016	GAGTGGGAGTTGCTGTTGAAGTC
G3BP2 Mouse Forward	This Paper	AGA CTG AAG AGC TGA AAC CAC
G3BP2 Mouse Reverse	This Paper	TTT ACT GGT CAC TGA AGC CC

Supplementary Table 2: Detailed antibody usage

Name	Company	Application	Dilution	Cat #
HA	Biolegend	Immunoblot	1:2000	901502
Myc	Cell Signaling	Immunoblot	1:1000	2278S
FLAG	Sigma	Immunoblot/IF	1:2000 /1:1000	F1804-1
His	Cell Signaling	Immunoblot	1:2000	2365S
Cul3	Cell Signaling	Immunoblot	1:1000	2759
G3BP1	Protein tech	Immunoblot	1:1000	I3057-2-AP
G3BP1	Abcam	IF/PLA	1:1000/1:4000	56574
SPOP	Proteintech	Immunoblot/IF/PLA	1:1000/1:200/1:2000	16750-1-AP
SRC3	Cell Signaling	Immunoblot	1:500	2126S
AR	Abcam	Immunoblot	1:1000	ab133273
AR	Santa Cruz	Immunoblot	1:500	sc-816
TRIM24	Santa Cruz	Immunoblot	1:500	sc-271266
α-tubulin	Protein Tech	Immunoblot	1:5000	6603I-I-ig
Laminin B	Santa Cruz	Immunoblot	1:500	sc-56143
β-actin	Protein Tech	Immunoblot	1:5000	20536-I-AP
G3BP2	Abcam	Immunoblot	1:1000	ab86135
Mouse IgG	BioLegend	Immunoprecipitation	1μg/reaction	400102
Rabbit IgG	Abcam	Immunoprecipitation	1μg/reaction	ab37415
Ki67	Bethyl	IHC/IF	1:1000	A700-021
Ecadherin	BD Bios	IF	1:2000	610405
Cytokeratin 5	Biolegend	IHC	1:5000	905501
Cytokeratin 8	Progen	IHC	1:2000	61038S
RFP	Abcam	Immunoblot	1:1000	ab62341
Cleaved Parp	Abcam	Immunoblot	1:1000	ab32064
PSA (KLK3)	Sigma-Aldrich	Immunoblot	1:1000	SAB1410703
TMPRSS2	Abcam	Immunoblot	1:1000	ab92323
FKBP5	Abcam	Immunoblot	1:1000	ab126715
Secondary antibody Mouse 800CW	LI-COR	Immunoblot	1:20,000	925-32210
Secondary antibody Mouse 680RD	LI-COR	Immunoblot	1:20,000	926-68070
Secondary antibody Rabbit 800CW	LI-COR	Immunoblot	1:20,000	926-32211
Secondary antibody Rabbit 680RD	LI-COR	Immunoblot	1:20,000	926-68071
Secondary antibody Alexa fluor 488 Mouse	Thermo Fisher Scientific	Immunofluorescence	1:500	A32723
Secondary antibody Alexa fluor 594 Mouse	Thermo Fisher Scientific	Immunofluorescence	1:500	A32742
Secondary antibody Alexa fluor 488 Rabbit	Thermo Fisher Scientific	Immunofluorescence	1:500	A32731

Secondary antibody Alexa fluor 594 Rabbit	Thermo Fisher Scientific	Immunofluorescence	1:500	A32740

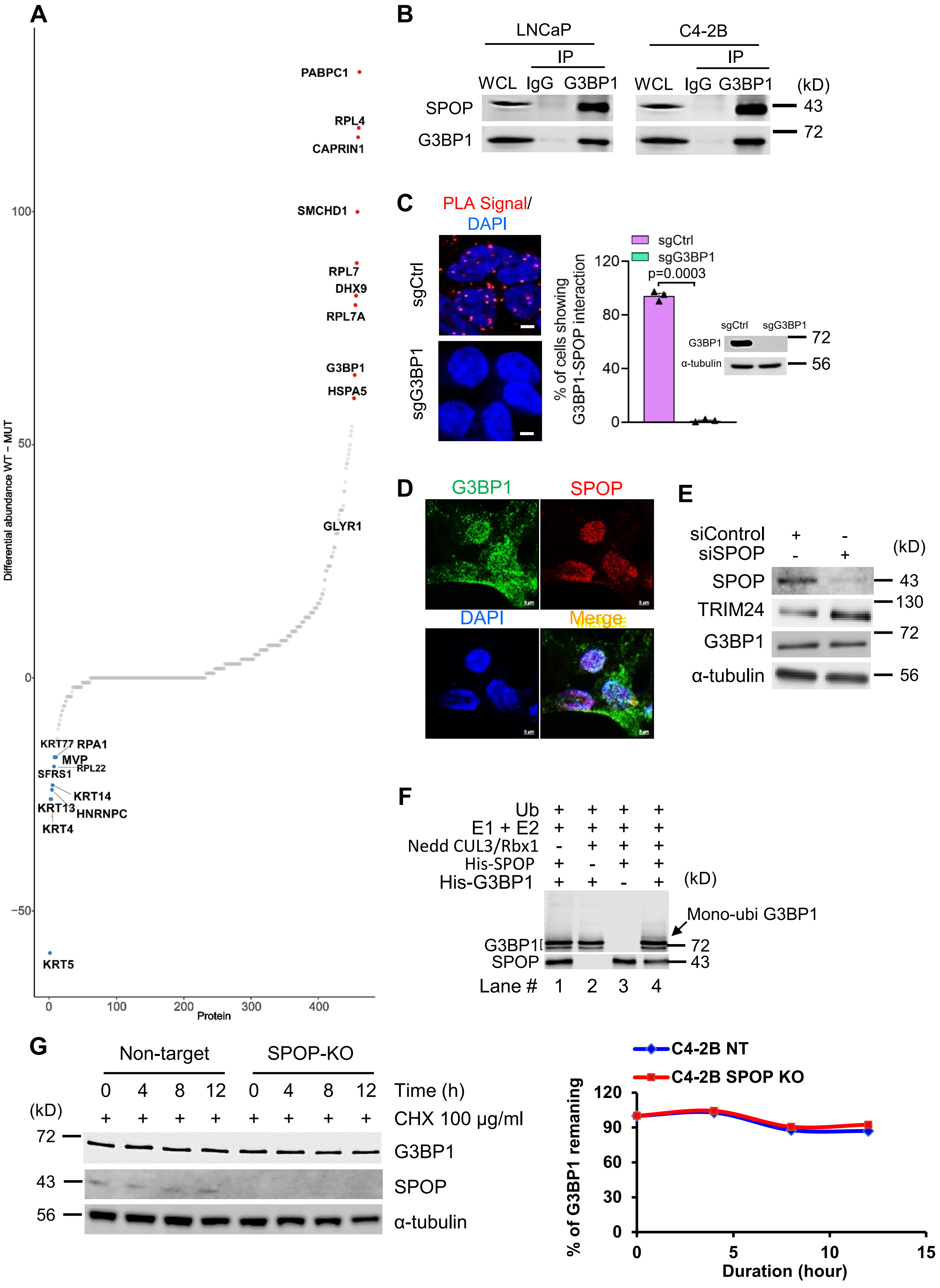

Supplementary Fig. 1A-1G

Supplementary Fig. 1A to 1G. G3BP1 co-localizes and interacts with SPOP to stabilize SPOP substrates.

(A) Scatter plot of differentially abundant proteins in SPOP wild type versus SPOP mutant human prostate cells determined by label-free mass spectrometry (MS/MS). Proteins are plotted on x axis, and differential abundance between SPOP wild type and mutant are plotted on y axis. (**B**) LNCaP and C4-2B cell lysates were subjected to immunoprecipitation (IP) with IgG and anti-G3BP1 antibody and immunoblotted with anti-SPOP and anti-G3BP1 antibodies. n=3 (**C**) Proximity ligation assay (PLA) for the endogenous G3BP1 and SPOP in 22RV1 cells. Each red dot represents an interaction (scale bar, 10 μm). A graph showing percentage of cells with G3BP1 and SPOP interaction in the nucleus n=3 biologically independent experiments. Error bars, ± S.E.M. Cell lysates of LNCaP-sgCtrl and LNCaP-sgG3BP1 cell lines were run and blotted for the indicated proteins. α-tubulin was used as a loading control (inset) n=10. **(D)** Representative immunofluorescence images of 22RV1 cells stained with G3BP1 (green) and SPOP (red). Scale bar, 5 μm. **(E)** Immunoblot analysis of WCL derived from 22RV1-NT (non-targeted), 22RV1-siSPOP cells for the indicated proteins. α-tubulin was used as loading control. n=3 **(F)** G3BP1 mono-ubiquitination (Ub) was reconstituted *in vitro* using affinity-purified recombinant proteins as indicated. Purified proteins and mono-ubiquitinated G3BP1 were subjected to immunoblotting with the indicated antibodies. All protein concentrations were in nM except Ub, which was in μM. n=3 **(G)** Cycloheximide-chase analysis to determine the half-life of endogenous G3BP1 in C4-2B-sgCtrl and C4-2B-sgSPOP cells. The percentage of G3BP1 remaining was graphed at the time points indicated. n=3. Source data are provided at the end of supplementary information file.

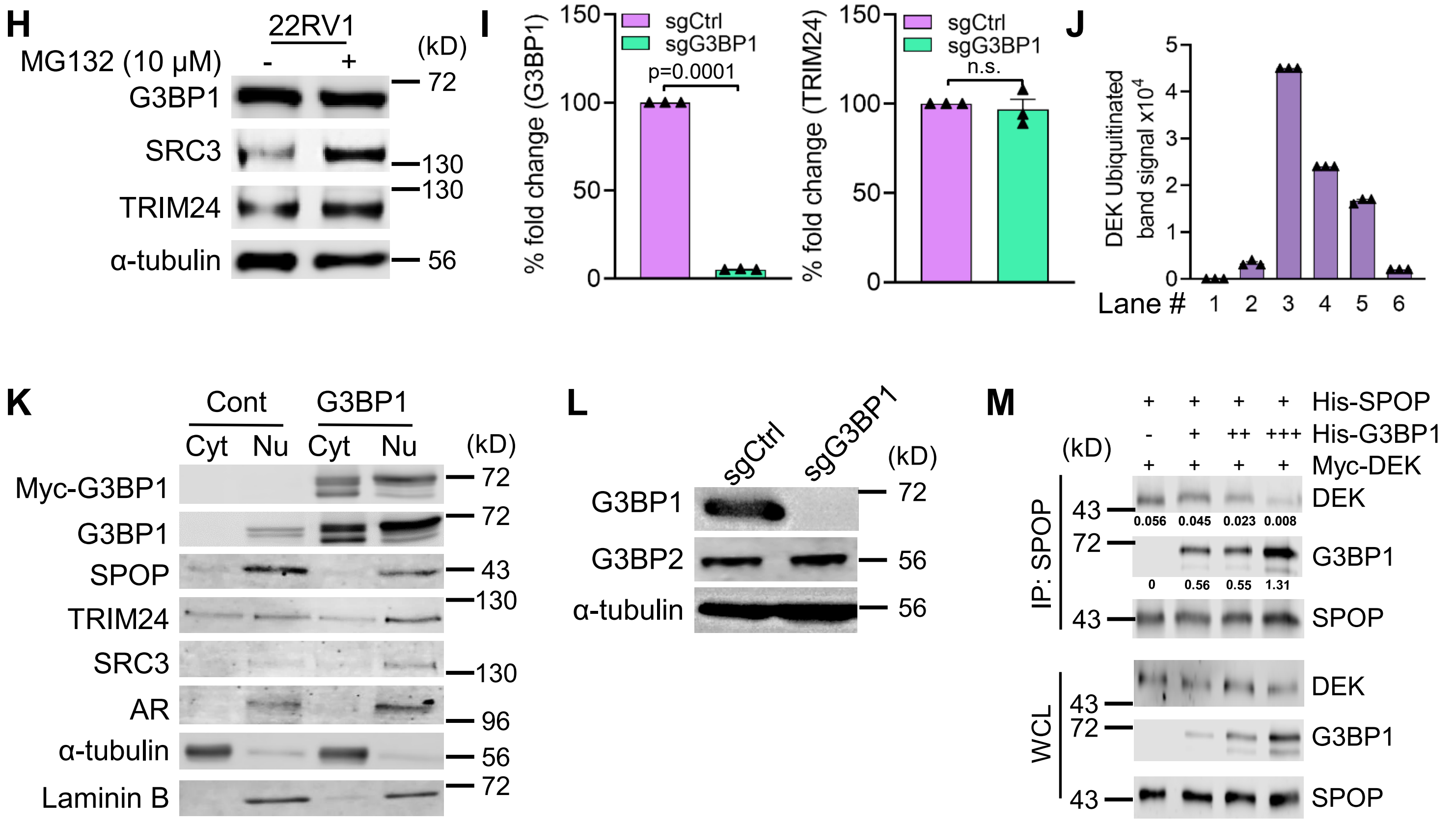

Supplementary Fig. 1H to 1M

Supplementary Fig. 1H to 1M. G3BP1 function as a competitive inhibitor of SPOP E3 ubiquitin ligase.

(H) Immunoblot analyses of WCL from 22RV1 cells treated with 10 μM MG132 for 8 h. n=3 biologically independent experiments. **(I)** RT-qPCR for G3BP1 and TRIM24 transcript in sgCtrl and sgG3BP1 22RV1 cells. n=3 biologically independent experiments. Error bars, ± S.E.M. **(J)** Quantification of DEK ubiquitinated band signal of Figure 1C. n=3 biologically independent experiments. Error bars, ± S.E.M. **(K)** Immunoblot analysis of nuclear and cytoplasmic fractions of LNCaP cells transfected with pcDNA3.1 and Myc-G3BP1 for the indicated proteins. n=3 **(L)** Immunoblot analysis of 22RV1-sgCtrl and 22RV1-sgG3BP1 cells for the indicated proteins. α-tubulin was used as a loading control. n=3 **(M)** Immunoprecipitation and immunoblotting of affinity-purified recombinant proteins as indicated. Increasing concentration of G3BP1 was used to determine the binding of SPOP to its substrate DEK. 4 μg of His-SPOP and 0.15 μg of Myc-DEK is used in this experiment. Signal ratio of DEK/SPOP is shown below the DEK blot and signal ratio of G3BP1/SPOP is shown below the G3BP1 blot. n=3. WCL, whole cell lysate; Cyt, cytoplasmic extract; Nu, nuclear extract. Source data are provided at the end of supplementary information file.

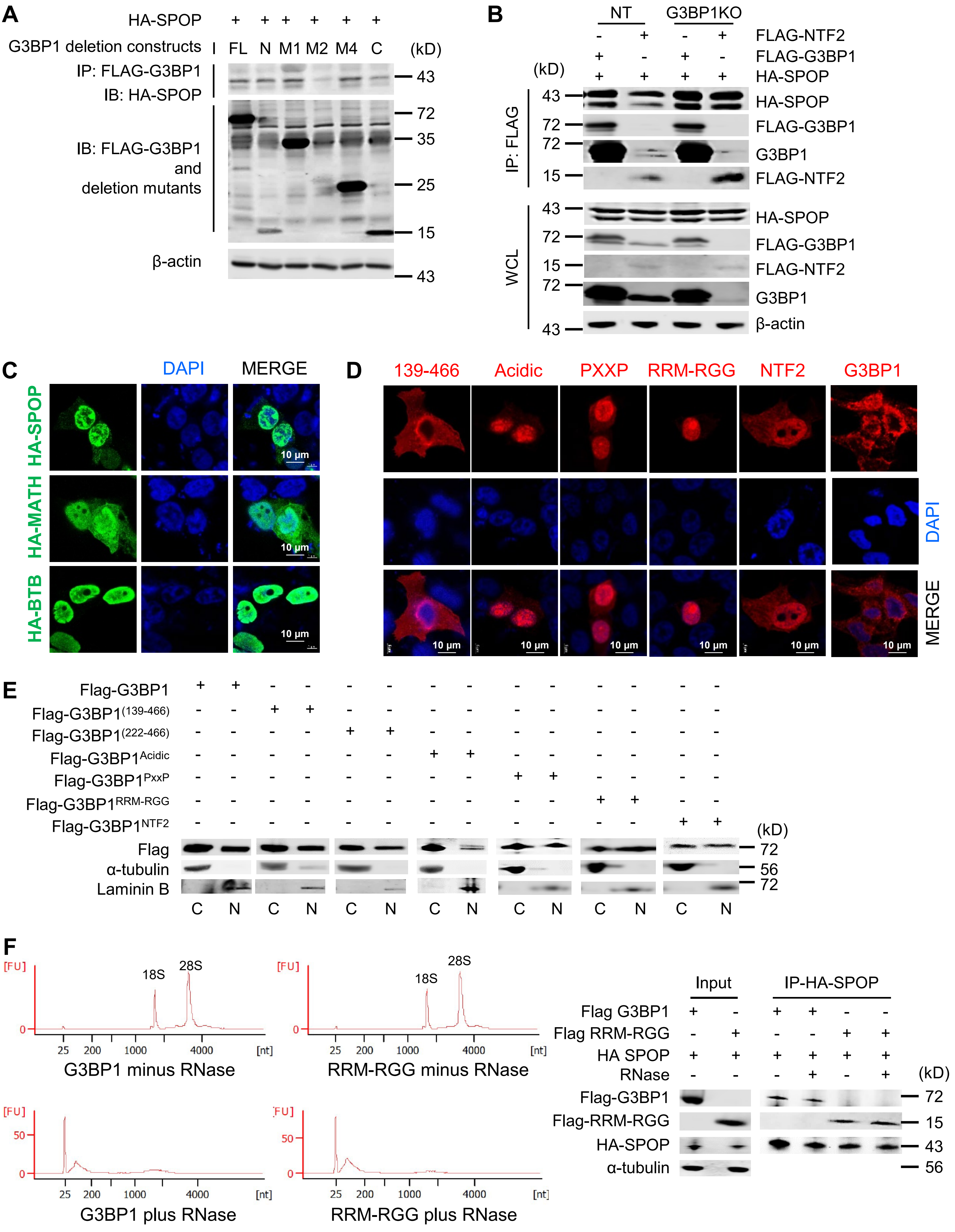

Supplementary Fig. 2

Supplementary Fig. 2. Specific domains of G3BP1 and SPOP are required for their association that is independent of G3BP1 dimerization.

(A) HEK 293T cells were transfected with HA-SPOP; and FLAG-G3BP1 and different G3BP1 deletion mutants: N: NTF2 (1-138 aa), M1: G3BP1 (139-466 aa), M4: G3BP1 (222-466 aa), M2: G3BP1 (139-338 aa), C: RRM (338-466 aa). WCL and FLAG-G3BP1 immunoprecipitates were subjected to SDS-PAGE and immunoblotting for the indicated proteins. β-actin was used as a loading control. n=3 **(B)** LNCaP-sgCtrl (NT) and LNCaP-sgG3BP1 (G3BP1 KO) cells were transiently transfected with indicated construct(s) for 48 h. Immunoblots of protein lysates and FLAG-G3BP1 pull downs were run and blotted for the indicated proteins. β-actin was used as a loading control. n=3 **(C)** Representative immunofluorescence images of 22RV1 cells transfected with HA-SPOP, HA-MATH, HA-BTB and stained with anti-HA antibody. n=3 **(D)** Representative immunofluorescence images of 22RV1 cells transfected with FLAG-G3BP1, FLAG-NTF2, FLAG-RRM, FLAG-PxxP, FLAG-Acidic, FLAG-139-466 and stained with anti-FLAG antibody. n=3 **(E)** Cytosolic (C) and nuclear (N) fraction of 22RV1 cells transfected with indicated constructs and immunoblotted with indicated antibodies. α-tubulin served as the loading control for cytoplasmic fraction and laminin B was served as a loading control for nuclear fraction n=3 **(F)** Agilent Bioanalyzer images of RNase untreated and treated samples. Presence of 18S and 28S indicates the intact RNA samples. Immunoprecipitation and immunoblotting of WCL derived from 22RV1 cells transfected with the indicated construct(s) without or with RNase treatment. n=3. Source data are provided at the end of supplementary information file. WCL, whole cell lysate.

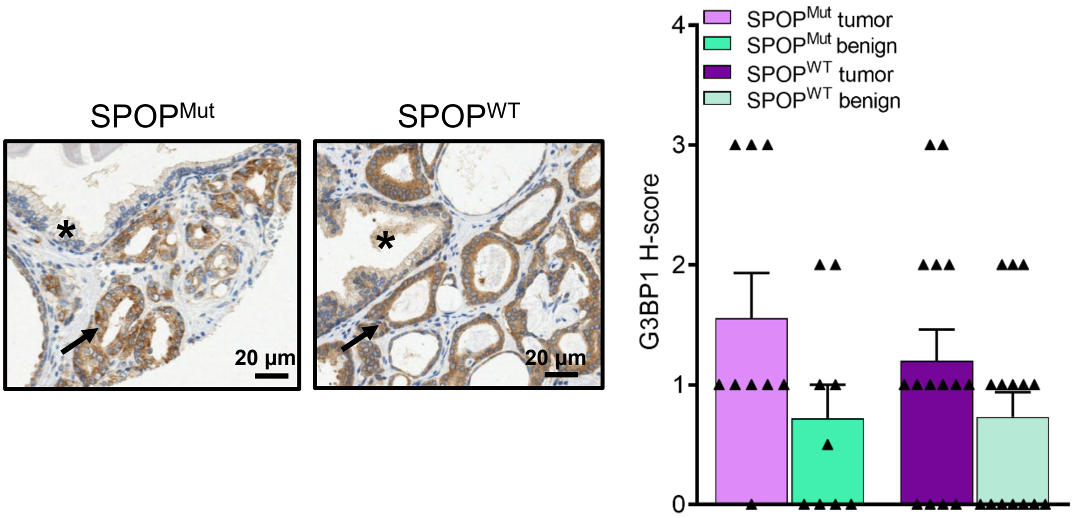

Supplementary Fig. 3

Supplementary Fig. 3. G3BP1 overexpression is independent of SPOP mutation.
Corresponding G3BP1 expression analysis on primary tumors stratified according to the $SPOP^{WT}$ (n=15 biologically independent tumor sections) and $SPOP^{Mut}$ (n=9 biologically independent tumor sections). Error bars, ± S.E.M. "star sign" indicates benign epithelium, arrow: PCa. The scale bar represents 20 µm. Source data are provided in source data file.

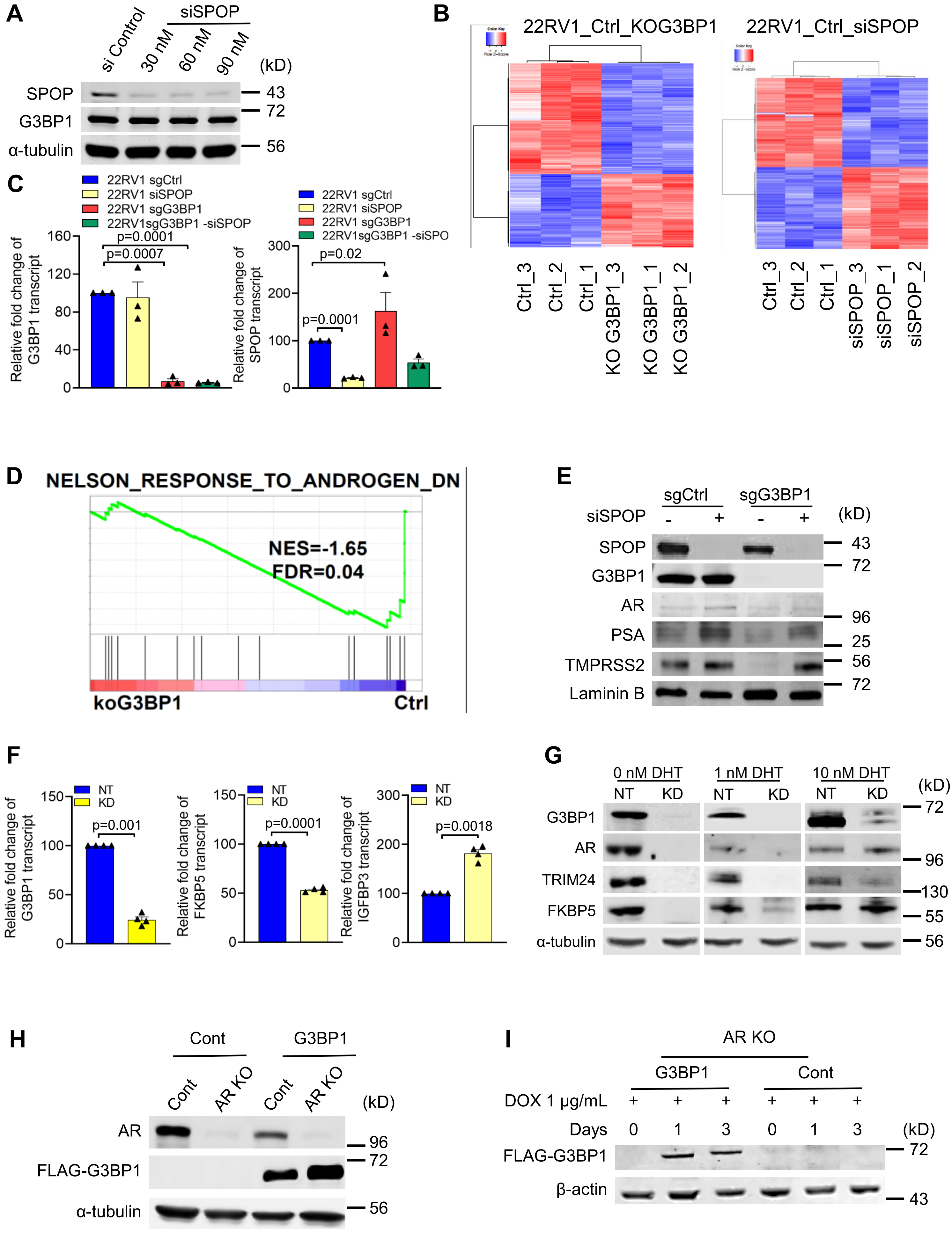

Supplementary Fig. 4

Supplementary Fig. 4. G3BP1 activates AR mediated signaling.

(A) Detection of SPOP protein after transfection with non-targeted or SPOP-siRNA at different concentrations in 22RV1 cells. α-tubulin served as a loading control. n=3 **(B)** Heatmap and hierarchical clustering of significantly differentially expressed genes of G3BP1 KO and siSPOP in 22RV1 cells. (**C**) 22RV1-sgCtrl and 22RV1-sgG3BP1 cells were transfected with non-targeted or SPOP siRNA and designated as 22RV1 sgCtrl, 22RV1 sgG3BP1, 22RV1 sgCtrl-siSPOP, or 22RV1 sgG3BP1-siSPOP, and subjected to RT-qPCR for G3BP1 and SPOP as indicated. Error bars, ± S.E.M. n=3 biologically independent experiments. Paired t-test. p value is indicated in figure. **(D)** GSEA analysis showing that genes downregulated in LNCaP cells in response to synthetic androgen R1881 are negatively enriched in G3BP1 KO cells when compared to control samples. Genes up-regulated by G3BP1 KO are negatively enriched in G3BP1 overexpressed samples compared to non-overexpressed samples in the TCGA cohort. **(E)** Immunoblot analysis of WCL derived from 22RV1-sgCtrl, 22RV1-sgG3BP1, 22RV1-sgCtrl-siSPOP, and 22RV1-sgG3BP1-siSPOP for the indicated proteins. n=3 **(F)** RT-qPCR of 3D organoids derived from DOX-inducible non-targeted (NT) and G3BP1 shRNA (G3BP1 KD) WT mPECs for the indicated genes. Error bars, ± S.E.M. n=3 biologically independent experiments. Paired t-test. p value is indicated in figure. **(G)** Organoids from NT and G3BP1 KD were grown in the presence of different doses of DHT. Immunoblot analysis was performed for the indicated proteins. α tubulin served as loading control. **(H)** Confirmation of DOX-induced overexpression of G3BP1 in AR KO mPECs. α-tubulin served as loading control. n=3 **(I)** Confirmation of AR KO and DOX-induced overexpression of G3BP1 in WT mPECs and AR KO mPECs. β-actin served as a loading control. n=3. Source data are provided at the end of supplementary information file. WCL, whole cell lysate.

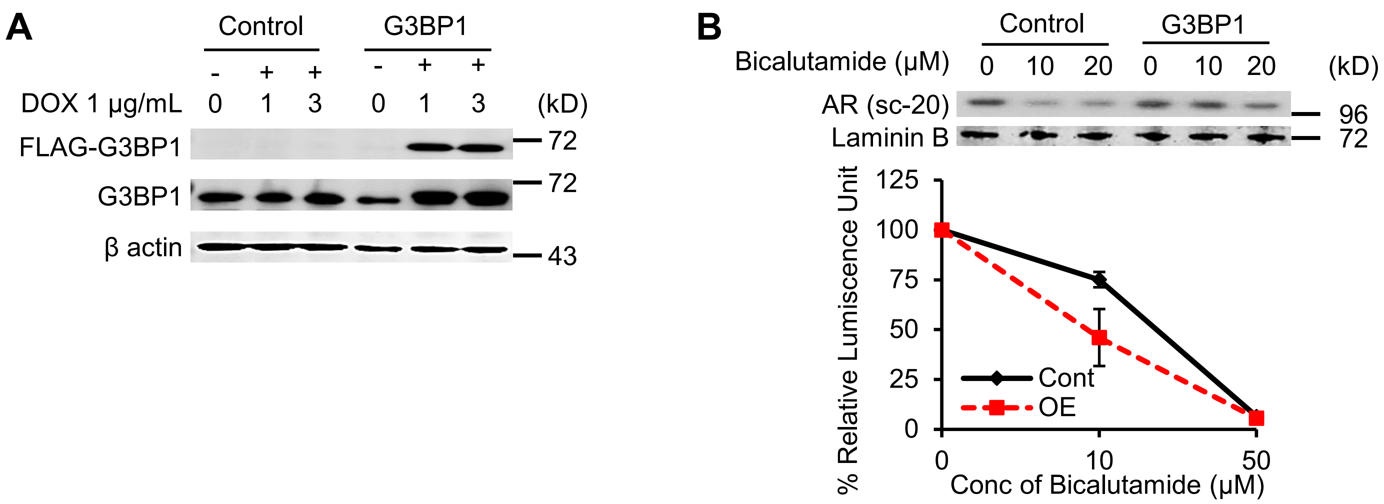

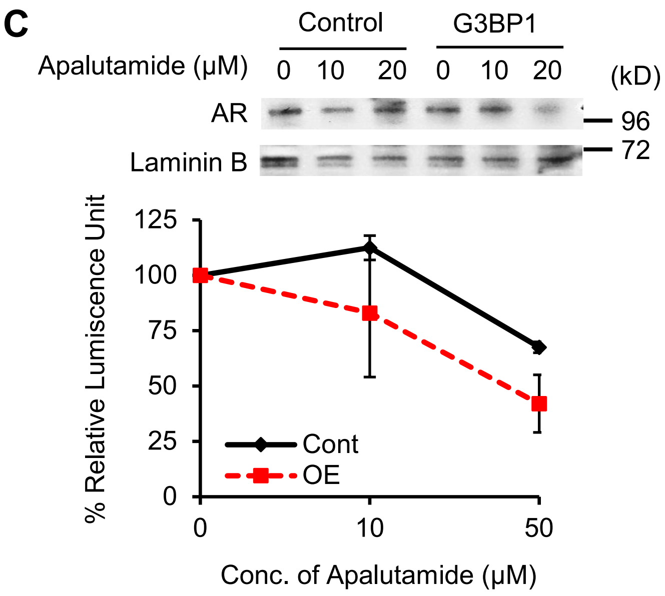

D

% Cell Death at 24 h		
Enzalutamide conc (µM)	Control	G3BP1
0	<1%	<1%
10	<1%	<1%
20	<1%	<1%
30	1%	<1%
40	<1%	1%
50	3%	5%

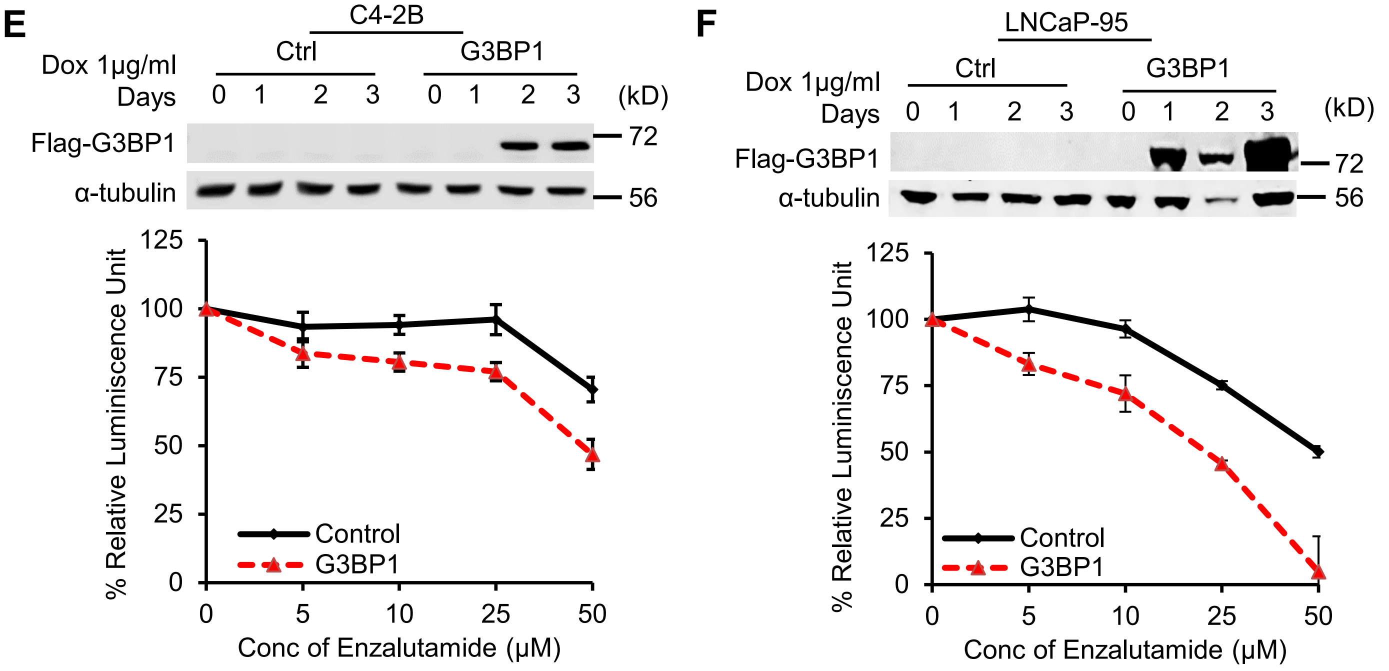

Supplementary Fig. 5

Supplementary Fig. 5. G3BP1 overexpression sensitizes 22RV1 prostate cancer cells to AR targeted drugs.

(A) Confirmation of G3BP1 overexpression after doxycycline (1 μg/mL) treatment in 22RV1 cells by immunoblotting. β actin was used as loading control. n=3 **(B-C)** Immunoblot analysis of WCL for the indicted proteins derived from DOX-inducible empty vector (Ctrl) and pCW57.1-FLAG G3BP1 (overexpressed) cells treated with bicalutamide **(B)** and apalutamide **(C)** at the indicated doses and incubated at 37°C for 24 h. Effect of bicalutamide **(B)** and apalutamide **(C)** on the survival of Ctrl and G3BP1-overexpressed 22RV1 cells. n=3 biologically independent experiments. Error bars, ± S.E.M. **(D)** Detection of percent cell death of control and G3BP1-overexpressed 22RV1 cells in response to various concentrations of enzalutamide using the trypan blue exclusion method. **(E-F)** Immunoblots of whole cell lysate derived from DOX-inducible empty vector (Ctrl) and pCW57.1-FLAG G3BP1 (overexpressed) C4-2B **(E)** and LNCaP-95 **(F)** cells. Detection of growth inhibition of Ctrl and G3BP1 overexpressed C4-2B **(E)** and LNCaP95 **(F)** cells in the presence of enzalutamide with the indicated concentrations using Cell Titer Glow assay. n=3 biologically independent experiments. Error bars, ± S.E.M. Source data are provided at the end of supplementary information file. WCL, whole cell lysate.

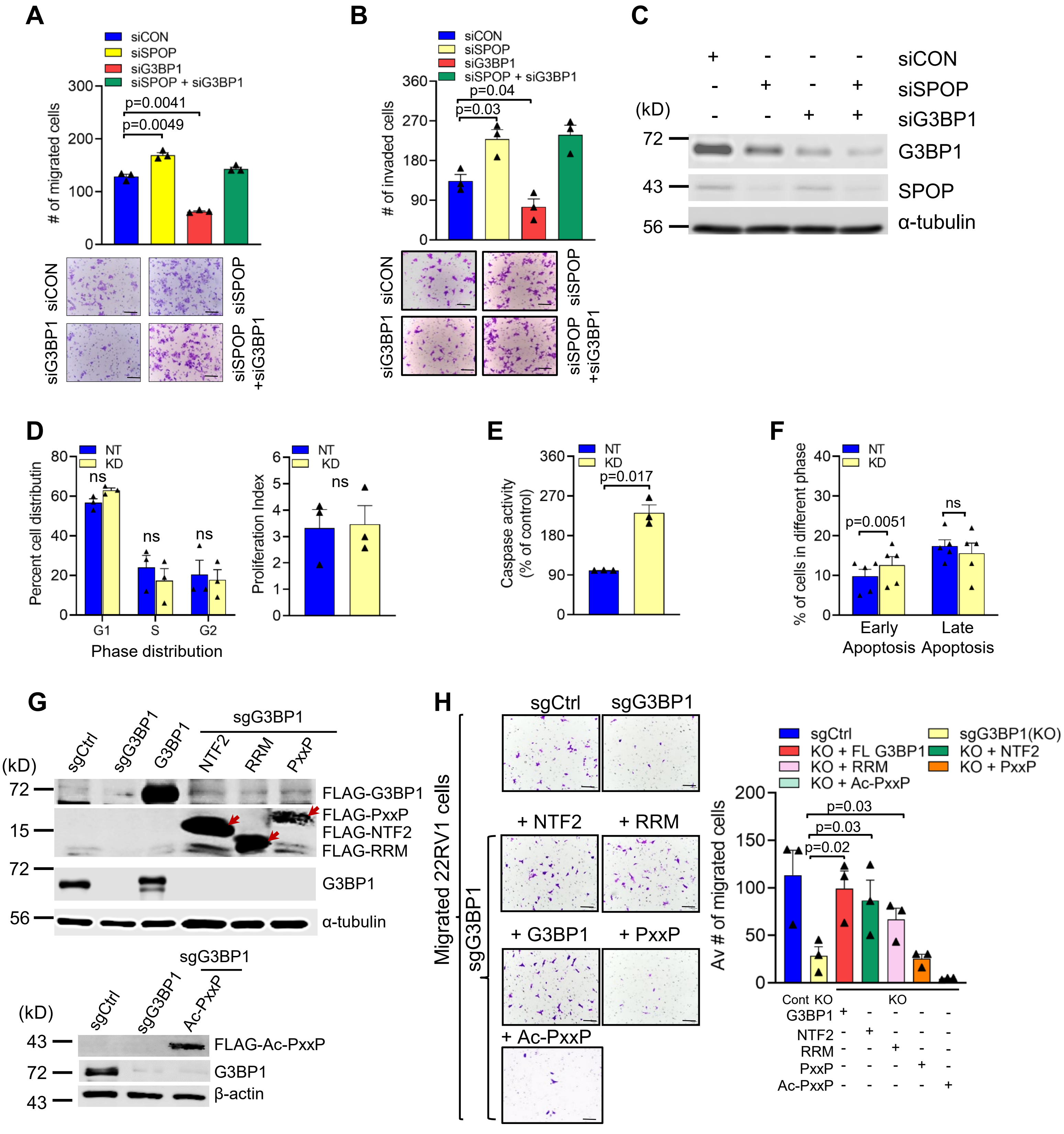

Supplementary Fig. 6

Supplementary Fig. 6. G3BP1-SPOP axis controls migratory and invasive potential with no change in proliferation for a duration of the 2-day experimentation.

(A-B) 22RV1 cells were transfected with non-targeted, SPOP siRNA, or G3BP1 siRNA or both G3BP1siRNA and SPOP siRNA and designated as 22RV1, 22RV1-siSPOP, 22RV1-siG3BP1, and 22RV1-siG3BP1-siSPOP. Quantitation and representative images of **(A)** migrated and **(B)** invaded cells. Error bars, ± S.E.M n=3 biologically independent experiments. Paired t-test. p value is indicated in figure. **(C)** 22RV1 PCa cells were transiently transfected with indicated siRNA for 48 h. Cell lysates were subjected for immunoblotting as indicated. α tubulin was used as a loading control. n=3 **(D-F)** DOX-inducible non-targeted (NT) and G3BP1 shRNA ($G3BP1^{KD}$) stable lines were generated from 22RV1 cells. **(D)** Cells were stained with DAPI and analyzed by FACS to detect the number of cells at different phases of cell cycle in NT and $G3BP1^{KD}$ cells. NT and $G3BP1^{KD}$ cells were stained with CellTrace Violet for 3 days and analyzed by FACS to assess the proliferation index. (Error bars, ± S.E.M., n=3). Paired t-test**.** **(E)** Caspase-3 and caspase-7 activities (%) were measured after 48 h in NT and $G3BP1^{KD}$ cells. Data shown represent three independent experiments, each performed in triplicate (Error bars, ± S.E.M., n=3). p value is indicated in figure. **(F)** NT and $G3BP1^{KD}$ cells were stained with Annexin V-FITC and PI to detect early ($AnnexinV^{high}$ PI^{low}) and late apoptotic ($AnnexinV^{high}$ PI^{high}) cells. (Error bars, ± S.E.M., n=3). Paired t-test **(G)** 22RV1-sgG3BP1 cells were transiently transfected with indicated construct(s) for 48 h and subjected to SAS-PAGE and immunoblotting for the indicated proteins. α-tubulin was used as a loading control. n=3 **(H)** 22RV1-sgCtrl and $^{-}$22RV1-sgG3BP1 cells were transiently transfected with the indicated construct(s) for Boyden Chamber migration assays. Quantification and representative images (10× magnifications) of migrated cells. 22RV1-sgCtrl and 22RV1-sgG3BP1 were used as references. n=3 biologically independent experiments. Error bars, ± S.E.M. Paired t-test. p value is indicated in figure. WCL, whole cell lysate. Source data including FACS sequential gating/sorting strategies for supplementary figure 6D, 6E, and 6F are provided at the end of supplementary information file.

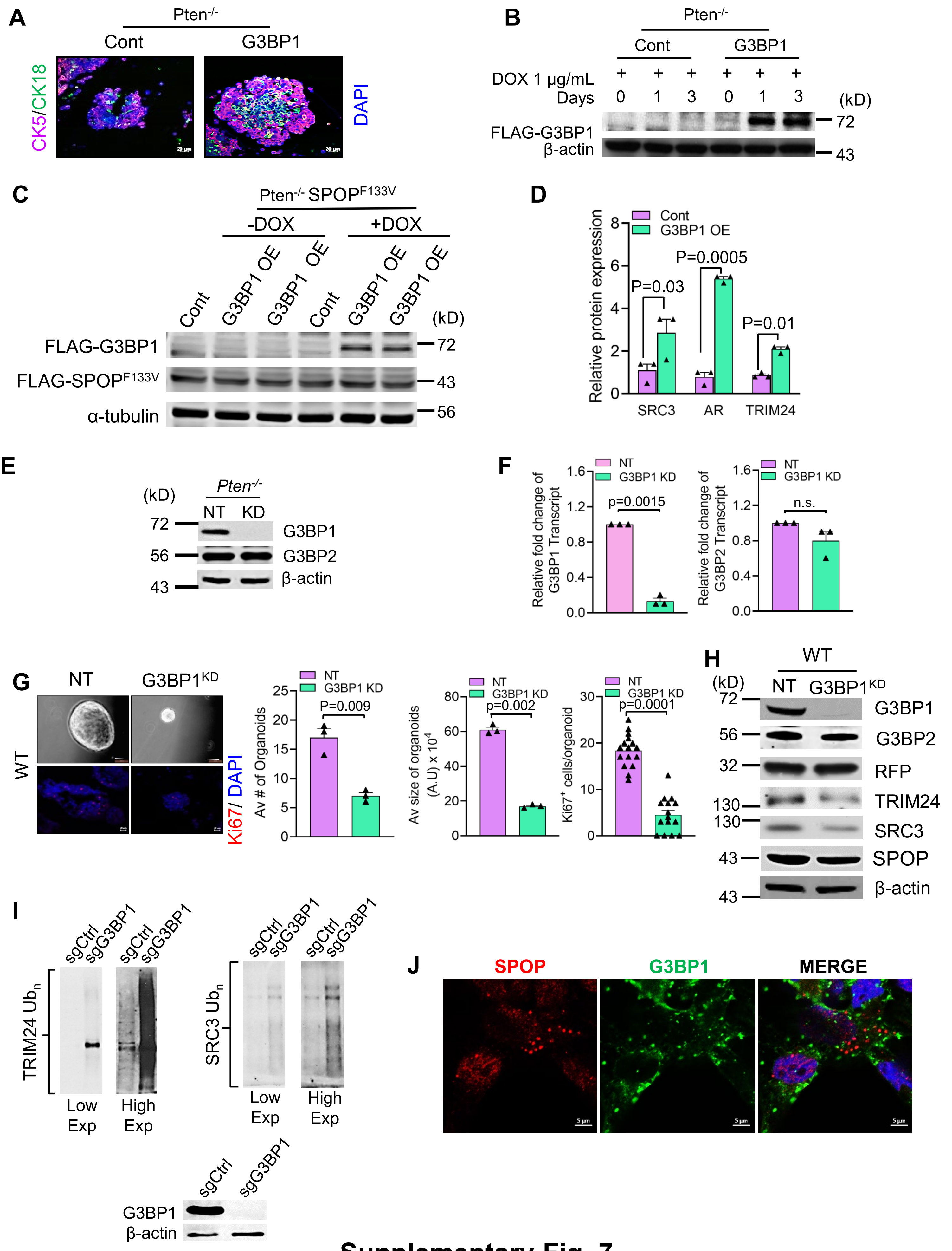

Supplementary Fig. 7

Supplementary Fig. 7. G3BP1-SPOP ubiquitin signaling axis regulates organoid growth but not stress granule formation.

(A) Characterization of G3BP1-overexpressing Pten$^{-/-}$ mPECs. Representative images of cytokeratin 5 and cytokeratin 18 per organoid are shown. scale bar, 20 µm. n=3 **(B)** Confirmation of induction of G3BP1 expression after doxycycline (1 µg/mL) treatment. α-tubulin served as a loading control. n=3 **(C)** Confirmation of G3BP1 overexpression after doxycycline (1 µg/mL) treatment at the protein level as indicated. α tubulin was used as a loading control. n=3 **(D)** For statistical analysis, band intensity (of Figure 7C) were quantified using ImageJ2. Data were normalized with housekeeping gene and average values were calculated. Error bars, ± S.E.M. n=3 biologically independent experiments. Paired t-test. **(E-F)** Expression of G3BP1 and G3BP2 at protein n=3 **(E)** and mRNA **(F)** levels n=3 biologically independent experiments. Error bars, ± S.E.M. Paired t-test. p value is indicated in figure. β-actin served as loading control. Expression of G3BP2 transcript level **(F)**. **(G)** DOX-inducible non-targeted (NT) and G3BP1 shRNA (G3BP1^{KD}) stable lines were generated from WT mPECs. Photomicrographs of representative mouse prostate organoids (scale bar, 200 µm) and Ki67 (scale bars, 20 µm) staining from the indicated mouse genotype following quantification of organoids (number and size) and Ki67. n=3 biologically independent experiments. Error bars, ± S.E.M. Paired t-test. p value is indicated in figure. **(H)** Representative immunoblots showing SPOP substrates in WT mPECs. β-actin served as loading control. n=3 **(I)** 22RV1 sgCtrl and sgG3BP1 cells were transiently transfected with His-ubiquitin construct for 48 h. Ni-NTA pull-down products were immunoblotted with TRIM24 and SRC 3. WCL were blotted for the indicated proteins. β-actin serves as the loading control. n=3 **(J)** SPOP is not present in the stress granules where cytoplasmic G3BP1 resides. Representative immunofluorescence images of LNCaP cells treated with 0.5 mM sodium arsenite for 30 mins and stained with G3BP1 (green) and SPOP (red) antibodies. n=3. scale bar, 5 µm. Source data are provided at the end of supplementary information file.

Immunoblot images depicted in Supplementary Fig. 1B

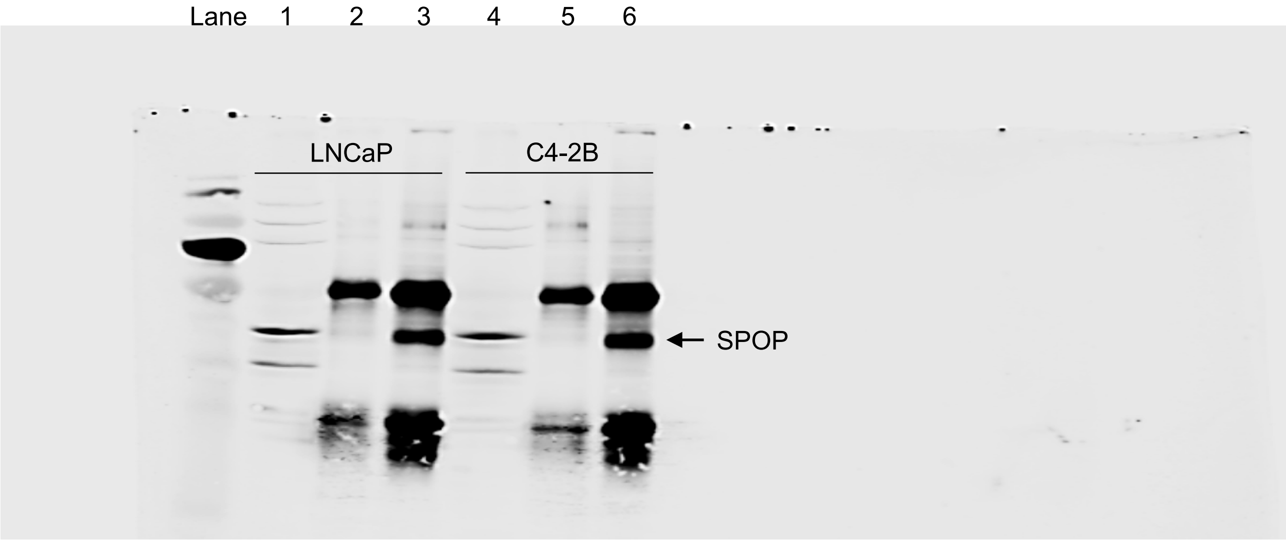

Lane 1: Whole cell lysate
Lane 2: IgG control
Lane 3: Pull down with endogenous G3BP1 and immunoblotted with endogenous SPOP
Lane 4: Whole cell lysate
Lane 5: IgG control
Lane 6: Pull down with endogenous G3BP1 and immunoblotted with endogenous SPOP

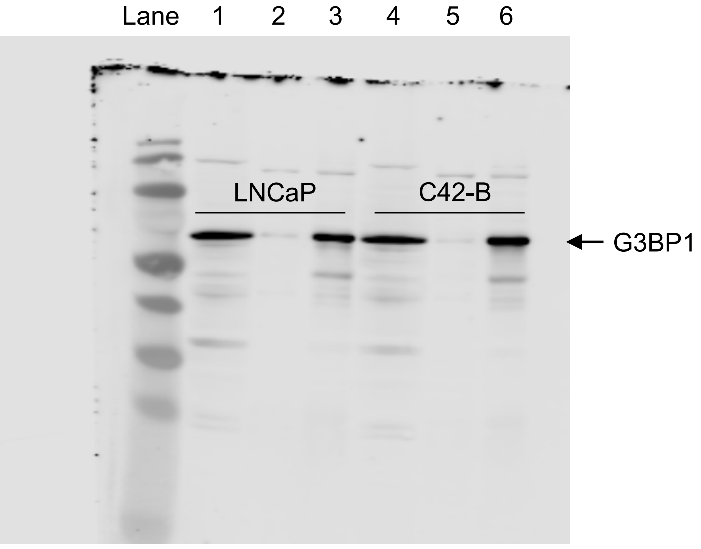

Lane 1: Whole cell lysate
Lane 2: IgG control
Lane 3: Pull down with endogenous G3BP1 and immunoblotted with endogenous G3BP1
Lane 4: Whole cell lysate
Lane 5: IgG control
Lane 6: Pull down with endogenous G3BP1 and immunoblotted with endogenous G3BP1

Immunoblot images depicted in Supplementary Fig. 1C

Lane 1 2

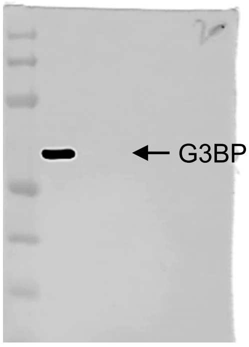

← G3BP1

Lane 1: sgControl
Lane 2: sgG3BP1

Lane 1 2

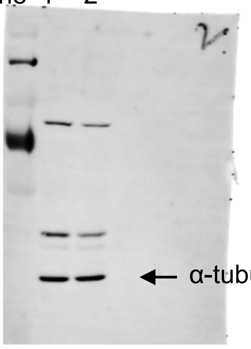

← α-tubulin

Lane 1: sgControl
Lane 2: sgG3BP1

Immunoblot images depicted in Supplementary Fig. 1E

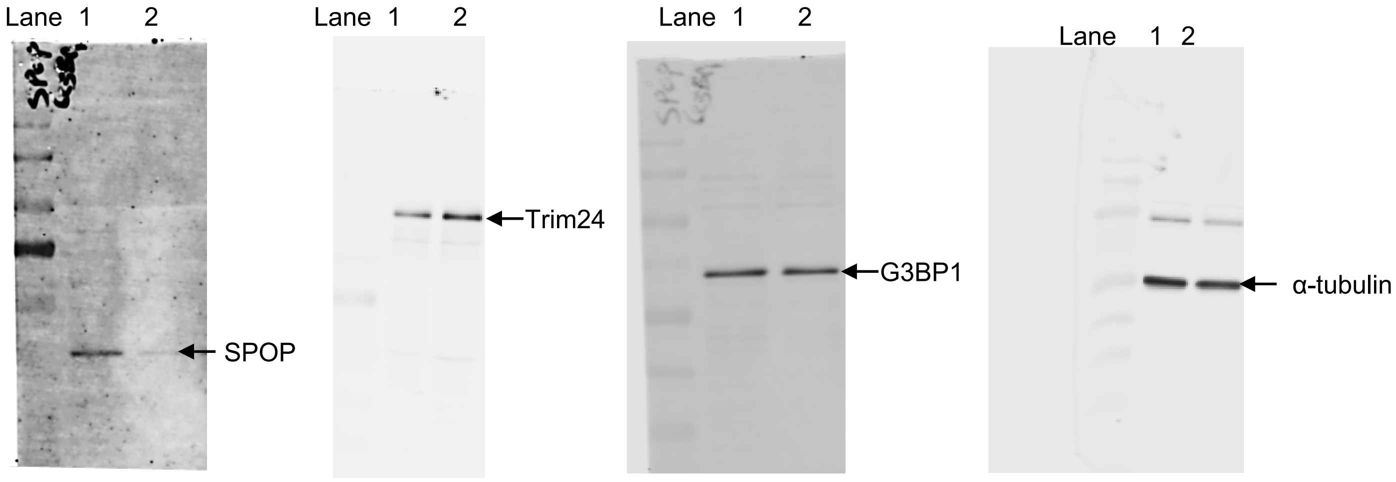

Lane 1: siControl
Lane 2: siSPOP

Immunoblot images depicted in Supplementary Fig. 1F

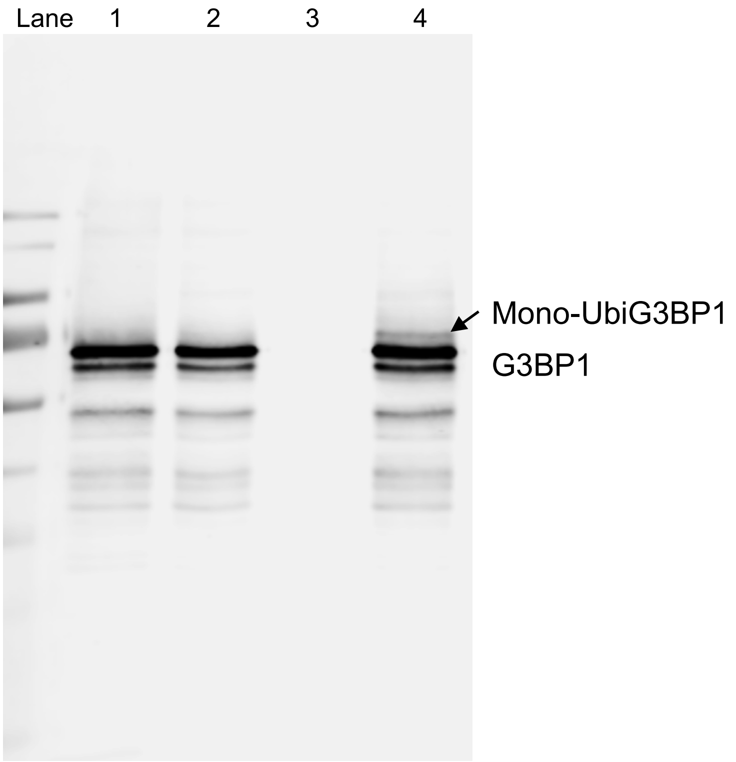

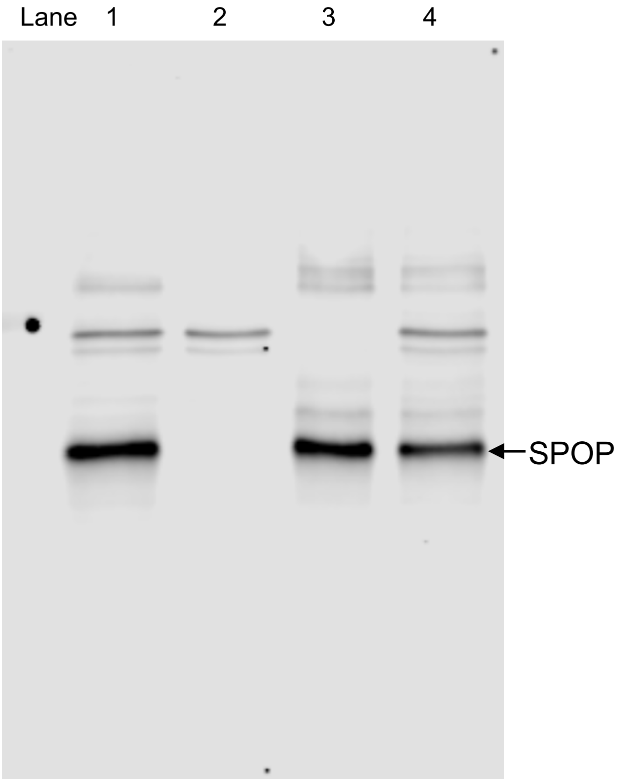

Lane 1: Ub+E1 and E2+ His SPOP+ His G3BP1
Lane 2: Ub+E1 and E2+ Nedd CUL3/Rbx1 + His G3BP1
Lane 3: Ub+E1 and E2+ Nedd CUL3/Rbx1 + His SPOP
Lane 4: Ub+E1 and E2+ Nedd CUL3/Rbx1 + His G3BP1+ His SPOP

Immunoblot images depicted in Supplementary Fig. 1G

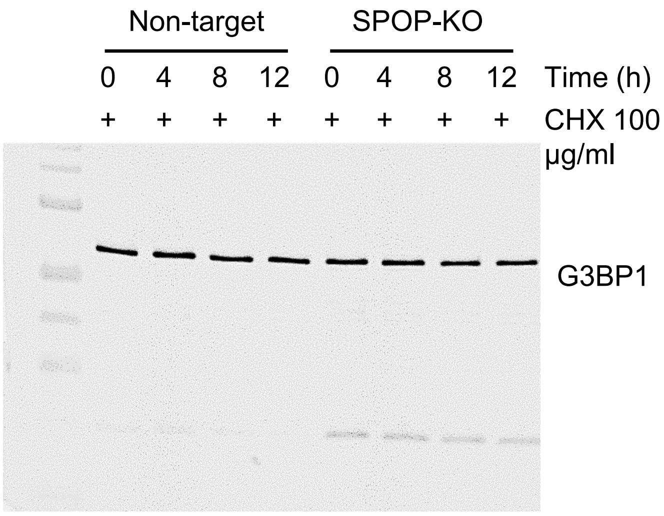

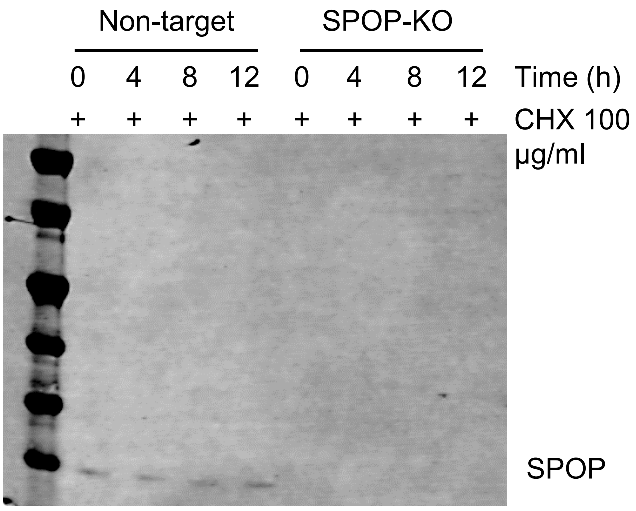

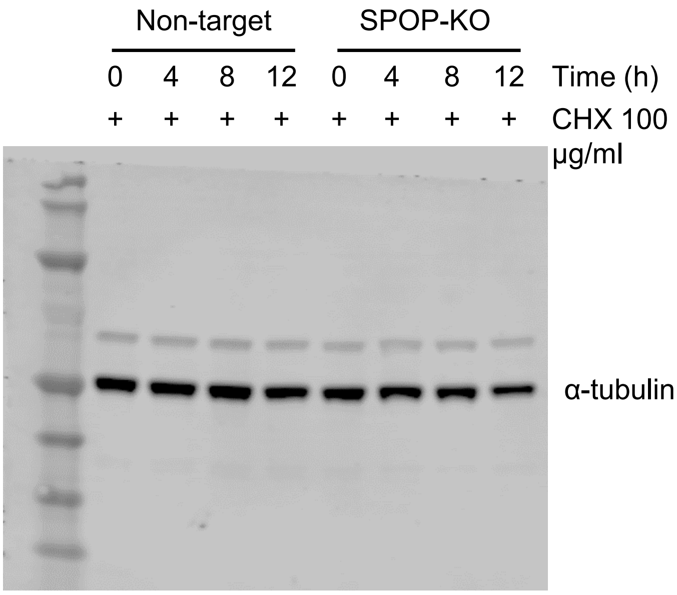

Immunoblot images depicted in Supplementary Fig. 1H

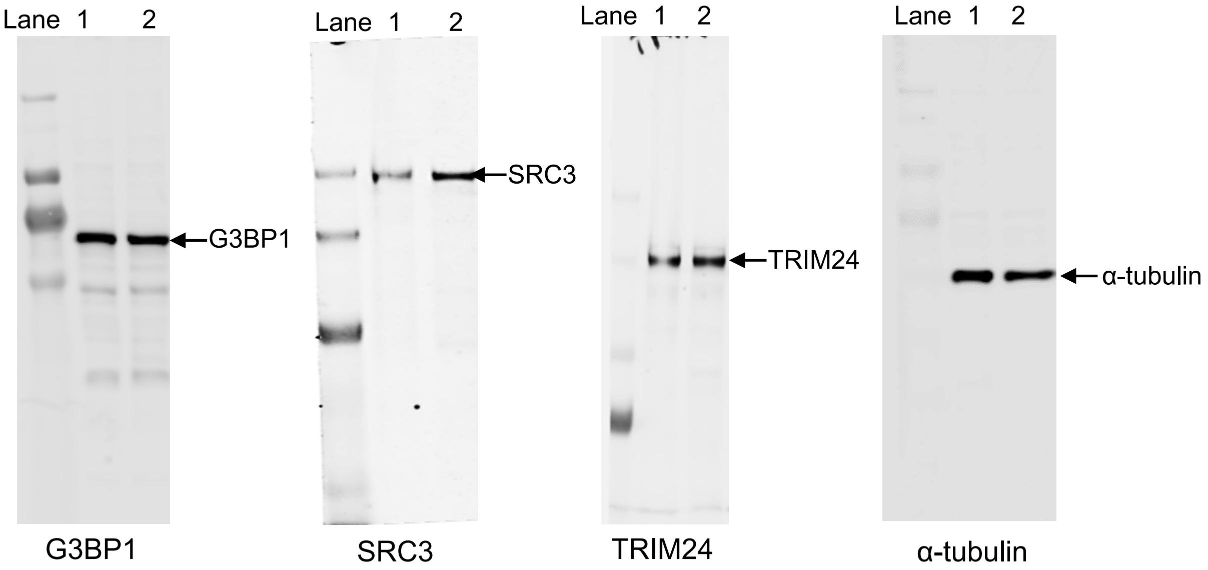

Lane 1: 22RV1 cells treated with DMSO
Lane 2: 22RV1 cells treated with 10μM MG132

Immunoblot images depicted in Supplementary Fig. 1K

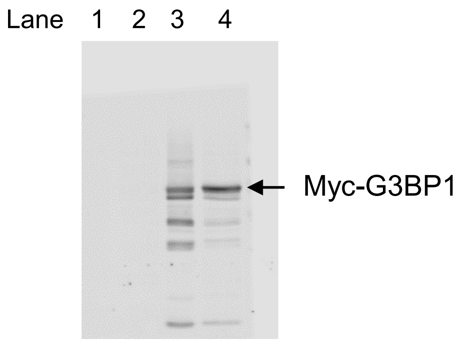

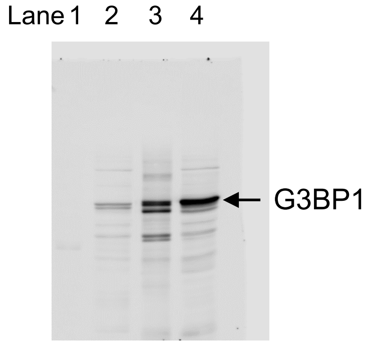

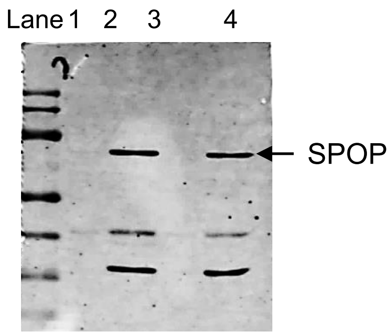

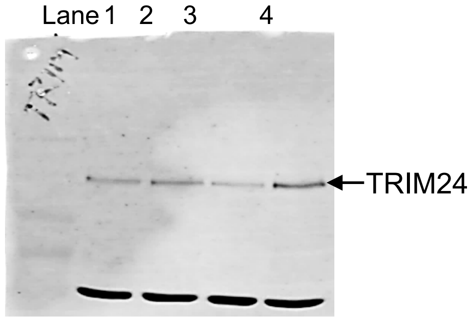

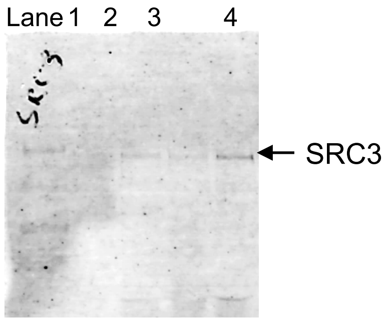

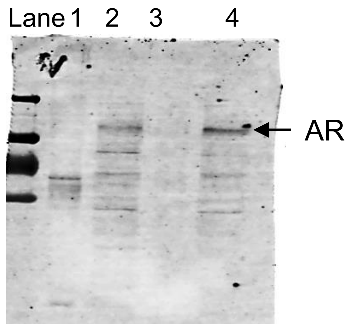

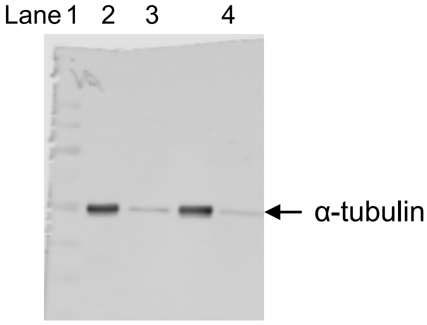

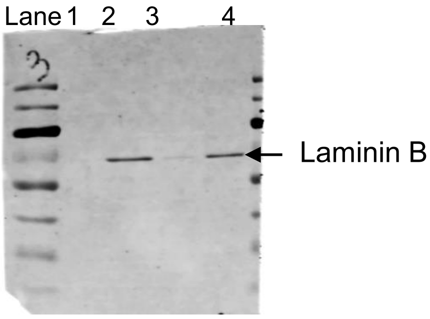

Lane 1: Cytosolic extract: sgControl
Lane 2: Nuclear extract: sgControl
Lane 3:Cytosolic extract: sgG3BP1
Lane 4: Nuclear extract: sgG3BP1

Immunoblot images depicted in Supplementary Fig. 1L

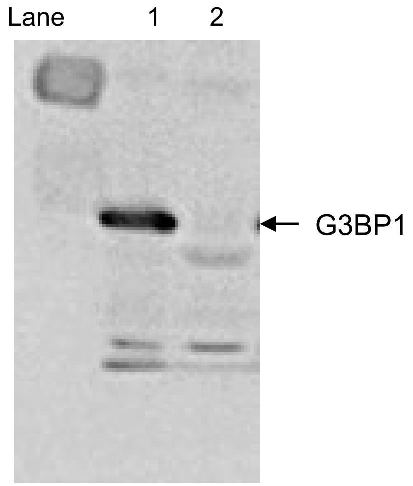

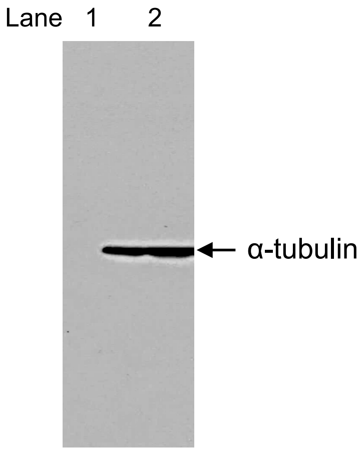

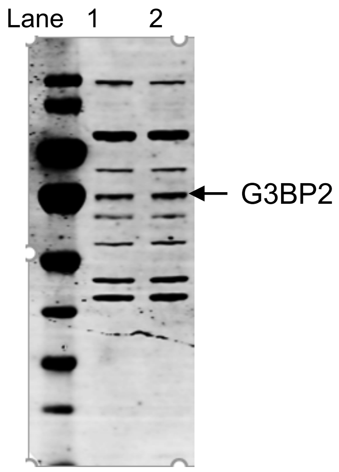

Lane 1: sgControl
Lane 2: sgG3BP1

Immunoblot images depicted in Supplementary Fig. 1M

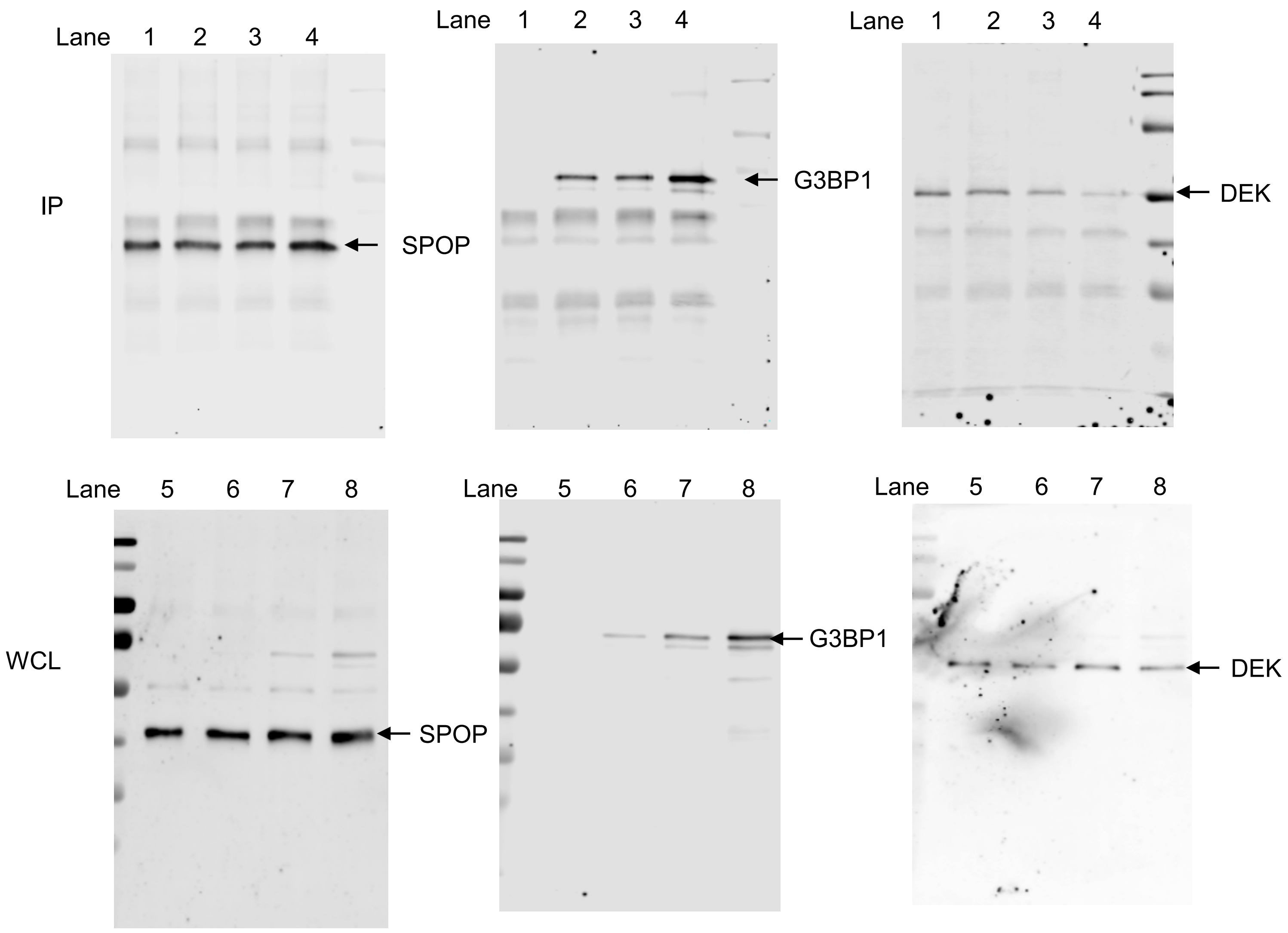

IP:
Lane 1: His SPOP + Myc DEK
Lane 2: His SPOP + His G3BP1(0.125µg) + Myc DEK
Lane 3: His SPOP + His G3BP1(0.25 µg) + Myc DEK
Lane 4: His SPOP + His G3BP1(0.75 µg) + Myc DEK
Immunoblot:
Lane 4: His SPOP + Myc DEK
Lane 5: His SPOP + His G3BP1(0.125µg) + Myc DEK
Lane 6: His SPOP + His G3BP1(0.25 µg) + Myc DEK
Lane 7: His SPOP + His G3BP1(0.75 µg) + Myc DEK

Immunoblot images depicted in Supplementary Fig. 2A

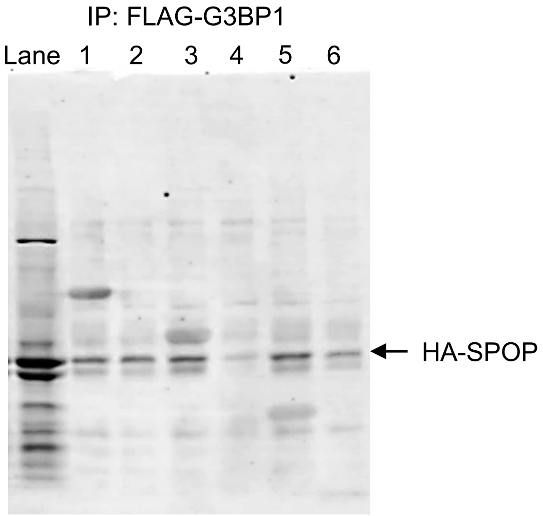

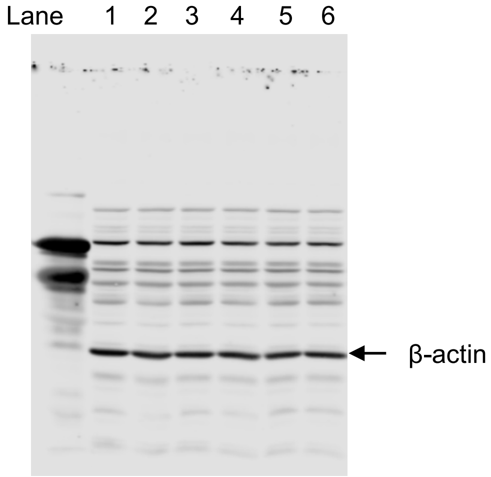

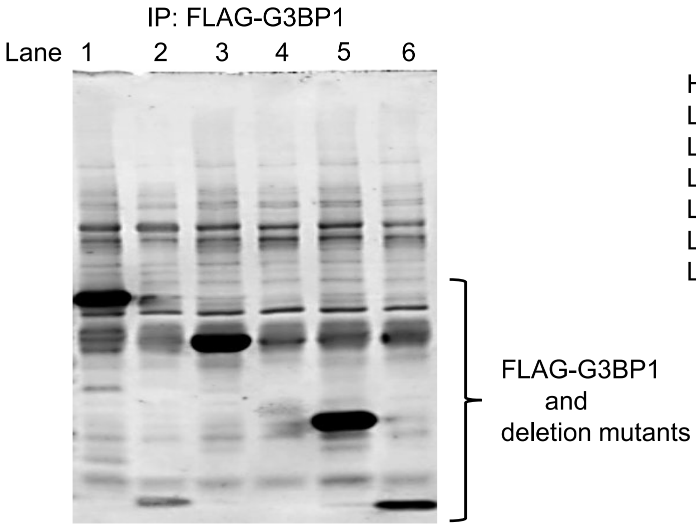

HEK 293T cells were transfected with
Lane 1: HA-SPOP and Flag-G3BP1
Lane 2: HA-SPOP and Flag-G3BP1^{NTF2}
Lane 3: HA-SPOP and Flag-G3BP1^{M1}
Lane 4: HA-SPOP and Flag-G3BP1^{M2}
Lane 5: HA-SPOP and Flag-G3BP1^{M4}
Lane 6: HA-SPOP and Flag-G3BP1^{C}

Immunoblot images depicted in Supplementary Fig. 2B

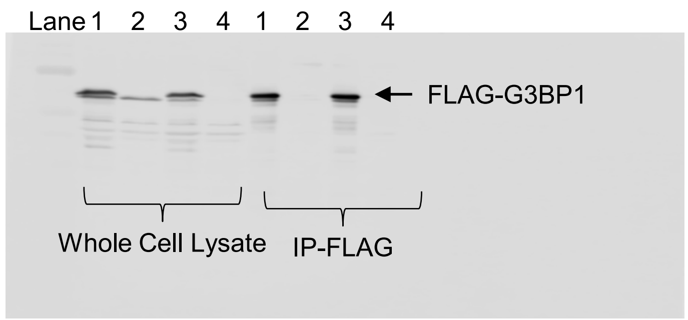

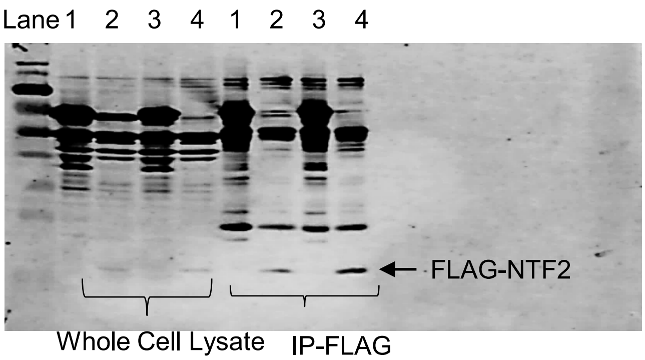

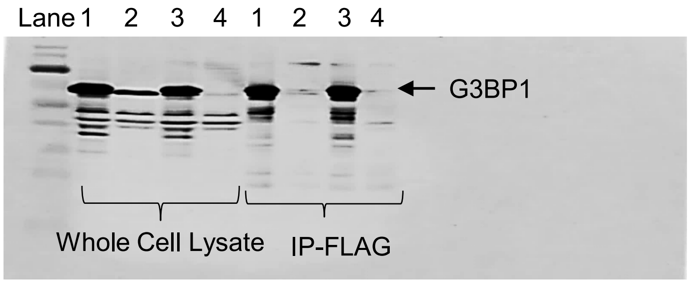

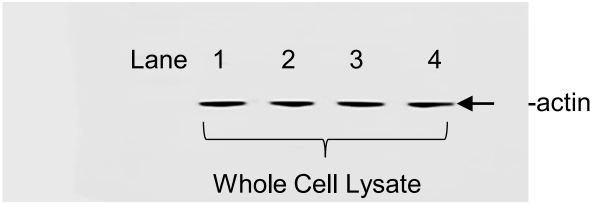

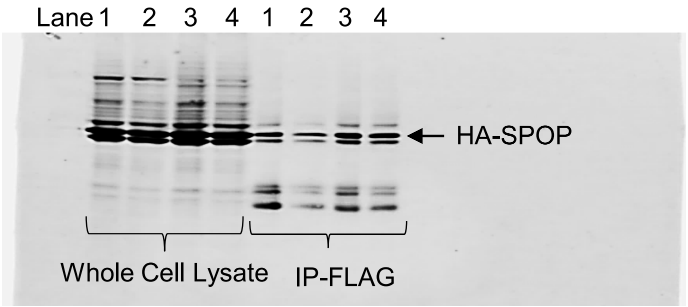

Lane 1:sgCntrol+ HA-SPOP + Flag-G3BP1
Lane 2: sgCntrol+ HA-SPOP + Flag-G3BP1^{NTF2}
Lane 3: sgG3BP1+ HA-SPOP + Flag-G3BP1
Lane 4: sgG3BP1+ HA-SPOP + Flag-G3BP1^{NTF2}

Immunoblot images depicted in Supplementary Fig. 2E

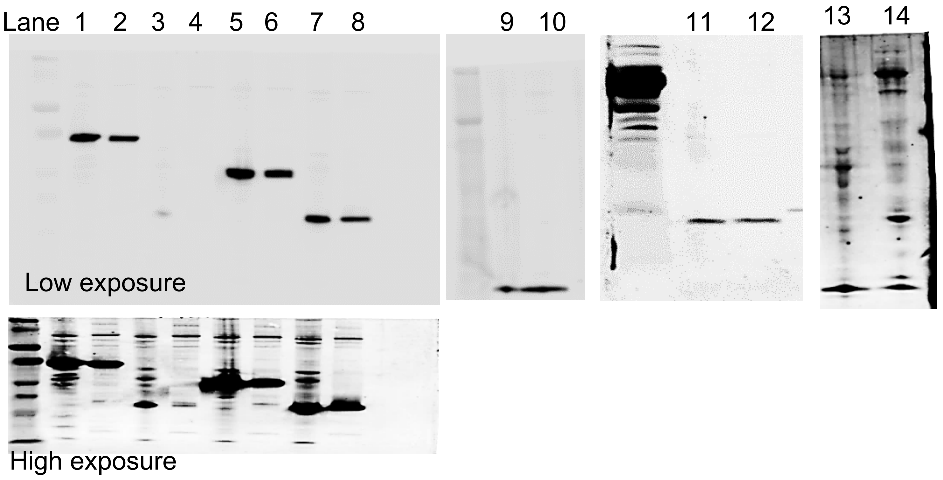

Flag-G3BP1 and its deletion construct, cytosolic and nuclear extract

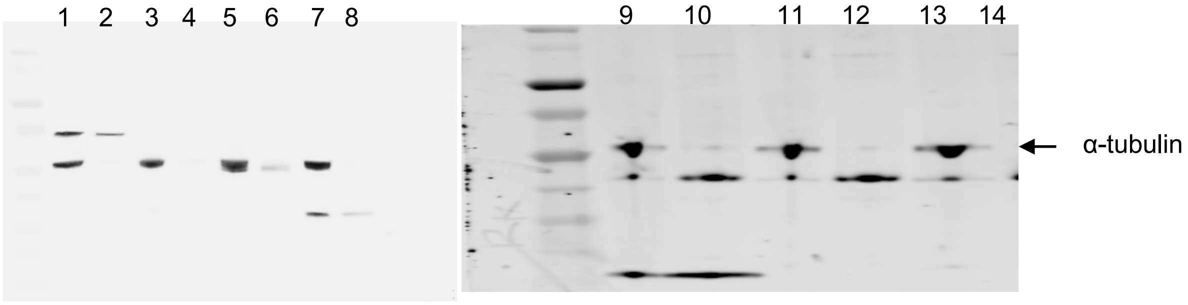

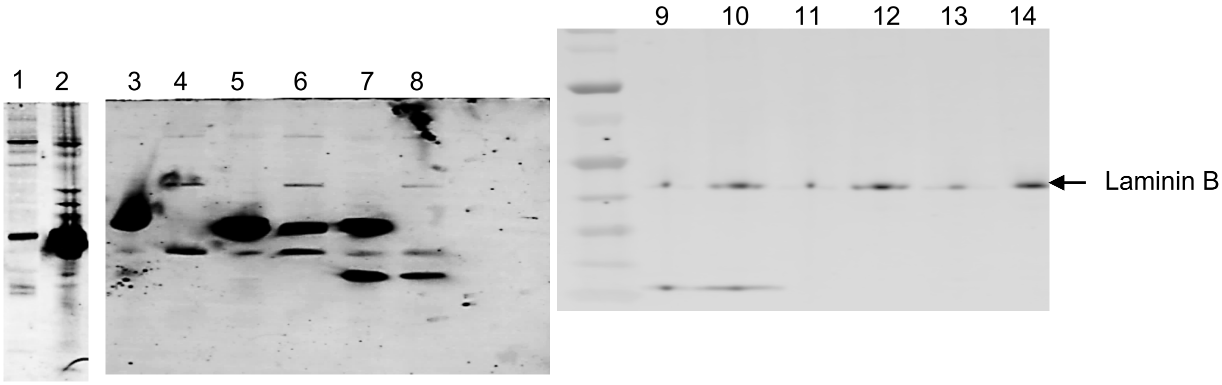

22RV1 cells were transfected with
Lane 1: full length G3BP1;cytosolic extract
Lane 2: full length G3BP1; nuclear extract
Lane 3: Flag-G3BP1$^{(139\text{-}466)}$;cytosolic extract
Lane 4: Flag-G3BP1$^{(139\text{-}466)}$;nuclear extract
Lane 5: Flag-G3BP1$^{(222\text{-}466)}$; cytosolic extract
Lane 6: Flag-G3BP1$^{(222\text{-}466)}$; nuclear extract
Lane 7: Flag-G3BP1^{Acidic}; cytosolic extract
Lane 8: Flag-G3BP1^{Acidic};nuclear extract
Lane 9: Flag-G3BP1^{PxxP}; cytosolic extract
Lane 10: Flag-G3BP1^{PxxP}; nuclear extract
Lane 11: Flag-G3BP1^{RRM}; cytosolic extract
Lane 12: Flag-G3BP1^{RRM}; nuclear extract
Lane 13: Flag-G3BP1^{NTF2}; cytosolic extract
Lane 14: Flag-G3BP1^{NTF2}; nuclear extract

Immunoblot images depicted in Supplementary Fig. 2F

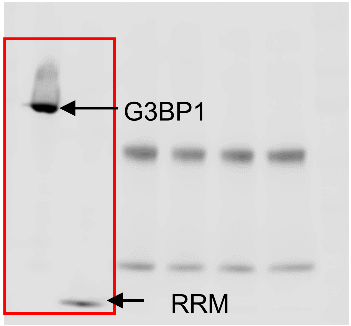

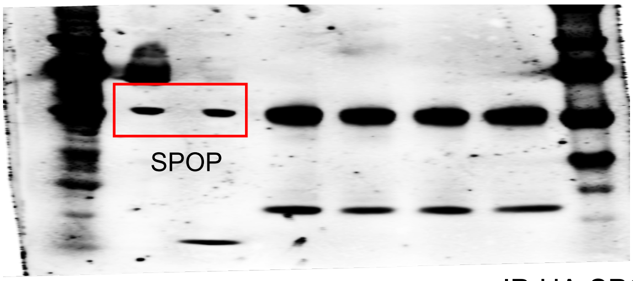

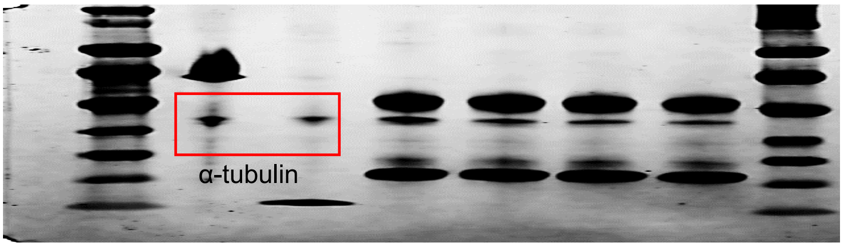

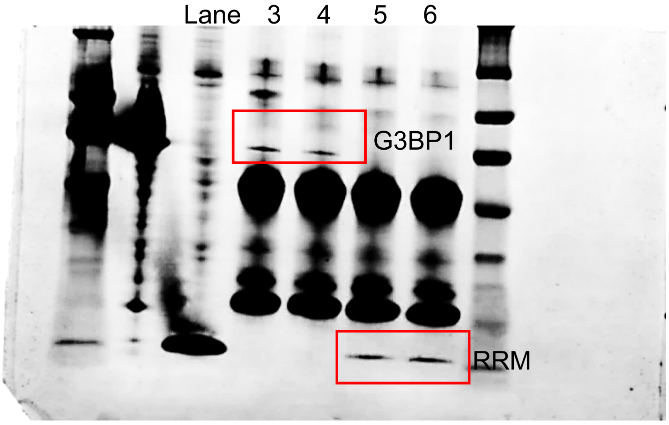

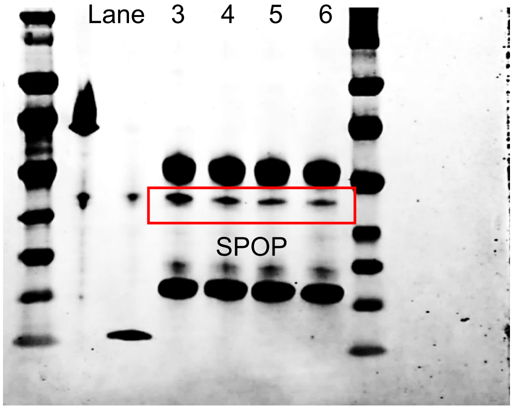

Lane 1: Whole cell lysate full length G3BP1
Lane 2: Whole cell lysate G3BP1^{RRM}
Lane 3: IP-HA-SPOP; -RNAse full length G3BP1
Lane 4: IP-HA-SPOP; +RNAse full length G3BP1
Lane 5: IP-HA-SPOP; -RNAse G3BP1^{RRM}
Lane 6: IP-HA-SPOP; +RNAse G3BP1^{RRM}

Immunoblot images depicted in Supplementary Fig. 4A

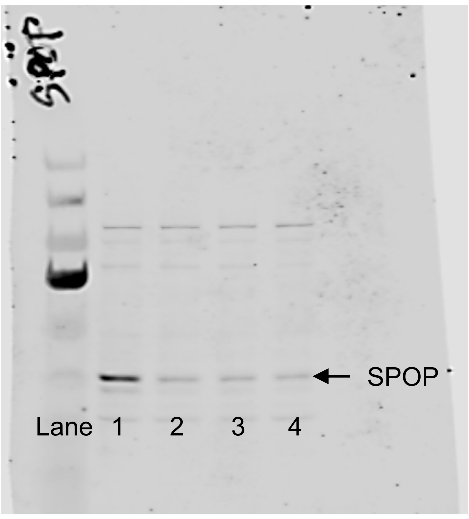

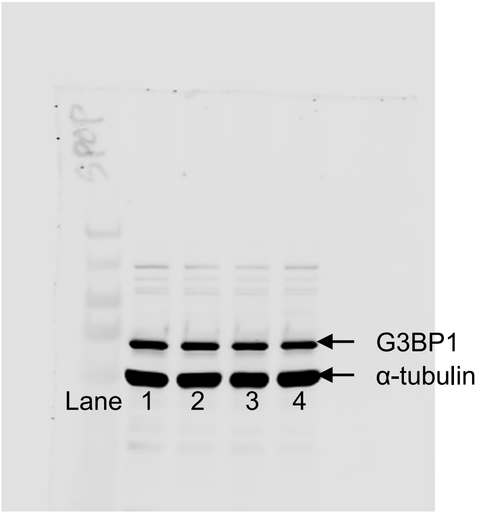

22RV1 cells were transfected with SPOP siRNA
Lane 1: siControl
Lane 2 : 30nM siSPOP
Lane 3: 60nM siG3BP1
Lane 4: 90nM siSPOP+siG3BP1

Immunoblot images depicted in Supplementary Fig. 4E

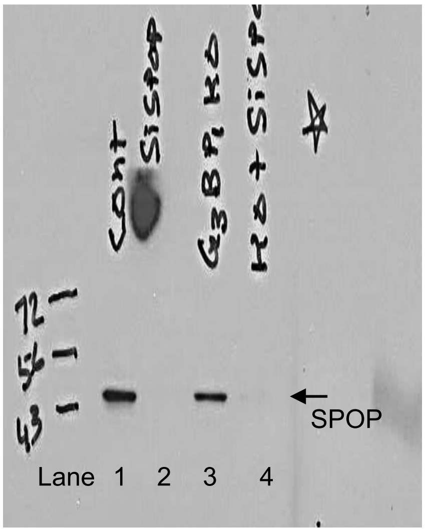

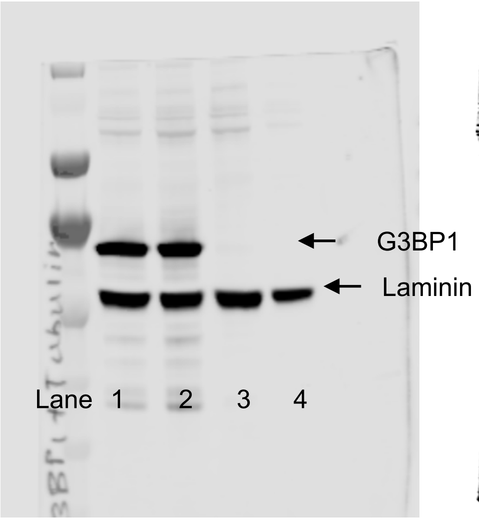

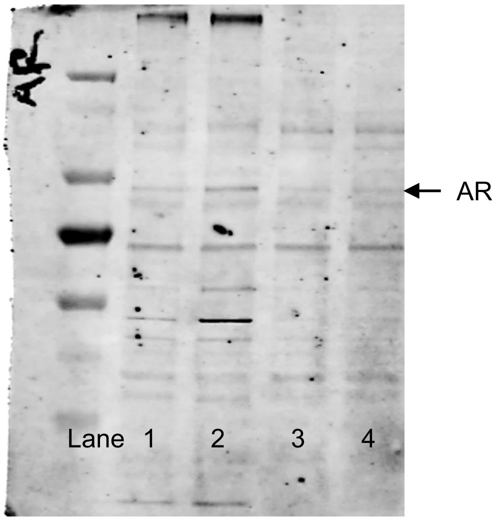

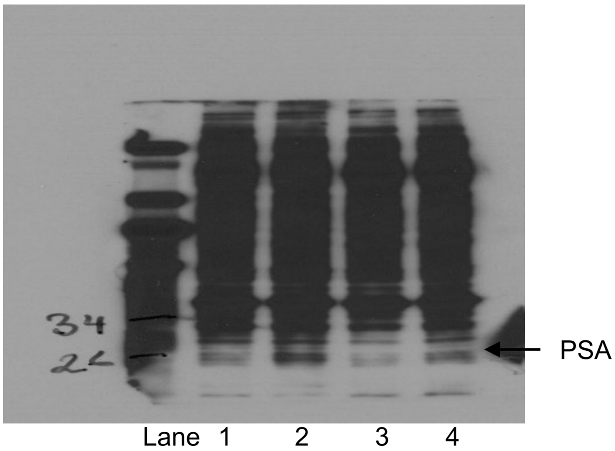

Lane 1: siControl
Lane 2 : siControl + siSPOP
Lane 3: si control+ sgG3BP1
Lane 4: siSPOP+sgG3BP1

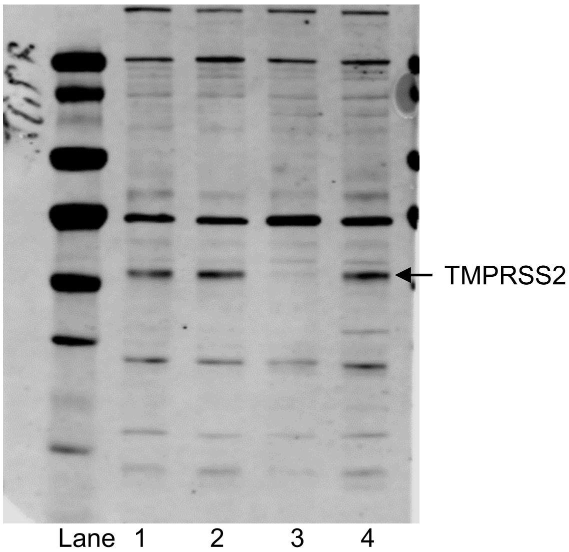

Immunoblot images depicted in Supplementary Fig. 4G

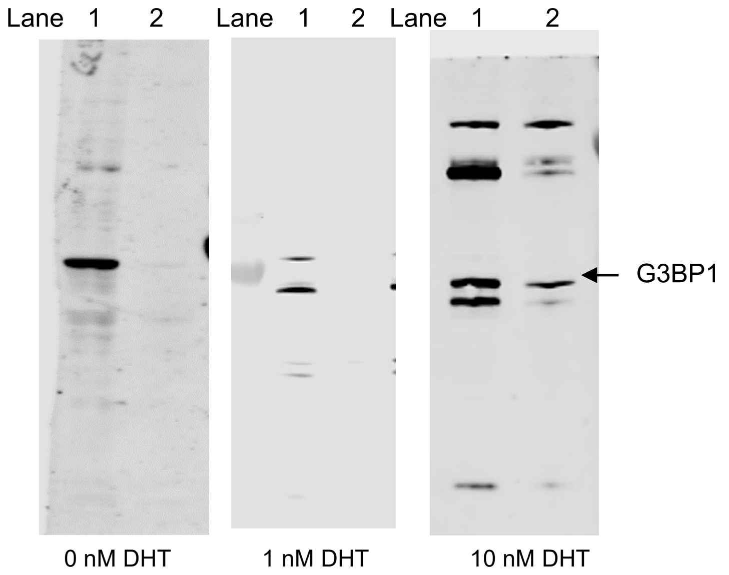

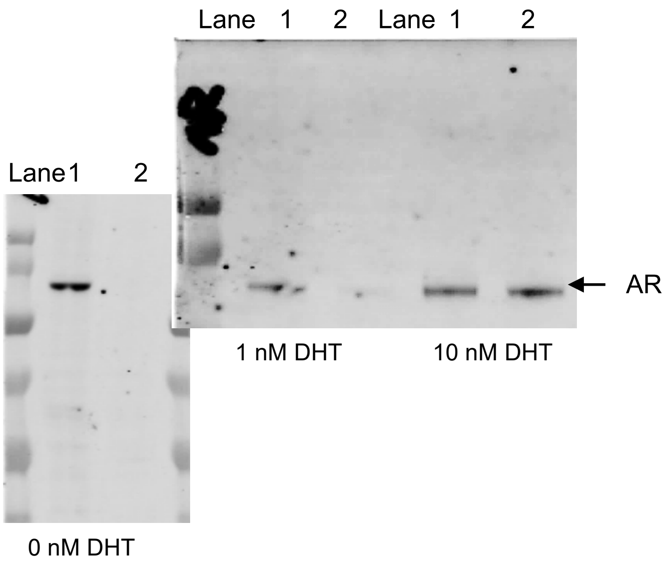

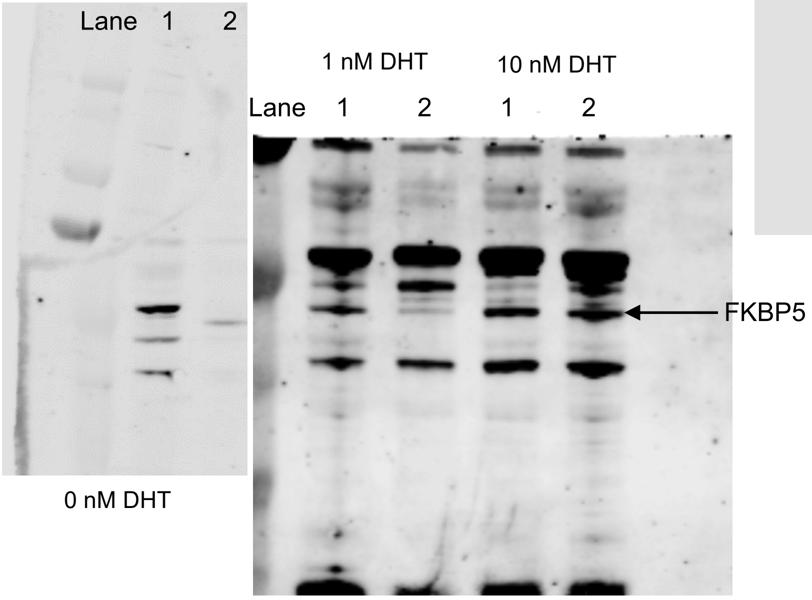

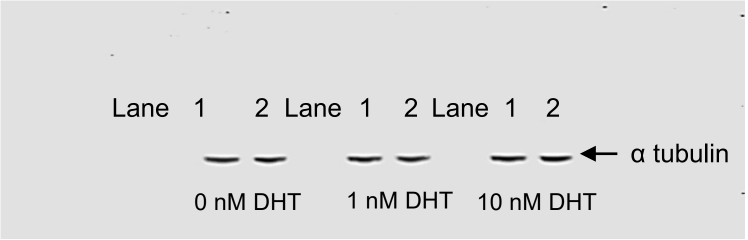

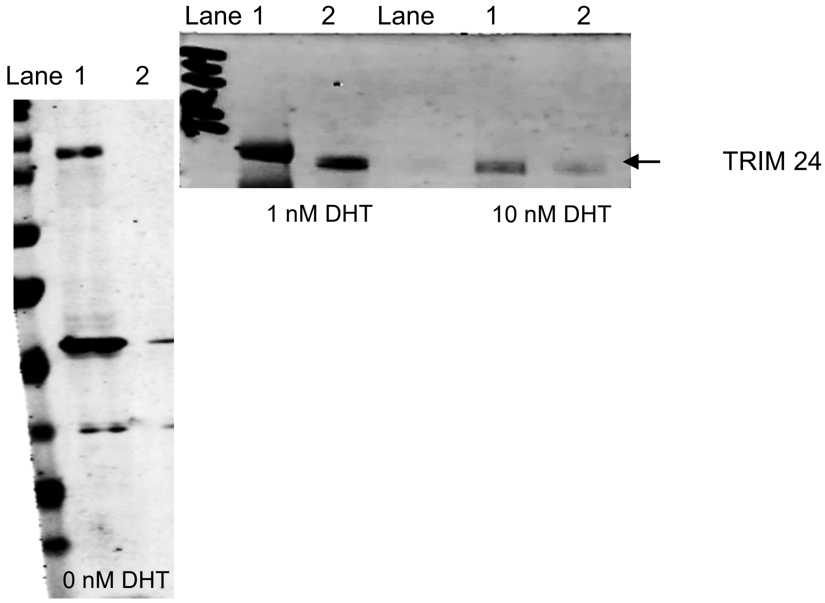

Protein lysates were collected from WT primary mouse prostate epithelial cell generated organoids.
Lane 1: Cells infected with NonTarget shRNA
Lane 2 : Cells infected with G3BP1^{KD} shRNA.

Immunoblot images depicted in Supplementary Fig. 4H

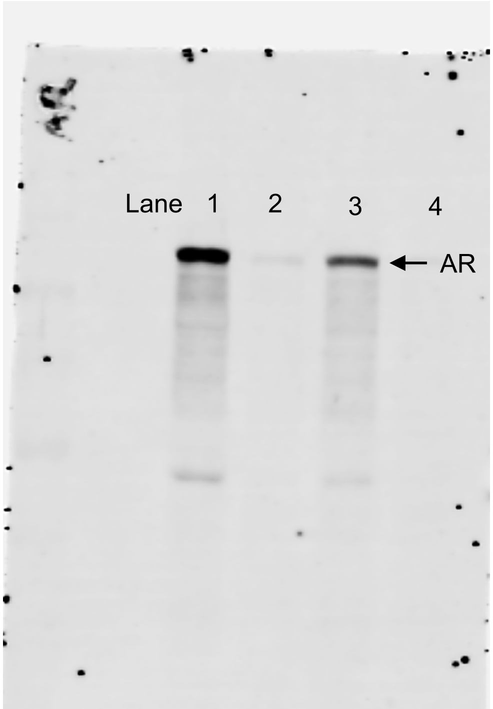

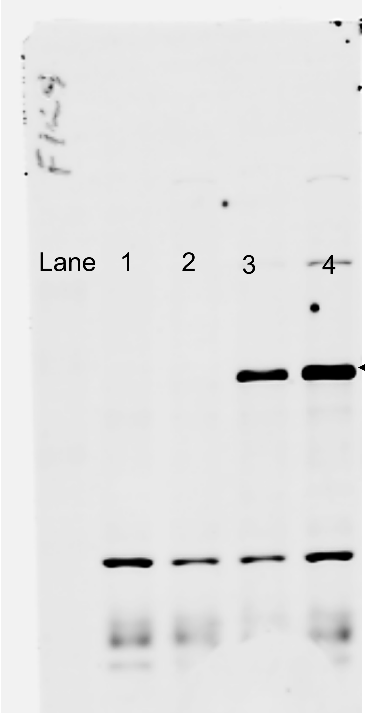

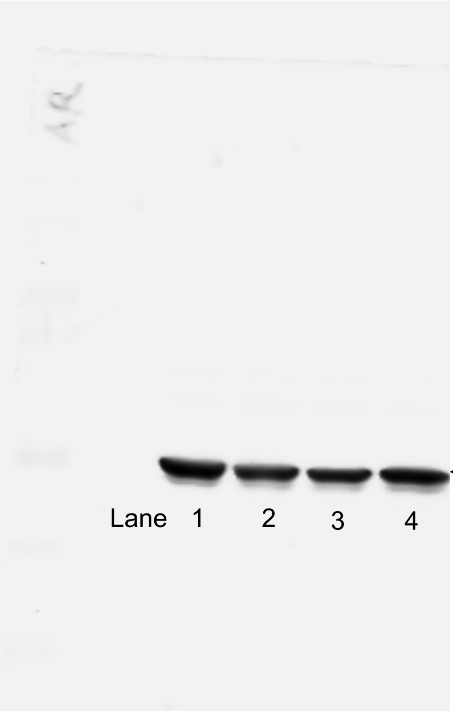

Protein lysates were collected from WT primary mouse prostate epithelial cell generated organoids.

Lane 1: sgControl

Lane 2 : sgARKO.

Lane 3: G3BP1 overexpressed

Lane 4: sgARKO+ G3BP1 overexpressed

Immunoblot images depicted in Supplementary Fig. 4I

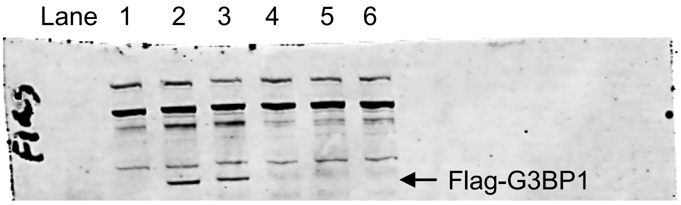

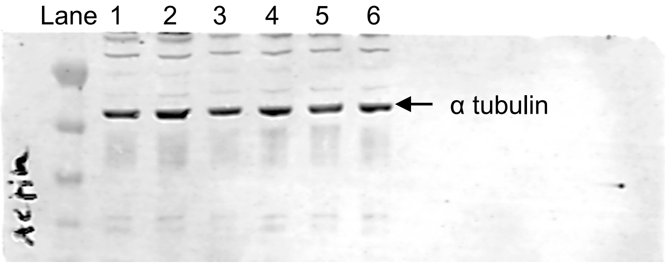

Protein lysates were collected from WT primary mouse prostate epithelial cell generated organoids.
Lane 1: AR KO + G3BP1 overexpressed 1 ug/ml dox treated with 0 days
Lane 2 : AR KO + G3BP1 overexpressed 1 ug/ml dox treated with 1 day.
Lane 3: AR KO + G3BP1 overexpressed 1 ug/ml dox treated with 3 days.
Lane 4: AR KO + control, 1 ug/ml dox treated with 0 days
Lane 5 : AR KO + control, 1 ug/ml dox treated with 1 day.
Lane 6: AR KO + control, 1 ug/ml dox treated with 3 days.

Immunoblot images depicted in Supplementary Fig. 5A

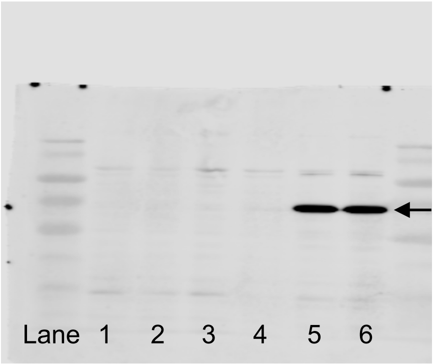

Flag G3BP1

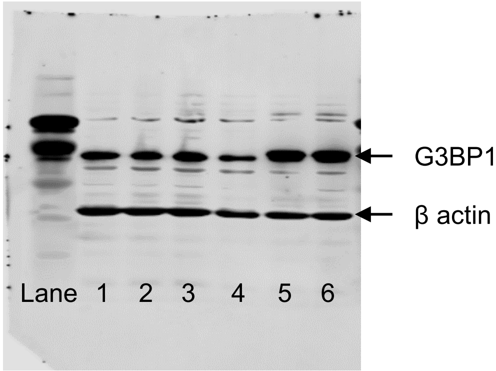

G3BP1

β actin

Lane 1: Cells infected with vector alone, treated with 1 μg/ml DOX for 0 days
Lane 2 : Cells infected with vector alone; treated with 1 μg/ml DOX for 1 day
Lane 3 : Cells infected with vector alone; treated with 1 μg/ml DOX for 3 days
Lane 4 : Cells infected with G3BP1 overexpressed construct ; treated with 1 μg/ml DOX for 3 days
Lane 5 : Cells infected with G3BP1 overexpressed construct ; treated with 1 μg/ml DOX for 1 day
Lane 6: Cells infected with G3BP1 overexpressed construct ; treated with 1 μg/ml DOX for 3 days

Immunoblot images depicted in Supplementary Fig. 5B

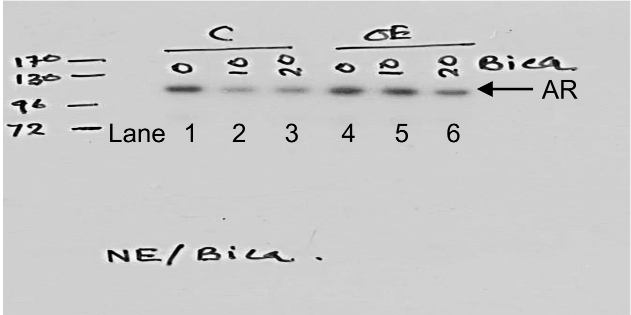

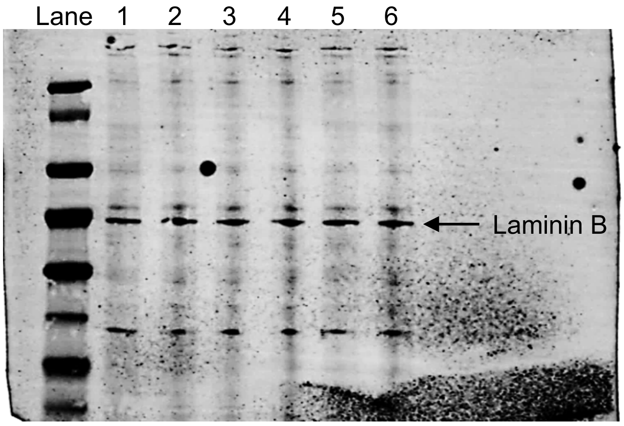

Lane 1: Vector alone, treated with DMSO
Lane 2 : Vector alone; treated with 10μM bicalutamide
Lane 3 : Vector alone; treated with 20μM bicalutamide
Lane 4 : G3BP1 overexpressed cells ; treated with DMSO
Lane 5 : G3BP1 overexpressed cells; treated with 10μM bicalutamide
Lane 6: G3BP1 overexpressed cell ; treated with 20μM bicalutamide

Immunoblot images depicted in Supplementary Fig. 5C

Lane 1 2 3 4 5 6

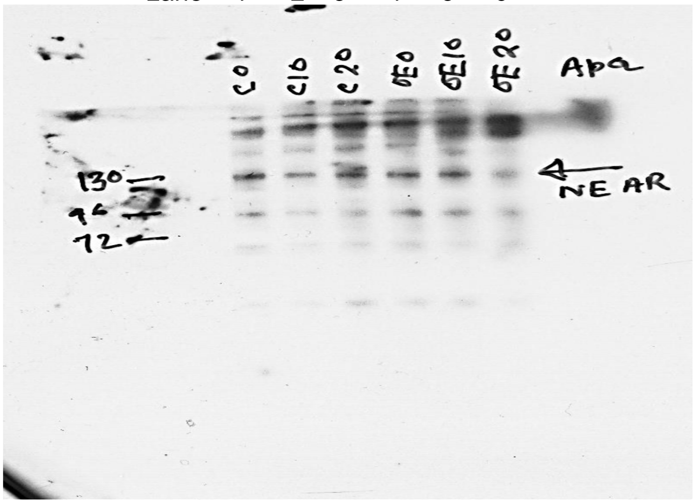

Lane 1 2 3 4 5 6

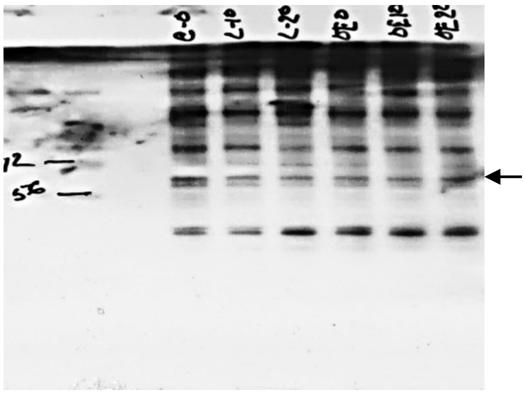

Laminin B

Lane 1: Vector alone, treated with DMSO
Lane 2 : Vector alone; treated with 10μM apalutamide
Lane 3 : Vector alone; treated with 20μM apalutamide
Lane 4 : G3BP1 overexpressed cells ; treated with DMSO
Lane 5 : G3BP1 overexpressed cells; treated with 10μM apalutamide
Lane 6: G3BP1 overexpressed cell ; treated with 20μM apalutamide

Immunoblot images depicted in Supplementary Fig. 5E

C4-2B cell

Lane 1 2 3 4 5 6 7 8

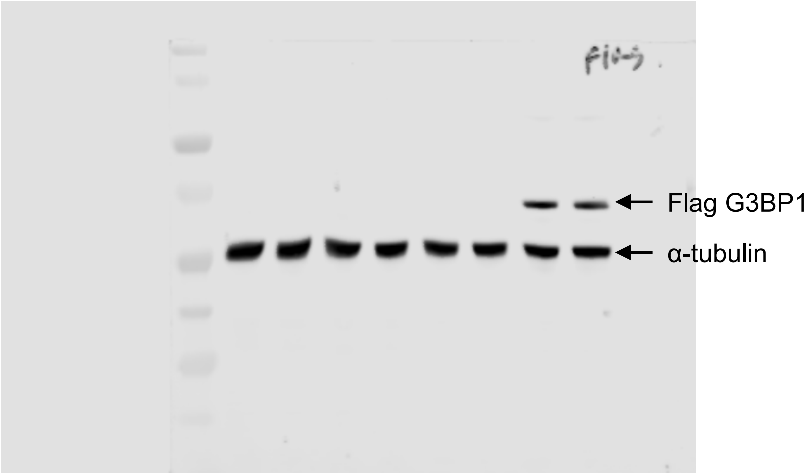

Lane 1: Vector alone, treated with Dox 1 μg/ml for 0 day
Lane 2 : Vector alone; treated with Dox 1 μg/ml for 1 day
Lane 3 : Vector alone; treated with Dox 1 μg/ml for 2 days
Lane 4 : Vector alone; treated with Dox 1 μg/ml for 3 days
Lane 5 : G3BP1 overexpressed cells; treated with Dox 1 μg/ml for 0 day
Lane 6: G3BP1 overexpressed cells; treated with Dox 1 μg/ml for 1 day
Lane 7: G3BP1 overexpressed cells; treated with Dox 1 μg/ml for 2 day
Lane 8: G3BP1 overexpressed cells; treated with Dox 1 μg/ml for 3 day

Immunoblot images depicted in Supplementary Fig. 5F

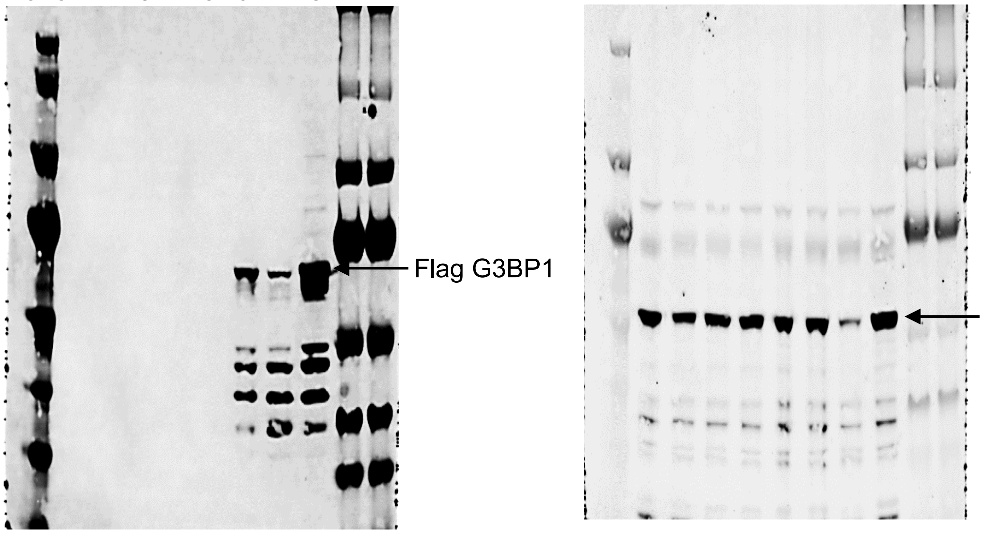

Lane 1: Vector alone, treated with Dox 1 µg/ml for 0 day
Lane 2 : Vector alone; treated with Dox 1 µg/ml for 1 day
Lane 3 : Vector alone; treated with Dox 1 µg/ml for 2 days
Lane 4 : Vector alone; treated with Dox 1 µg/ml for 3 days
Lane 5 : G3BP1 overexpressed cells; treated with Dox 1 µg/ml for 0 day
Lane 6: G3BP1 overexpressed cells; treated with Dox 1 µg/ml for 1 day
Lane 7: G3BP1 overexpressed cells; treated with Dox 1 µg/ml for 2 day
Lane 8: G3BP1 overexpressed cells; treated with Dox 1 µg/ml for 3 day

Immunoblot images depicted in Supplementary Fig. 6C

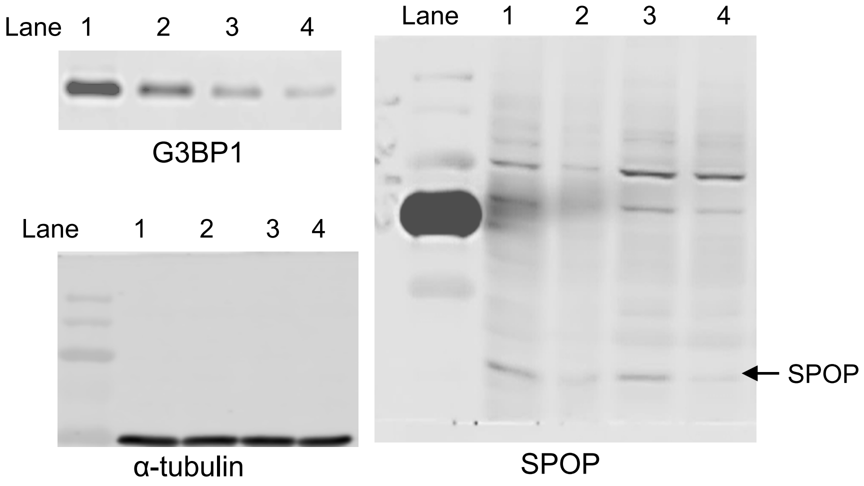

22RV1 cells were transfected with different siRNA
Lane 1: siControl
Lane 2 : siSPOP
Lane 3: siG3BP1
Lane 4: siSPOP+siG3BP1

FACS analysis depicted in Supplementary Fig. 6D

Non-Targeted

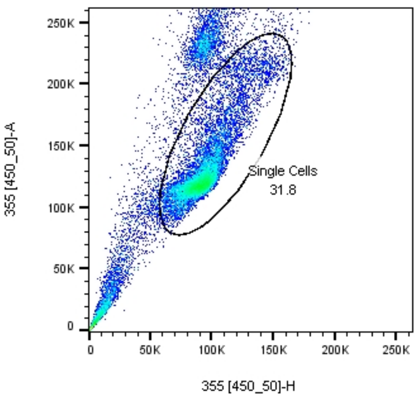

Non-Targeted

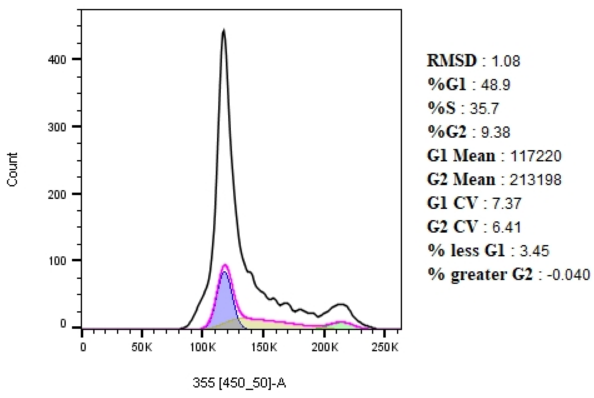

G3BP1 KD

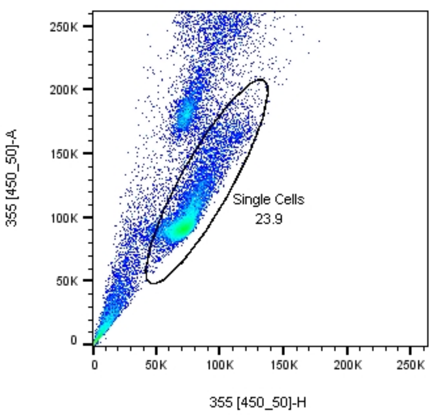

G3BP1 KD

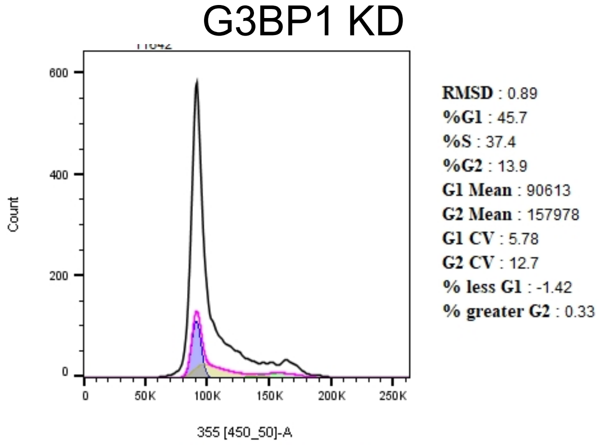

One representative image out of three independent experiments showing FACS sequential gating/sorting strategies used to generate Supplementary Fig. 6D

FACS analysis depicted in Supplementary Fig. 6E

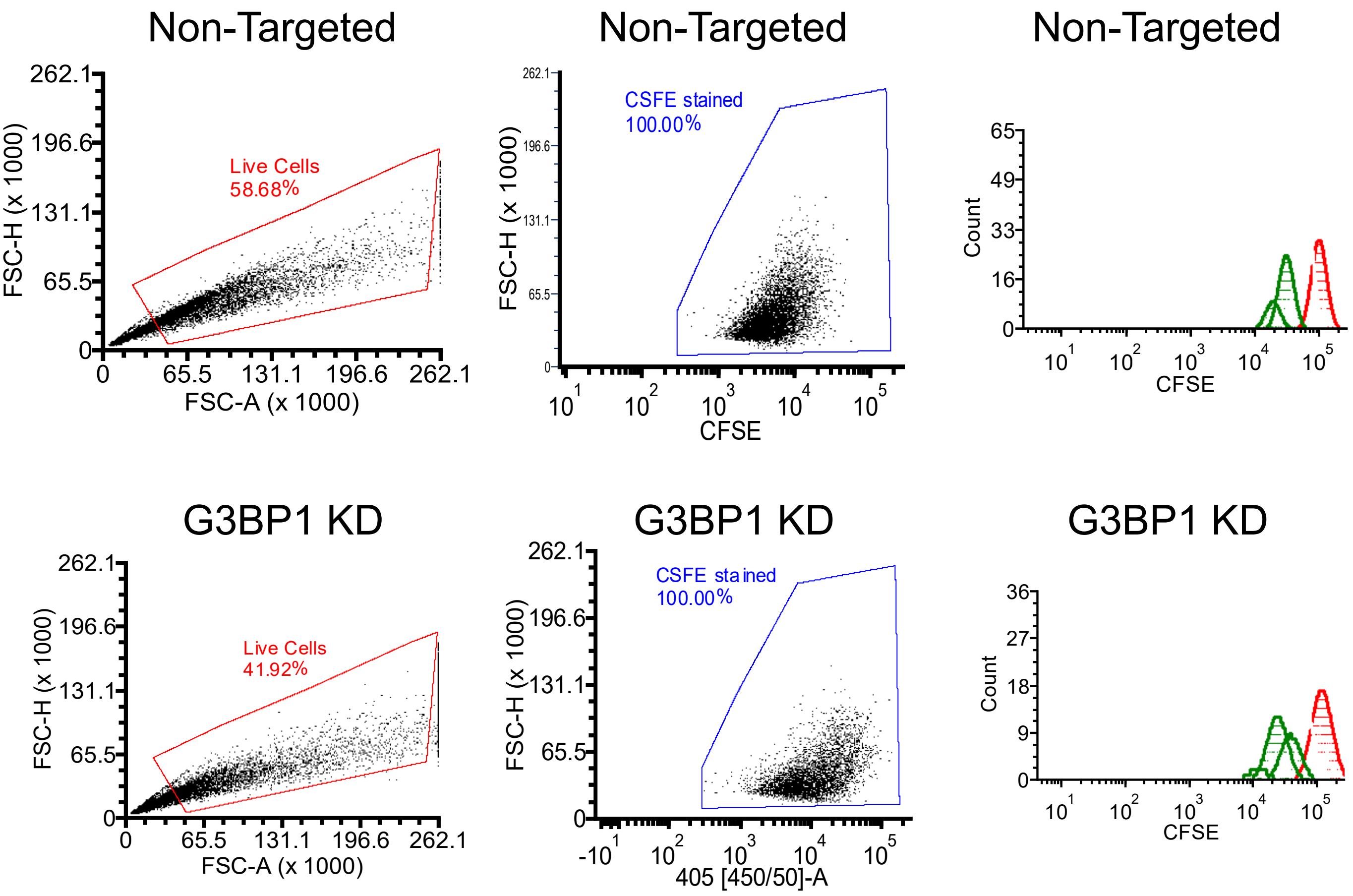

One representative image out of three independent experiments showing FACS sequential gating/sorting strategies used to generate Supplementary Fig. 6E

FACS sequential gating/sorting strategies depicted in Supplementary Fig. 6F

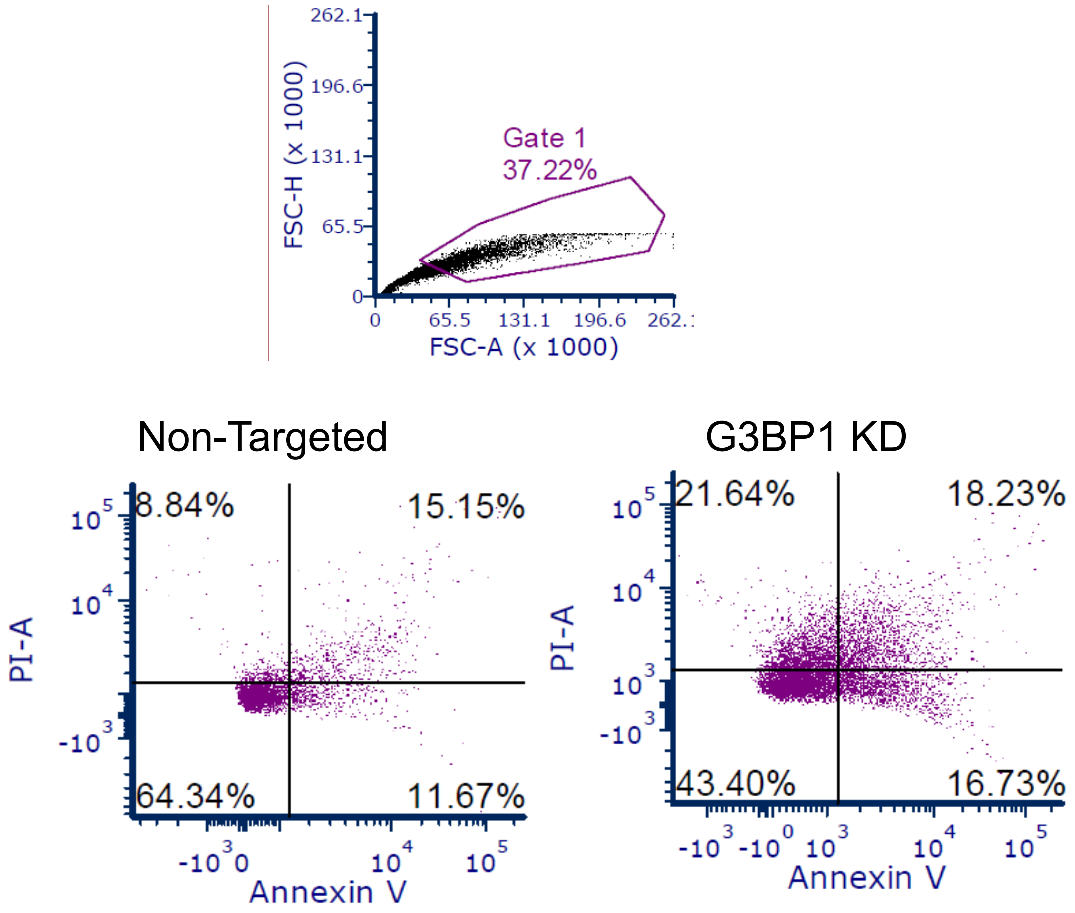

One representative image out of three independent experiments showing FACS sequential gating/sorting strategies used to generate Supplementary Fig. 6F

Immunoblot images depicted in Supplementary Fig. 6G

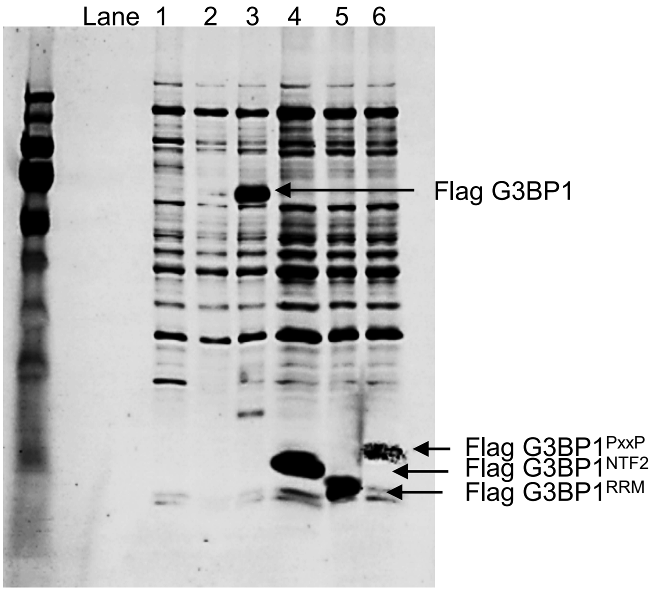

Flag G3BP1

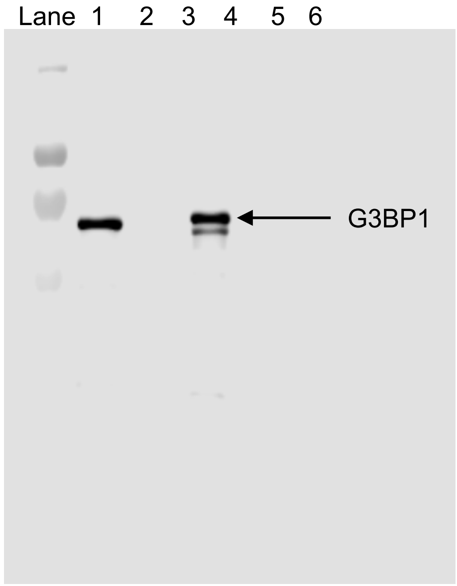

G3BP1

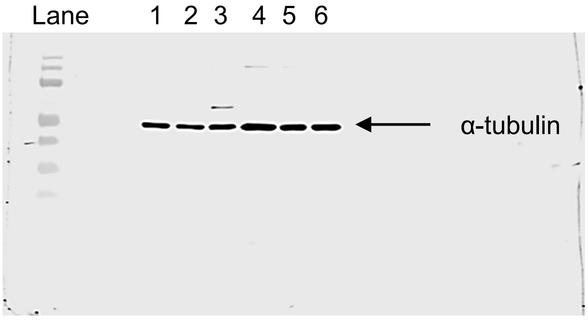

α-tubulin

22RV1 cells were transfected with different plasmid and immunoblot was performed
Lane 1: sgControl
Lane 2 : sgG3BP1
Lane 3: sgG3BP1 + full length G3BP1
Lane 4: sgG3BP1 + G3BP1^{NTF2}
Lane 5: sgG3BP1 + G3BP1^{RRM}
Lane 6: sgG3BP1 + G3BP1^{PxxP}

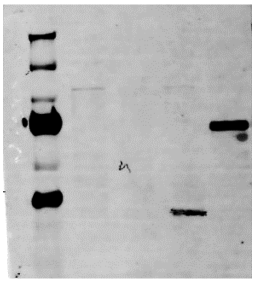

FLAG-Ac-PxxP

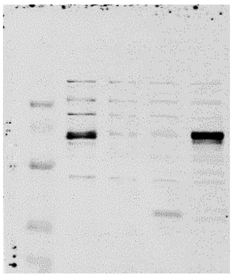

G3BP1

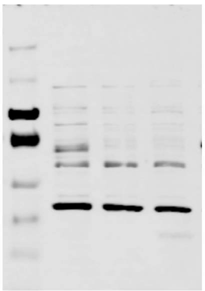

β-actin

22RV1 cells were transfected with different plasmid and immunoblot was performed
Lane 1: sgControl
Lane 2 : sgG3BP1
Lane 3: sgG3BP1 + G3BP1$^{Ac\text{-}PxxP}$

Immunoblot images depicted in Supplementary Fig. 7B

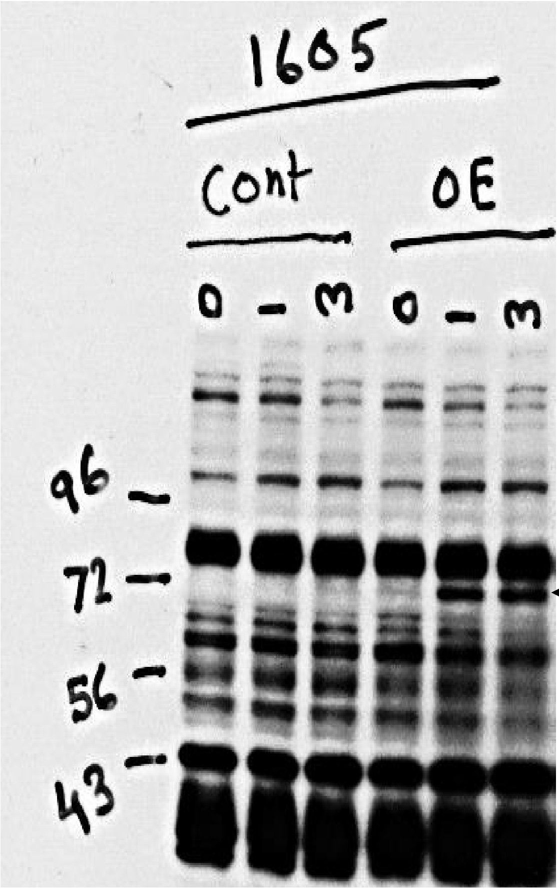

Lane 1 2 3 4 5 6

← Flag G3BP1

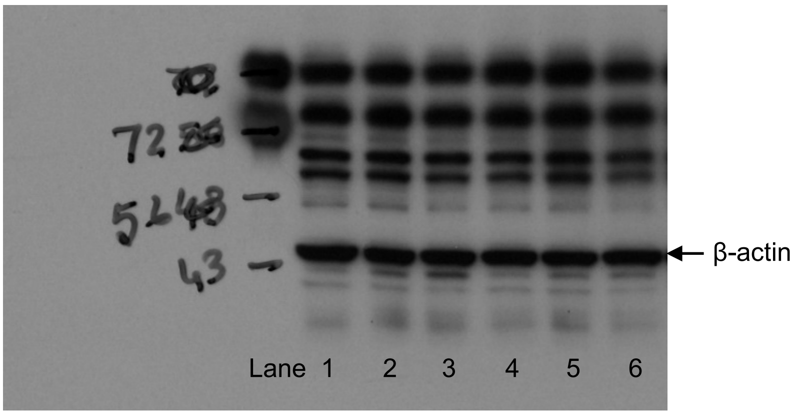

← β-actin

Cell lysates were collected from Pten$^{-/-}$ primary mouse prostate epithelial cell.

Lane 1: Cells infected with vector alone, treated with 1 μg/ml DOX for 0 days

Lane 2: Cells infected with vector alone; treated with 1 μg/ml DOX for 1 day

Lane 3: Cells infected with vector alone; treated with 1 μg/ml DOX for 3 days

Lane 4: Cells infected with G3BP1 overexpressed construct ; treated with 1 μg/ml DOX for 3 days

Lane 5: Cells infected with G3BP1 overexpressed construct ; treated with 1 μg/ml DOX for 1 day

Lane 6: Cells infected with G3BP1 overexpressed construct ; treated with 1 μg/ml DOX for 3 days

Immunoblot images depicted in Supplementary Fig. 7C

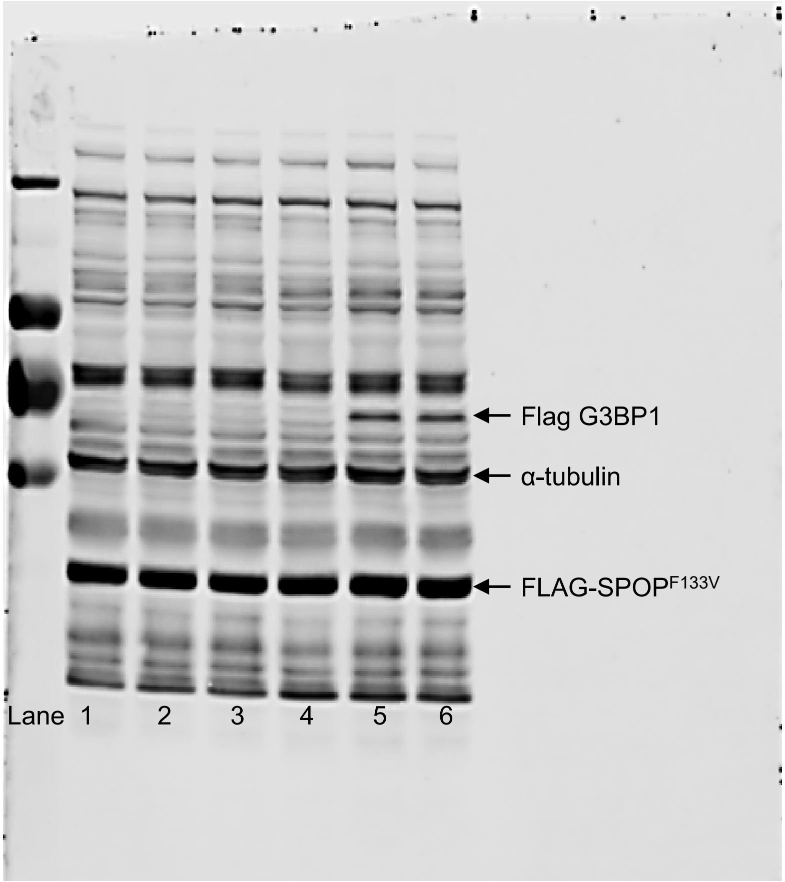

FLAG-G3BP1 andFLAG-SPOP^{F133V} expression

Cell lysates were collected from Pten$^{-/-\ SPOP133V}$primary mouse prostate epithelial cell.
Lane 1: Cells infected with vector alone, 0 µg/ml DOX
Lane 2 : Cells infected with G3BP1 overexpressed construct 0 µg/ml DOX.
Lane 3 : Cells infected with G3BP1 overexpressed construct 0 µg/ml DOX.
Lane 4 : Cells infected with vector alone, 1 µg/ml DOX.
Lane 5 : Cells infected with G3BP1 overexpressed construct ; 1 µg/ml DOX.
Lane 6: Cells infected with G3BP1 overexpressed construct ; 1 µg/ml DOX.

Immunoblot images depicted in Supplementary Fig. 7E

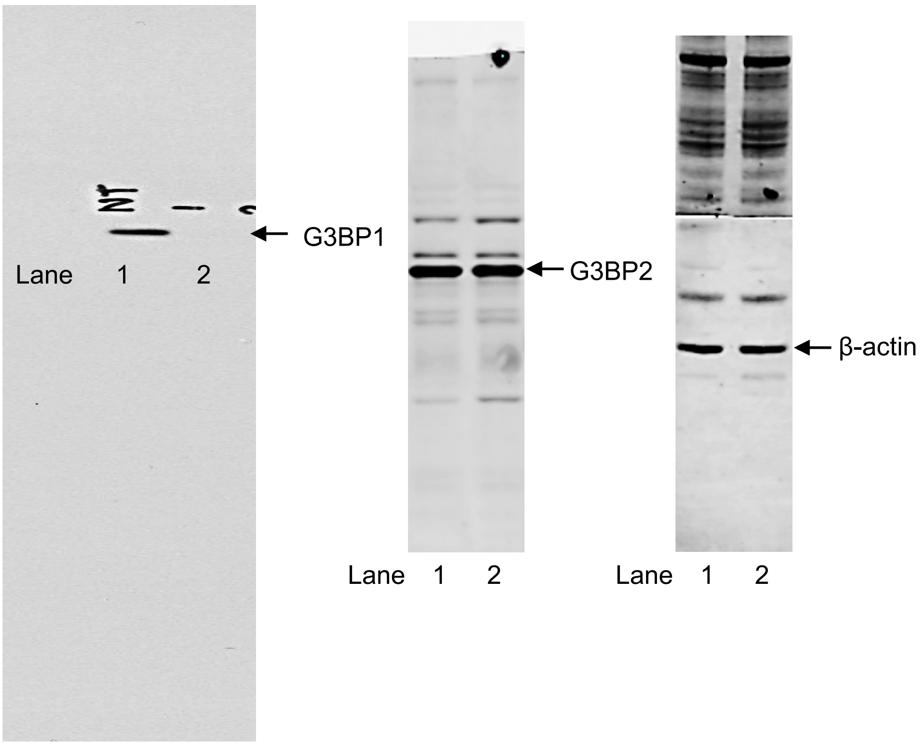

Cell lysates were collected from $Pten^{-/-}$ primary mouse prostate epithelial cell.
Lane 1: Cells infected with NonTarget shRNA
Lane 2 : Cells infected with $G3BP1^{KD}$ shRNA.

Immunoblot images depicted in Supplementary Fig. 7H

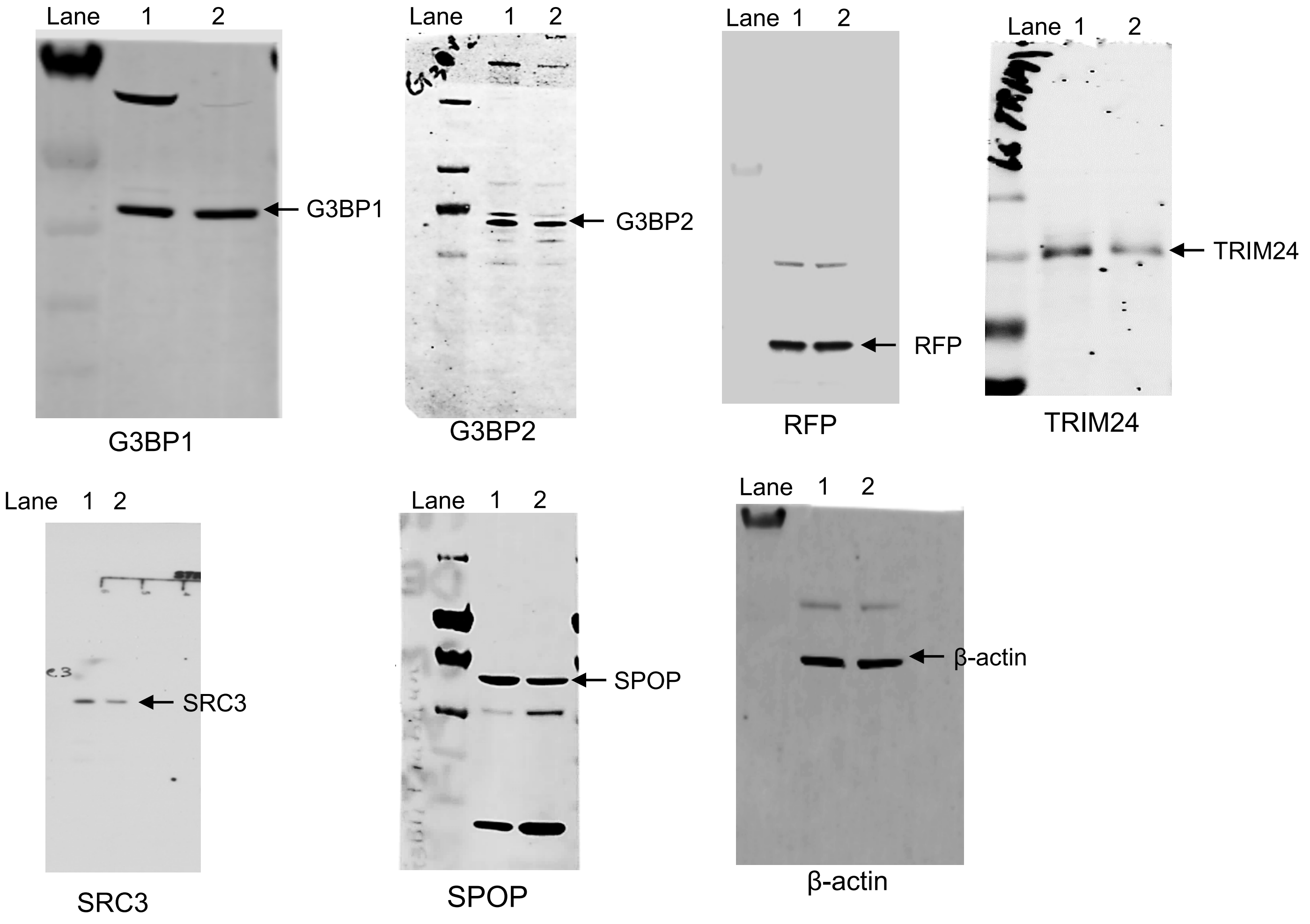

Cell lysates were collected from WT primary mouse prostate epithelial cell.
Lane 1: Cells infected with NonTarget shRNA
Lane 2 : Cells infected with G3BP1^{KD} shRNA.

Immunoblot images depicted in Supplementary Fig. 7I

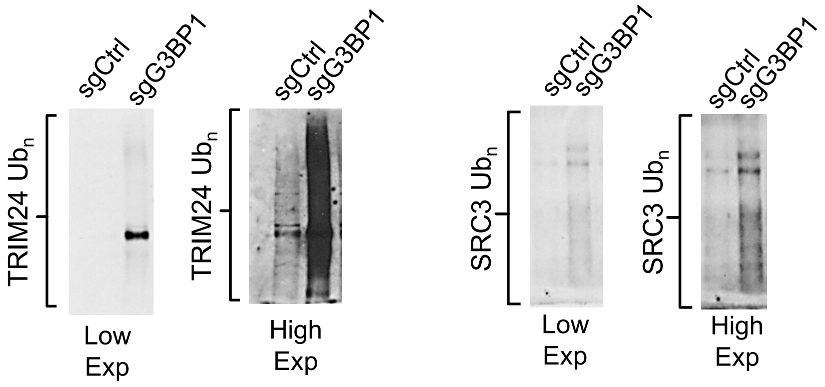

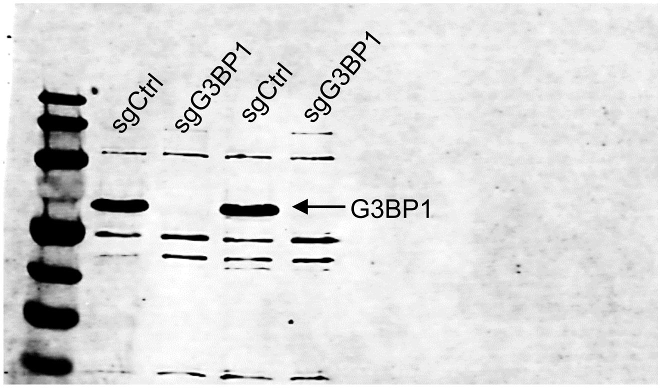

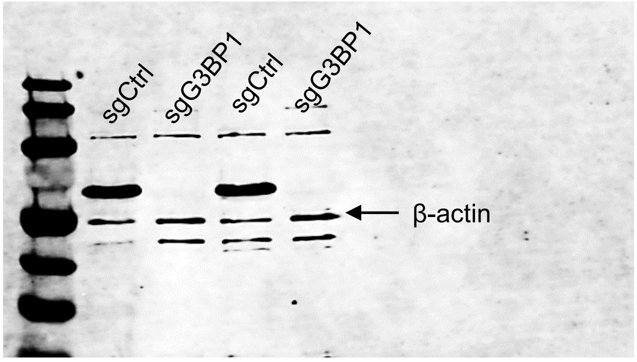

Supplement: Supplementary file 1 — Supplementary Information [file 41467_2021_27024_MOESM1_ESM.pdf]
