# Supplementary material for: G3BP1 inhibits Cul3SPOP to amplify AR signaling and promote prostate cancer
Source: Nat Commun. 2021 Nov 18;12:6662. doi: 10.1038/s41467-021-27024-x (PMC8602290; doi:10.1038/s41467-021-27024-x)
Supplement: Supplementary file 2 — Reporting Summary [file 41467_2021_27024_MOESM2_ESM.pdf]

nature research

Corresponding author(s): Pengbo Zhou

Last updated by author(s): Apr 29, 2021

Reporting Summary

Nature Research wishes to improve the reproducibility of the work that we publish. This form provides structure for consistency and transparency in reporting. For further information on Nature Research policies, see our Editorial Policies and the Editorial Policy Checklist.

Statistics

For all statistical analyses, confirm that the following items are present in the figure legend, table legend, main text, or Methods section.

n/a	Confirmed	
☐	☒	The exact sample size (*n*) for each experimental group/condition, given as a discrete number and unit of measurement
☐	☒	A statement on whether measurements were taken from distinct samples or whether the same sample was measured repeatedly
☐	☒	The statistical test(s) used AND whether they are one- or two-sided<br>*Only common tests should be described solely by name; describe more complex techniques in the Methods section.*
☒	☐	A description of all covariates tested
☒	☐	A description of any assumptions or corrections, such as tests of normality and adjustment for multiple comparisons
☐	☒	A full description of the statistical parameters including central tendency (e.g. means) or other basic estimates (e.g. regression coefficient) AND variation (e.g. standard deviation) or associated estimates of uncertainty (e.g. confidence intervals)
☐	☒	For null hypothesis testing, the test statistic (e.g. *F*, *t*, *r*) with confidence intervals, effect sizes, degrees of freedom and *P* value noted<br>*Give P values as exact values whenever suitable.*
☒	☐	For Bayesian analysis, information on the choice of priors and Markov chain Monte Carlo settings
☒	☐	For hierarchical and complex designs, identification of the appropriate level for tests and full reporting of outcomes
☒	☐	Estimates of effect sizes (e.g. Cohen's *d*, Pearson's *r*), indicating how they were calculated

Our web collection on statistics for biologists contains articles on many of the points above.

Software and code

Policy information about availability of computer code

Data collection

MS spectrum files were transformed into MGF format by MSConvert software v1.0 and interrogated by a MASCOT 2.4 search engine using human UniProt database version 15 concatenated with reverse sequences for estimation of false discovery rate (FDR) and with a list of common contaminants.HiSeq 2500 sequencer (illumina), Brightfield microscope with DP80 camera (Olympus), LSM 880 confocal laser scanning microscope, Promega Glomax luminometer with microplate reader, Roche Light Cycler 480, BioTek Lionheart FX Automated Microscope, Becton-Dickinson Fortessa Analyzer.

Data analysis

MS Data Analysis
MS spectrum files were transformed into MGF format by MSConvert software v1.0 and interrogated by a MASCOT 2.4 search engine using human UniProt database version 15 concatenated with reverse sequences for estimation of false discovery rate (FDR) and with a list of common contaminants. The search parameters were as follows: full tryptic search, 2 allowed missed cleavages, peptide charges +2 and +3 only, MS tolerance 1 Da, MS/MS tolerance 0.5 Da. The only permanent post-translational modification was cysteine carbamidomethylation. Variable post-translational modifications were protein N-terminal acetylation, Met oxidation and N-terminal Glutamine to pyro-Glutamate conversion. The remaining analysis was performed as in [1]. To summarize, the minimal ion score threshold was chosen such that a peptide false discovery rate (FDR) below 1% was achieved. The peptide FDR was calculated as: 2 × (decoy_hits)/ (target + decoy hits). Spectral counts for all detected proteins were assembled using a Python script written in-house. The adjustment of spectral counts was done by the same script as in [1].

Analysis of Human TMAs (protein)
In order to analyze G3BP1 protein expression in a larger number of different prostate cancer patient samples, Weill Cornell Medicine constructed tissue microarrays (TMAs) containing 153 formalin-fixed, paraffin-embedded, prostate cancer tissues. The specimens were collected between 2001 and 2011 in Weill Cornell Medicine, NY, USA. Hematoxylin- and eosin-stained slides of all specimens were re-evaluated by one experienced pathologist (B.D.R.) to identify representative areas. Patients had an average age of 58 years (range: 37-76). The local scientific ethics committees approved the cohort (Protocol # 1007011157). This cohort was used to correlate G3BP1 protein

expression determined by IHC with TRIM24 and AR.

Global Transcriptome Analysis

All analysis of human prostate cancer data was conducted using previously published datasets of The Cancer Genome Atlas (TCGA) cohort [2], Taylor cohort [3], and PCF/SU2C [4], which can be explored in the cBioPortal for Cancer Genomics (http://www.cbioportal.org). The AR output score of different cohorts were calculated by following a strategy similar to that of the TCGA study [2]. Specifically, the AR output score was derived from the mRNA expression of 20 genes that were experimentally validated AR transcriptional targets from the LNCaP cell line [5]. Here the Z-score for the expression of each gene in each sample was calculated and the AR score for each sample was then computed as the sum of the Z-scores of 20 AR signaling genes. Statistics: For comparison of pooled data between two different groups, unpaired t tests were used to determine significance. For Kaplan-Meier analysis of MET-free survival rates, expression profiles of retrospective (n = 1,626) cohorts were derived from the Decipher Genomics Resource Information Database (GRID) registry (ClinicalTrials.gov identifier: NCT02609269). The retrospective GRID cohort was pooled from seven published microarray studies: Cleveland Clinic (CCF) [6], Erasmus MC [7], Johns Hopkins (JHMI) [8], Memorial Sloan Kettering (MSKCC) [9], Mayo Clinic (Mayo I and Mayo II) [10], and Thomas Jefferson University (TJU) [11]. Associated accession numbers are: GSE79957, GSE72291, GSE62667, GSE62116, GSE46691, GSE41408, and GSE21032 [12].

RNA-seq Analysis

22RV1 control, 22RV1 siSPOP, 22RV1 G3BP1 KO and 22RV1 G3BP1 KO plus siSPOP (3 replicates) were prepared for RNA sequencing using TruSeq RNA Library Preparation Kit v2. Each sample was sequenced with the HiSeq 2500 to generate 51 bp paired-end reads. Reads (FASTQ files) were mapped to the human reference genome sequence (hg19/GRCh37 http://hgdownload.cse.ucsc.edu/downloads.html) using STAR v2.4.0j [13], and the resulting BAM files were subsequently converted into mapped-read format (MRF) using RSEQtools v0.6 [14]. The read count of each gene was calculated via HTSeq [15] using GENCODE as the reference gene–annotation set. Quantification of gene expression was performed using RSEQtools with GENCODE as the reference gene–annotation set (http://www.gencodegenes.org/human/release_19.html). Expression levels (RPKM) were estimated by counting all nucleotides mapped to each gene and were normalized by the total number of mapped nucleotides (per million) and the gene length (per kb). Differential expression analyses were performed using DESeq2 (v1.20.0) [16] based on the gene read count data. Multiple-hypothesis testing was considered by using the Benjamini-Hochberg (BH; FDR) correction. Heatmap and hierarchical clustering were performed via using correlation distance and Ward's method. GSEA [17] was performed using the JAVA program and run in pre-ranked mode to identify enriched signatures. We used the gene sets in the Molecular Signature Database (MSigDB) [17]. The GSEA plot, normalized enrichment score and p-values were derived from GSEA output for each MSigDB hallmark signature. The AR output scores of our samples were calculated by following a strategy similar to that of the TCGA study [2]. Specifically, the AR output score was derived from the mRNA expression of 20 genes that were experimentally validated AR transcriptional targets from the LNCaP cell line [5]. Here the Z-score for the expression of each gene in each sample was calculated, and the AR score for each sample was then computed as the sum of the Z-scores of 20 AR signaling genes. By following a similar strategy [18], we developed the G3BP1 overexpression transcriptional signature, which includes 498 overexpressed genes from samples with G3BP1 that was overexpressed compared with wild type samples from TCGA prostate cancer RNA-seq data. Specifically, we identified significantly differentially expressed genes by comparing G3BP1 overexpression and wild type cases as determined from genomic analyses among TCGA samples with ETS family gene fusions (ERG, ETV1, ETV4 and FLI1), using DESeq2 (v1.20.0) [16] and controlled for false discovery using Benjamini-Hochberg adjustment (FDR ≤0.05).

Ki67 Quantification

Three independent, representative images were taken at 20× magnification for each mouse prostate. The percentage of Ki67 positive epithelial cells was calculated for each image and the mean and standard error of all images for each genotype was determined. A Student's 2-sided t-test was used to determine statistical differences between two genotypes.

Tumor-Xenograft Volume Quantification

Volume (mm3) for each xenograft was calculated and plotted. The difference in the mean volume between xenograft lines was determined using a 2-sided Student's t-test.

PLA Quantification

A minimum of 200 cells per condition were quantified in a single plane for detectable foci. The number of foci per cell per condition is reported. Differences in the mean numbers of foci between conditions were determined using a 2-sided Student's t-test.

References:

Organoids number and size were quantified by ImageJ2.

CellTrace Violet cell proliferation assays, Cell cycle analysis and Annexin V-PI staining: Data were analyzed using FCS express version 6 software

1. Poliakov A, Russell CW, Ponnala L, et al. Large-scale label-free quantitative proteomics of the pea aphid-Buchnera symbiosis. Molecular & cellular proteomics : MCP 2011;10(6):M110 007039 doi: 10.1074/mcp.M110.007039[published Online First: Epub Date]|.

2. TCGA. The Molecular Taxonomy of Primary Prostate Cancer. Cell 2015;163(4):1011-25 doi: 10.1016/j.cell.2015.10.025[published Online First: Epub Date]|.

3. Taylor BS, Schultz N, Hieronymus H, et al. Integrative genomic profiling of human prostate cancer. Cancer cell 2010;18(1):11-22 doi: 10.1016/j.ccr.2010.05.026[published Online First: Epub Date]|.

4. Robinson D, Van Allen EM, Wu YM, et al. Integrative clinical genomics of advanced prostate cancer. Cell 2015;161(5):1215-28 doi: 10.1016/j.cell.2015.05.001[published Online First: Epub Date]|.

5. Hieronymus H, Lamb J, Ross KN, et al. Gene expression signature-based chemical genomic prediction identifies a novel class of HSP90 pathway modulators. Cancer cell 2006;10(4):321-30 doi: 10.1016/j.ccr.2006.09.005[published Online First: Epub Date]|.

6. Klein EA, Yousefi K, Haddad Z, et al. A genomic classifier improves prediction of metastatic disease within 5 years after surgery in node-negative high-risk prostate cancer patients managed by radical prostatectomy without adjuvant therapy. European urology 2015;67(4):778-86 doi: 10.1016/j.eururo.2014.10.036[published Online First: Epub Date]|.

7. Boormans JL, Korsten H, Ziel-van der Made AJ, et al. Identification of TDRD1 as a direct target gene of ERG in primary prostate cancer. International journal of cancer 2013;133(2):335-45 doi: 10.1002/ijc.28025[published Online First: Epub Date]|.

8. Ross AE, Johnson MH, Yousefi K, et al. Tissue-based Genomics Augments Post-prostatectomy Risk Stratification in a Natural History Cohort of Intermediate- and High-Risk Men. European urology 2016;69(1):157-65 doi: 10.1016/j.eururo.2015.05.042[published Online First: Epub Date]|.

9. Erho N, Crisan A, Vergara IA, et al. Discovery and validation of a prostate cancer genomic classifier that predicts early metastasis following radical prostatectomy. PloS one 2013;8(6):e66855 doi: 10.1371/journal.pone.0066855[published Online First: Epub Date]|.

10. Karnes RJ, Bergstralh EJ, Davicioni E, et al. Validation of a genomic classifier that predicts metastasis following radical prostatectomy in an

at risk patient population. The Journal of urology 2013;190(6):2047-53 doi: 10.1016/j.juro.2013.06.017[published Online First: Epub Date]|.
11. Den RB, Feng FY, Showalter TN, et al. Genomic prostate cancer classifier predicts biochemical failure and metastases in patients after postoperative radiation therapy. International journal of radiation oncology, biology, physics 2014;89(5):1038-46 doi: 10.1016/j.ijrobp.2014.04.052[published Online First: Epub Date]|.
12. Liu D, Takhar M, Alshalalfa M, et al. Impact of the SPOP Mutant Subtype on the Interpretation of Clinical Parameters in Prostate Cancer. JCO precision oncology 2018;2018 doi: 10.1200/PO.18.00036[published Online First: Epub Date]|.
13. Dobin A, Davis CA, Schlesinger F, et al. STAR: ultrafast universal RNA-seq aligner. Bioinformatics 2013;29(1):15-21 doi: 10.1093/bioinformatics/bts635[published Online First: Epub Date]|.
14. Habegger L, Sboner A, Gianoulis TA, et al. RSEQtools: a modular framework to analyze RNA-Seq data using compact, anonymized data summaries. Bioinformatics 2011;27(2):281-3 doi: 10.1093/bioinformatics/btq643[published Online First: Epub Date]|.
15. Anders S, Pyl PT, Huber W. HTSeq--a Python framework to work with high-throughput sequencing data. Bioinformatics 2015;31(2):166-9 doi: 10.1093/bioinformatics/btu638[published Online First: Epub Date]|.
16. Love MI, Huber W, Anders S. Moderated estimation of fold change and dispersion for RNA-seq data with DESeq2. Genome biology 2014;15(12):550 doi: 10.1186/s13059-014-0550-8[published Online First: Epub Date]|.
17. Subramanian A, Tamayo P, Mootha VK, et al. Gene set enrichment analysis: a knowledge-based approach for interpreting genome-wide expression profiles. Proceedings of the National Academy of Sciences of the United States of America 2005;102(43):15545-50 doi: 10.1073/pnas.0506580102[published Online First: Epub Date]|.
18. Blattner M, Liu D, Robinson BD, et al. SPOP Mutation Drives Prostate Tumorigenesis In Vivo through Coordinate Regulation of PI3K/mTOR and AR Signaling. Cancer cell 2017;31(3):436-51 doi: 10.1016/j.ccell.2017.02.004[published Online First: Epub Date]|.
19. Lee J, Shieh JH, Zhang J, et al. Improved ex vivo expansion of adult hematopoietic stem cells by overcoming CUL4-mediated degradation of HOXB4. Blood 2013;121(20):4082-9 doi: 10.1182/blood-2012-09-455204[published Online First: Epub Date]|.

For manuscripts utilizing custom algorithms or software that are central to the research but not yet described in published literature, software must be made available to editors and reviewers. We strongly encourage code deposition in a community repository (e.g. GitHub). See the Nature Research guidelines for submitting code & software for further information.

Data

Policy information about availability of data

All manuscripts must include a data availability statement. This statement should provide the following information, where applicable:

- Accession codes, unique identifiers, or web links for publicly available datasets
- A list of figures that have associated raw data
- A description of any restrictions on data availability

RNA-Seq data has been deposited in GEO under the accession GSE138612. https://www.ncbi.nlm.nih.gov/geo/query/acc.cgi?acc=GSE138612
Original/source data for Figure 3A in the paper is available in Cell 163, 1011-1025 (2015).
Original/source data for Figure 3B in the paper is available in Decipher; GenomeDx Biosciences, Vancouver, BC, Canada. Nature,571,408-412(2019)
Original/source data for Figures 4G in the paper is available in Cell 163, 1011-1025 (2015), Cancer cell 18, 11-22, Cell 161, 1215-1228.
All data supporting the findings of this study are available within the article and its Supplementary information files and from the corresponding author upon reasonable request. Source data are provided with this paper. The data that support the finding in Supplementary Fig. 1A of this study is deposited in proteomeXchange accession no. PXD029120.

Field-specific reporting

Please select the one below that is the best fit for your research. If you are not sure, read the appropriate sections before making your selection.

☒ Life sciences ☐ Behavioural & social sciences ☐ Ecological, evolutionary & environmental sciences

For a reference copy of the document with all sections, see nature.com/documents/nr-reporting-summary-flat.pdf

Life sciences study design

All studies must disclose on these points even when the disclosure is negative.

Sample size	All sample sizes are included in the relevant figure legends.
Data exclusions	No exclusions
Replication	All experiments were repeated at least for three times. Detailed information on replicates are available in the figure legends. All attempts to replicate the experiments performed here were successful.
Randomization	No method of randomization was used. Male littermates were used for mouse experiments. Biological replicates of sequencing-based assays were performed in separate experiments at different times to control for co-variates. Randomization is not applicable to cell culture experiments.
Blinding	Data acquisition in the studies, including animal experiments, was conducted in a blinded manner. Briefly, large cohorts of mice were injected with the indicated cell lines and at a given timepoints tumor volumes were measured without knowing the cell type injected. For the other experiments, samples were all processed in parallel in a blind fashion by assigning numerical IDs to the samples.

Reporting for specific materials, systems and methods

We require information from authors about some types of materials, experimental systems and methods used in many studies. Here, indicate whether each material, system or method listed is relevant to your study. If you are not sure if a list item applies to your research, read the appropriate section before selecting a response.

Materials & experimental systems

n/a	Involved in the study
☐	☒ Antibodies
☐	☒ Eukaryotic cell lines
☒	☐ Palaeontology and archaeology
☐	☒ Animals and other organisms
☐	☒ Human research participants
☒	☐ Clinical data
☒	☐ Dual use research of concern

Methods

n/a	Involved in the study
☒	☐ ChIP-seq
☐	☒ Flow cytometry
☒	☐ MRI-based neuroimaging

Antibodies

Antibodies used

HA (BioLegend, Cat# 901502) used at 1:2000 for immunoprecipitation and immunoblot analysis, Myc (Cell Signaling Cat# 2278S) used at 1:1000 for immunoprecipitation and immunoblot analysis, FLAG (Sigma, Cat# F1804-1) used at 1:2000 /1:1000 for immunoblot, immunoprecipitation and immunofluorescence assay. His (Cell Signaling, Cat# 2365S) used at 1:2000 for immunoblot, immunoprecipitation assays. Cul3 (Cell Signaling, Cat# 2759) used at 1:1000, for immunoblot, analysis. G3BP1 (Protein tech, Cat# I3057-2-AP) used at 1:1000 for immunoblot, immunoprecipitation studies. G3BP1 (Abcam Cat# 56574) used at 1:1000/1:4000 for immunoblot and PLA assay. SPOP antibody, 1:1000 (Protein TechCat# 16750-1-AP) used for immunoblot analysis. SRC3 (Cell signaling, Cat# 2126S) used at 1:500 for immunoblot analysis. AR (Abcam, Cat# 133273) used at 1:1000 for immunoblot analysis. AR (Santa Cruz Cat# c-816) used at 1:500 for immunoblot analysis. TRIM24 (Santa Cruz, Cat# sc-271266) used at 1:500 for immunoblot analysis. α-tubulin (Protein Tech, Cat# 6603I-I-ig) used at 1:5000 for immunoblot analysis. Laminin B (Santa Cruz, Cat# sc-56143) used at 1:500 for immunoblot analysis. β-actin (Protein Tech, Cat# 20536-I-AP) used at 1:5000 for immunoblot analysis. G3BP2 (Abcam Cat# ab86135) used at 1:1000 for immunoblot analysis. Mouse IgG (BioLegend Cat# 400102) used in immunoprecipitation at 1μg/reaction. Rabbit IgG (Abcam, Cat# b37415) used in immunoprecipitation at 1μg/reaction. Ki67 (Bethyl, Cat# A700-021) used at 1:1000 for IHC. E cadherin (BD Bioscience, Cat# 610405) used at 1:2000 for immunofluorescence study. Cytokeratin 5 (Biolegend, Cat# 905501) used at 1:5000 for immunofluorescence study. Cytokeratin 8 (Progen, Cat# 61038S) used at 1:2000 for immunofluorescence study. RFP (Abcam, Cat# ab62341) used at 1:1000 for immunoblot analysis. Cleaved PARP (Abcam Cat # ab32064) used at 1:1000 for immunoblot analysis. PSA (KLK3) [Sigma-Aldrich, Cat# SAB1410703] used at 1:1000 for immunoblot analysis. TMPRSS2 (Abcam, Cat# ab92323) used at 1:1000 for immunoblot analysis. FKBP5 (Abcam, Cat# ab126715) , used at 1:1000 for immunoblot analysis. Secondary antibodies from LI-COR used for immunoblot analysis were (1) Mouse 800CW (Cat#925-32210) (2) Mouse 680RD (Cat#926-68070) 3) Rabbit 800CW (Cat#926-32211) (4) Rabbit 680RD (Cat#926-68071) at 1:20,000 dilution.

Secondary antibodies from Thermo Fisher Scientific used for Immunofluorescence analysis were (1) Alexa fluor 488 Mouse (Cat# A32723) (2) Alexa fluor 594 Mouse (Cat# A32742) 3) Alexa fluor 488 Rabbit (Cat# A32731) (4) Alexa fluor 594 Rabbit (Cat# A32740) at 1:500 dilution.

Validation

All antibodies are validated by the manufacturer and information can be found at the following websites: https://www.biolegend.com/en-us/products/purified-anti-ha-11-epitope-tag-antibody-11374, https://www.cellsignal.com/products/primary-antibodies/myc-tag-71d10-rabbit-mab/2278?site-search-type=Products&N=4294956287&Ntt=2278s&fromPage=plp&_requestid=539539, https://www.sigmaaldrich.com/US/en/substance/monoclonalantiflagm2antibodyproducedinmouse1234598765?context=product, https://www.cellsignal.com/products/primary-antibodies/his-tag-antibody/2365?site-search-type=Products&N=4294956287&Ntt=+2365s&fromPage=plp&_requestid=539937, https://www.cellsignal.com/products/primary-antibodies/cul3-antibody/2759?site-search-type=Products&N=4294956287&Ntt=+2759&fromPage=plp&_requestid=539976, https://www.ptglab.com/products/G3BP1-Antibody-13057-2-AP.htm, https://www.abcam.com/g3bp-antibody-2f3-ab56574.html, https://www.cellsignal.com/products/primary-antibodies/src-3-5e11-rabbit-mab/2126?site-search-type=Products&N=4294956287&Ntt=2126s&fromPage=plp&_requestid=540258, https://www.abcam.com/androgen-receptor-antibody-epr15352-ab133273.html, https://www.scbt.com/p/ar-antibody-n-20?requestFrom=search, https://www.scbt.com/p/tif1alpha-antibody-c-4?requestFrom=search, https://www.ptglab.com/products/tubulin-Alpha-Antibody-66031-1-Ig.htm, https://www.scbt.com/p/lamin-b1-antibody-119d5-f1?requestFrom=search, https://www.ptglab.com/products/ACTB-Antibody-20536-1-AP.htm, https://www.abcam.com/g3bp2-antibody-ab86135.html, https://www.biolegend.com/en-us/products/purified-mouse-igg1-kappa-isotype-ctrl-1375, https://www.abcam.com/rabbit-igg-polyclonal-isotype-control-ab37415.html, https://www.bethyl.com/product/A700-021?referrer=search, https://www.bdbiosciences.com/en-us/products/reagents/microscopy-imaging-reagents/immunofluorescence-reagents/purified-mouse-anti-e-cadherin.610405, https://www.biolegend.com/en-us/products/keratin-5-polyclonal-antibody-purified-10956, https://www.progen.com/anti-Keratin-K8-mouse-monoclonal-Ks8.7-purified-sample-ready-to-use/61038S, https://www.abcam.com/rfp-antibody-ab62341.html, https://www.abcam.com/cleaved-parp1-antibody-e51-ab32064.html, https://www.sigmaaldrich.com/US/en/search/sab1410703?focus=products&page=1&perPage=30&sort=relevance&term=SAB1410703&type=product, https://www.abcam.com/tmprss2-antibody-epr3861-ab92323.html, https://www.abcam.com/fkbp51-antibody-epr6617-ab126715.html, https://www.licor.com/bio/reagents/irdye-800cw-goat-anti-mouse-igg-secondary-antibody, https://www.licor.com/bio/reagents/irdye-680rd-goat-anti-mouse-igg-secondary-antibody, https://www.licor.com/bio/reagents/irdye-800cw-goat-anti-rabbit-igg-secondary-antibody, https://www.licor.com/bio/reagents/irdye-680rd-goat-anti-rabbit-igg-secondary-antibody, https://www.thermofisher.com/antibody/product/Goat-anti-Mouse-IgG-H-L-Highly-Cross-Adsorbed-Secondary-Antibody-Polyclonal/A32723, https://www.thermofisher.com/antibody/product/Goat-anti-Mouse-IgG-H-L-Highly-Cross-Adsorbed-Secondary-Antibody-Polyclonal/A32742, https://

www.thermofisher.com/antibody/product/Goat-anti-Rabbit-IgG-H-L-Highly-Cross-Adsorbed-Secondary-Antibody-Polyclonal/A32731, https://www.thermofisher.com/antibody/product/Goat-anti-Rabbit-IgG-H-L-Highly-Cross-Adsorbed-Secondary-Antibody-Polyclonal/A32740, https://www.ptglab.com/products/SPOP-Antibody-16750-1-AP

Eukaryotic cell lines

Policy information about cell lines

Cell line source(s)	Cell line source(s):<br>Cell Lines<br>HEK 293T, LNCaP, C4-2B and 22RV1 cells were purchased from the American Type Culture Collection (ATCC- Item # ACS-4500, CRL-1740, CRL-3315, CRL-2505). LNCaP-95 were kindly provided by Dr. Loda's laboratory (WCMC, NY, US) (RRID:CVCL_ZC87). Primary WT mPECs (mouse prostate epithelial cells), Pten-/- mPECs and Pten-/--SPOPF133V mPECs were generated in Dr. Barbieri's laboratory (WCMC, NY, US).<br><br>CRISPR Model Generation<br>LNCaP and 22RV1 cells (<passage 10) were transfected with All-In-One pLentiCRISPR v2/sgRNA plasmids containing either control or G3BP1 specific sgRNAs (AACGTTTGTCCTTGCTCCTG) purchased from GenScript. Cells were selected with puromycin until resistant populations emerged, and then assayed for G3BP1 depletion using immunoblot.<br><br>Engineered G3BP1 overexpression model<br>Stable pooled populations of 22RV1, LNCaP-95, C4-2B control and G3BP1 overexpressed [pCW57.1-FLAG G3BP1 (overexpressed)]. cells were generated by selection using 3 µg/mL puromycin. Levels of mRNA transcripts, expression of protein, and the phenotype of cells were analyzed.<br><br>Engineered Mouse Prostate Epithelial Cells<br>Primary mouse (C57BL/6) prostate epithelial cells were isolated by dissecting 6-10-week-old WT, Pten-/-, and Pten-/-SPOPF133V mouse prostates. Cells were infected with SMARTvector inducible plasmid either with non-targeted control or G3BP1 specific shRNA (ATTCCGAGACACCAAACGC) purchased from Dharmacon. ARKO (knockout) cells were generously provided by Dr. Barbieri. For overexpression, WT, Pten-/-, Pten-/- SPOPF133V, and ARKO cells were infected with DOX-inducible empty vector (control) and pCW57.1-FLAG G3BP1 (overexpressed) lenti-virus. Cells were selected with puromycin until resistant populations emerged, and then treated with 1 µg/mL DOX for 72 h followed by RFP positive cell sorting. G3BP1 stable knockdown or overexpression was confirmed by immunoblot analysis.<br><br>Transient Models<br>22RV1 cells were transfected with either non-targeted control, ON-TARGETplus smart pool or G3BP1 siRNA (Dharmacon Cat# D-001810-10-05, L-012099-00-0005) and siGenome smartpools to target SPOP (M-017919-02-0005), using Lipofectamine RNAiMAX (Thermo Fisher Scientific Cat# 13778150). Twenty-four hours post-transfection, cells were plated onto migration and invasion chambers (corning Cat# 3462 and 354480) and assessed for migratory and invasive cells.
Authentication	All cells were authenticated using short tandem repeat profiling performed by ATCC.
Mycoplasma contamination	All cultured cells were Confirmed for free of mycoplasma monthly via the highly sensitive PCR-based kit from Sigma (Cat # MP0025).
Commonly misidentified lines<br>(See ICLAC register)	No commonly misidentified cell lines were used.

Animals and other organisms

Policy information about studies involving animals; ARRIVE guidelines recommended for reporting animal research

Laboratory animals	All procedures involving mice were approved by the Institutional Animal Care and Use Committee (approval number 2017-0008) at Weill Cornell Medicine and complied with all relevant ethical regulations. Mice were maintained in micro-isolators in the USDA (U.S. Department of Agriculture)/AALAC (American Association of Laboratory Animal Science) accredited facility at the Weill Cornell Medicine on a 12 h light, 12 h dark cycle. The mice were allowed food and acidified water ad libitum.In this study were used 3–4-week-old male NU/J mice (Jackson Laboratories, Bar Harbor, Maine).
Wild animals	No wild animals were used in this study.
Field-collected samples	No field-collected samples were used in this study.
Ethics oversight	All procedures involving mice were approved by the Institutional Animal Care and Use Committee (approval number 2017-0008) at Weill Cornell Medicine and complied with all relevant ethical regulations.

Note that full information on the approval of the study protocol must also be provided in the manuscript.

Human research participants

Policy information about studies involving human research participants

Population characteristics	The specimens were collected between 2001 and 2011 in Weill Cornell Medicine, NY, USA. Tumor specimens were obtained prospectively through Institutional Review Board (IRB)-approved protocol with informed consent (WCM IRB no. Protocol # 1007011157). For these studies, 60 benign prostate tissues, 73 localized prostate cancers, 16 metastatic castration-resistant prostate adenocarcinomas. All patients were male and tumors were classified based on histomorphology using a published pathologic classification system [Epstein et al., Am. J. Surg. Pathol. 38,756-767 (2014)]. No other covariates were assessed
Recruitment	We used available samples for this study. There is no self-selection bias or other biases are present. Male patients had an average age of 58 years (range: 37-76).
Ethics oversight	The local scientific ethics committees and Weill Cornell Medicine approved the protocol (Protocol # 1007011157) and the cohort. This cohort was used to correlate G3BP1 protein expression determined by IHC with TRIM24 and AR.

Note that full information on the approval of the study protocol must also be provided in the manuscript.

Flow Cytometry

Plots

Confirm that:

- [x] The axis labels state the marker and fluorochrome used (e.g. CD4-FITC).
- [x] The axis scales are clearly visible. Include numbers along axes only for bottom left plot of group (a 'group' is an analysis of identical markers).
- [x] All plots are contour plots with outliers or pseudocolor plots.
- [x] A numerical value for number of cells or percentage (with statistics) is provided.

Methodology

Sample preparation	CFSE dye dilution and CellTrace Violet cell proliferation assays: 22RV1shCtrl and 22RV1shG3BP1 (500,000 cells/ml) were stained with CFSE or CellTrace Violet according to the manufacturer's instructions for 48 h. Dilution of CFSE or CellTrace Violet fluorescence as an indicator of cell division was assessed using FACS analysis. Data were analyzed using FCS express 6 software to delineate the percentage of cells that had undergone increasing number of divisions to determine the proliferation index.<br>Cell Cycle Analysis: Cell cycle analysis was performed by flow cytometry using standard procedures as described previously [19]. Briefly, 22RV1shCtrl and 22RV1shG3BP1 (2*106/ml) cells were fixed with 70% ethanol followed by treatment with RNase A for 10 min and then stained with DAPI (DAPI 1μg/mL, Triton X100 0.1%) for 30 min in the dark. Analysis was performed on a flow cytometer Fortessa and data analysis was conducted using FCS express 6 software. All samples were analyzed in triplicate, and the data presented are the average of three independent experiments.<br>Annexin V-PI staining: Occurrence of apoptosis was measured using the Annexin V-FITC Apoptosis Detection Kit according to the manufacturer's protocol. Briefly, 22RV1shCtrl and 22RV1shG3BP1cells were suspended with binding buffer and incubated with Annexin-V-FITC and propidium iodide in binding buffer for 10min at room temperature. Analysis was performed on a flow cytometer Fortessa and data analysis was conducted using FCS express 6 software. All samples were analyzed in triplicate, and the data presented are the average of the three independent experiments.<br>Reference:<br>19. Lee J, Shieh JH, Zhang J, et al. Improved ex vivo expansion of adult hematopoietic stem cells by overcoming CUL4-mediated degradation of HOXB4. Blood 2013;121(20):4082-9 doi: 10.1182/blood-2012-09-455204[published Online First: Epub Date]|.
Instrument	Data were acquired on an LSRII (BD Biosciences).
Software	FCS express 6 software was used to analyzed data
Cell population abundance	Post sort RFP positive cells were analyzed by immunofluorescence and immunoblot analysis.
Gating strategy	Forward vs side scatter (FSC vs SSC) gating was used to identify the cell of interest. Exclude debris and dead cells. Unstained cells were used as a negative control.

- [x] Tick this box to confirm that a figure exemplifying the gating strategy is provided in the Supplementary Information.
