# Supplementary material for: G3BP1 inhibits Cul3SPOP to amplify AR signaling and promote prostate cancer
Source: Nat Commun. 2021 Nov 18;12:6662. doi: 10.1038/s41467-021-27024-x (PMC8602290; doi:10.1038/s41467-021-27024-x)
Supplement: Supplementary file 3 — Source Data [file 41467_2021_27024_MOESM3_ESM.zip › 8-Sourse data/Uncropped gel images.pdf]

Immunoblot images depicted in Fig. 1a

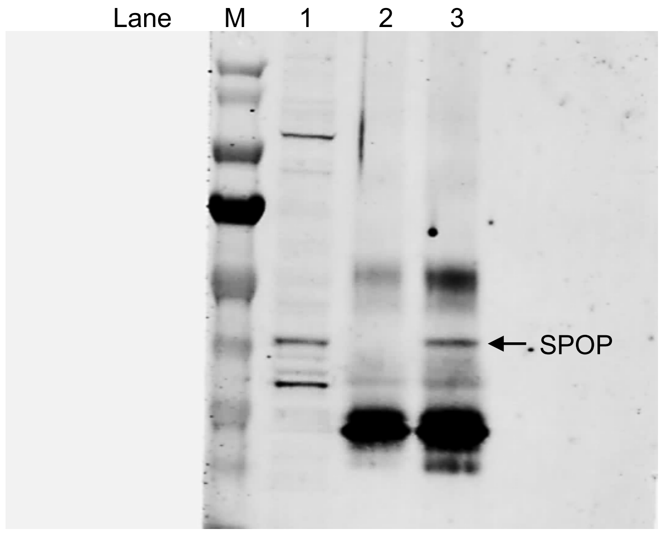

Lane 1: Marker
Lane 2: Whole cell lysate
Lane 3: IgG control
Lane 4: Pull down with endogenous G3BP1 and immunoblotted with endogenous SPOP

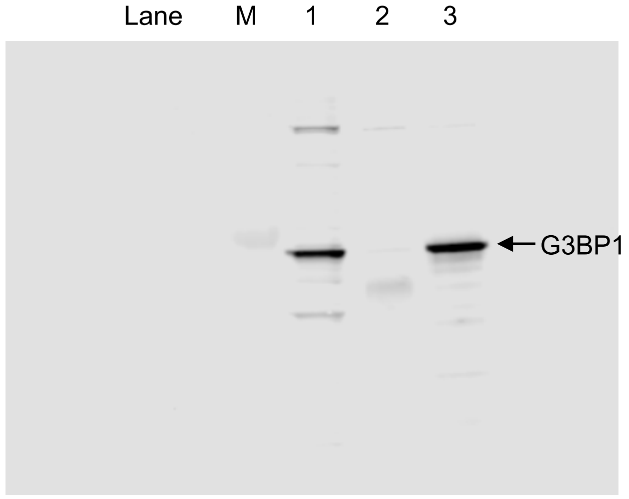

Lane 1: Marker
Lane 2: Whole cell lysate
Lane 3: IgG control
Lane 4: Pull down with endogenous G3BP1 and immunoblotted with endogenous G3BP1

Immunoblot images depicted in Figure 1B

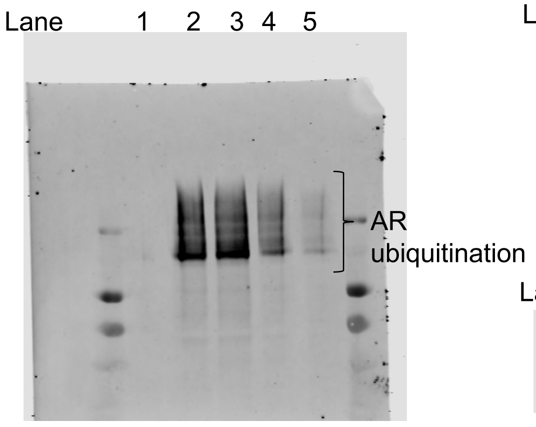

AR ub

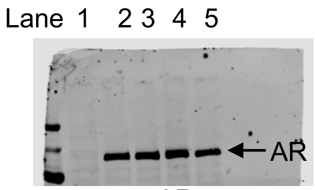

AR

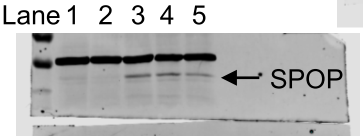

SPOP

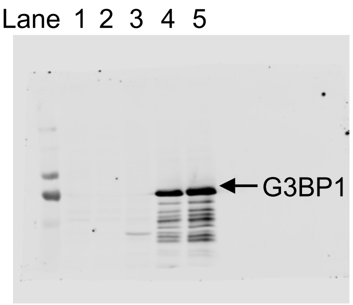

G3BP1

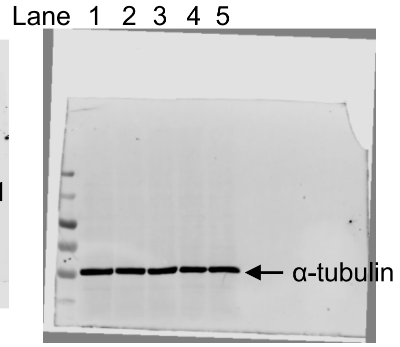

α-tubulin

Lane 1: His ub
Lane 2: His ub+Flag AR
Lane 3: His ub+Flag AR+ HA-SPOP
Lane 4: His ub+Flag AR+ HA-SPOP+ Myc G3BP1 (Low G3BP1 expression)
Lane 5: His ub+Flag AR+ HA-SPOP+ Myc G3BP1 (High G3BP1 expression)

Immunoblot images depicted in Fig. 1c

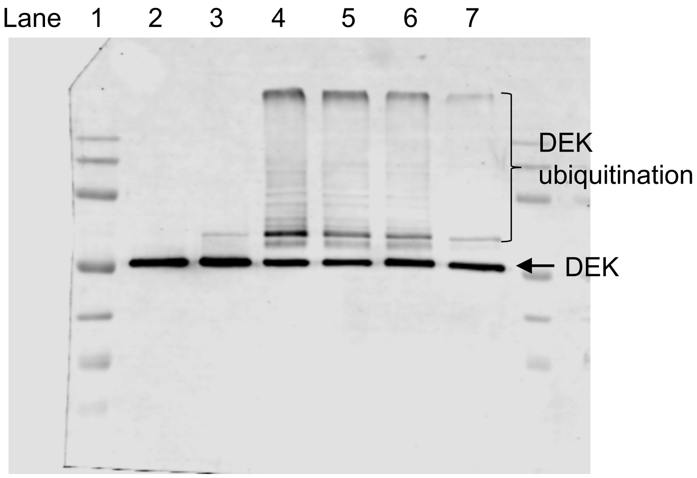

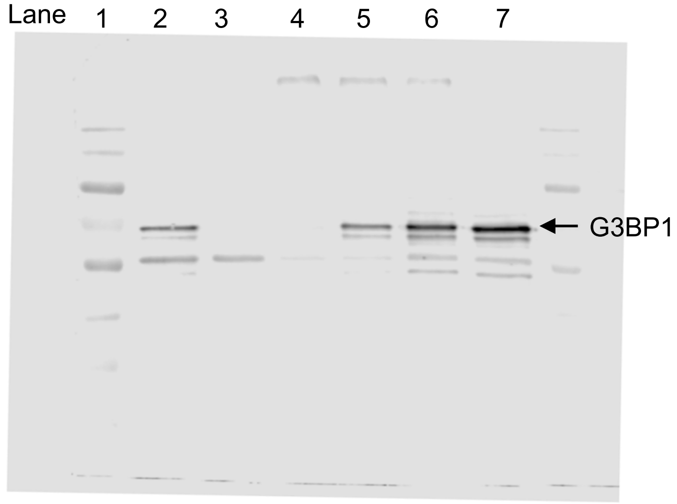

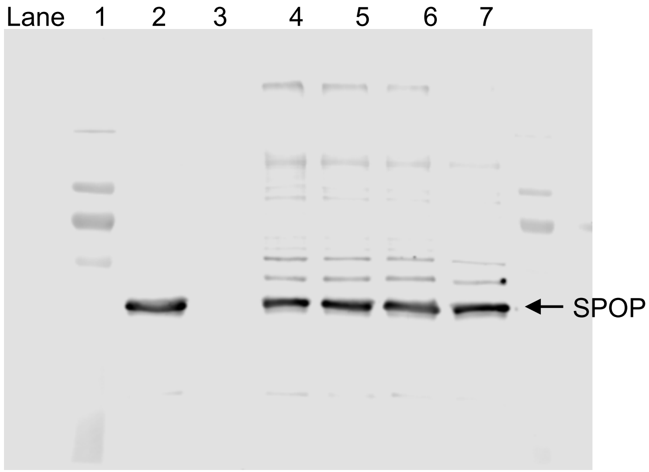

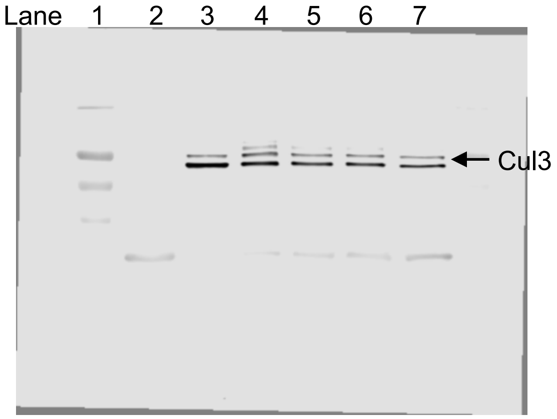

Lane 1: Marker
Lane 2:affinity purified DEK protein
Lane 3:affinity purified DEK and CUL3 proteins
Lane 4: affinity purified DEK and CUL3 and SPOP proteins
Lane 5: affinity purified DEK and CUL3, G3BP1 (0.25 µg) and SPOP proteins
Lane 6: affinity purified DEK and CUL3, G3BP1 (0.5 µg) and SPOP proteins
Lane 7: affinity purified DEK and CUL3, G3BP1 (1 µg) and SPOP proteins.

Immunoblot images depicted in Fig. 1d

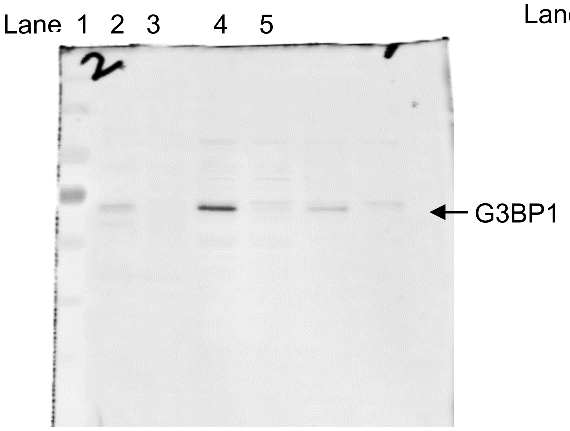

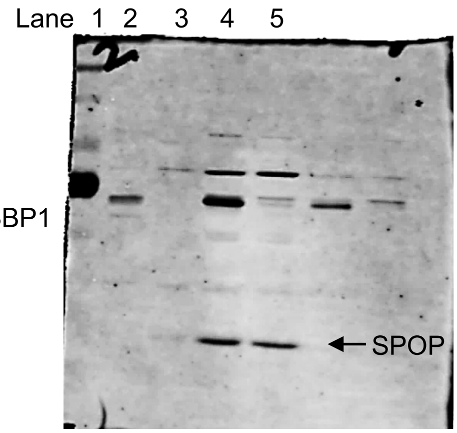

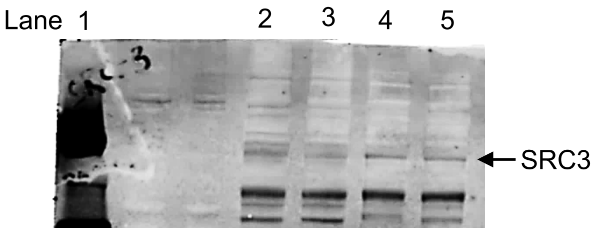

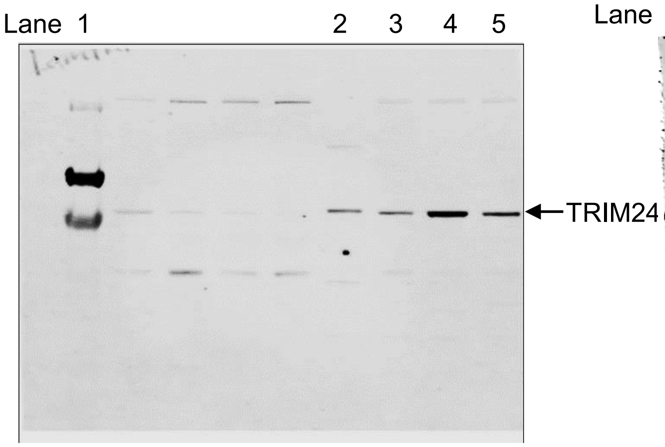

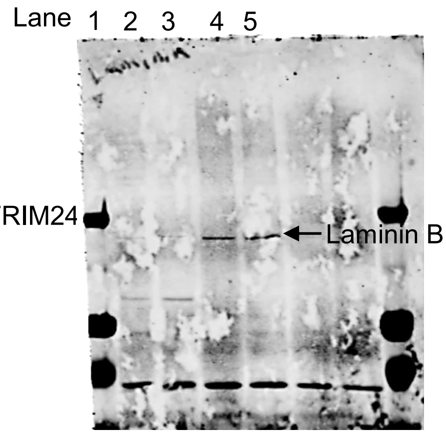

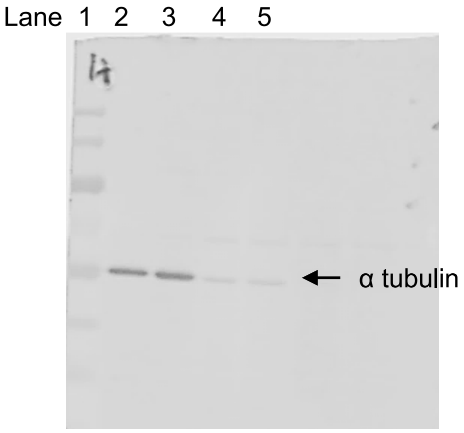

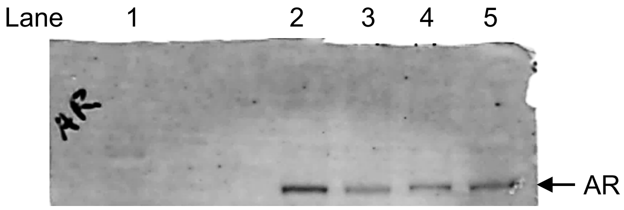

Lane 1: Marker
Lane 2: Cytosolic extract: sgControl
Lane 3: Cytosolic extract: sgG3BP1
Lane 4: Nuclear extract: sgControl
Lane 5: Nuclear extract: sgG3BP1

Immunoblot images depicted in Fig. 1e

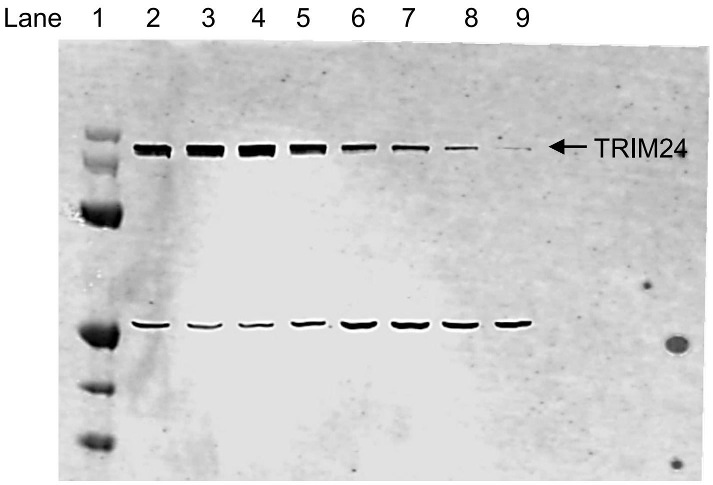

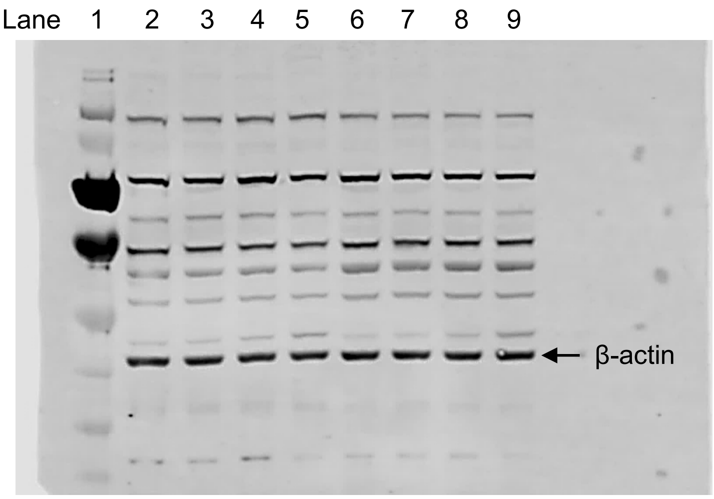

Lane 1: Marker
Lane 2: sgCtrl treated with DMSO
Lane 3: sgCtrl treated with 100μg/ml CHX for 4 h
Lane 4: sgCtrl treated with 100μg/ml CHX for 8 h
Lane 5: sgCtrl treated with 100μg/ml CHX for 12 h
Lane 6: sgG3BP1 treated with DMSO
Lane 7: sgG3BP1 treated with 100μg/ml CHX for 4 h
Lane 8: sgG3BP1 treated with 100μg/ml CHX for 8 h
Lane 9: sgG3BP1 treated with 100μg/ml CHX for 12 h

Immunoblot images depicted in Fig. 2c

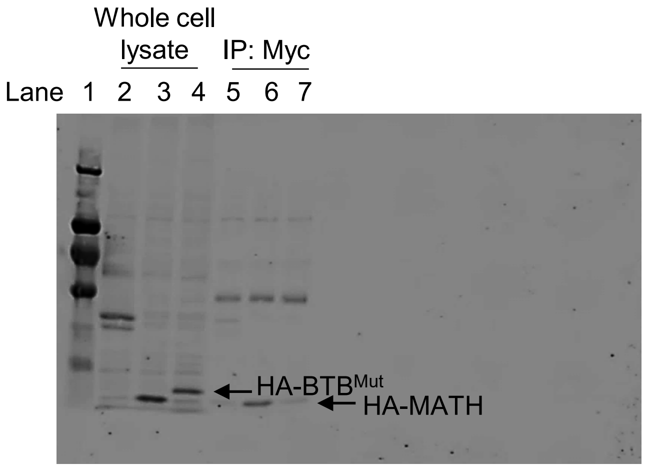

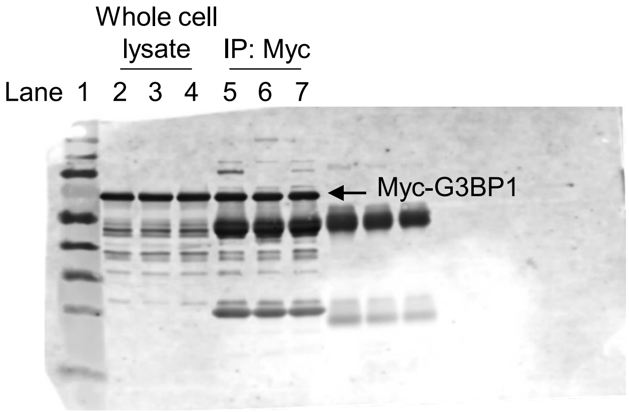

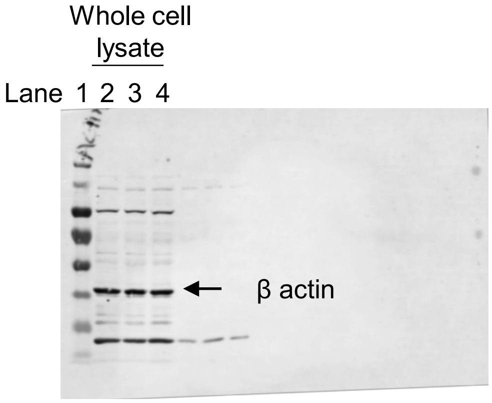

Lane 1: Marker
Whole cell lysate from HEK 293T cells transfected with
Lane 2: Myc-G3BP1 and HA-SPOP
Lane 3: Myc-G3BP1 and HA-MATH
Lane 4: Myc-G3BP1 and HA-BTB^{MUT}
Cell lysate were pulldown with Myc and immunoblotted with HA
Lane 5: HEK 293T cells transfected with Myc-G3BP1 and HA-SPOP
Lane 6: HEK 293T cells transfected with Myc-G3BP1 and HA-MATH
Lane 7: HEK 293T cells transfected with Myc-G3BP1 and HA-BTB^{MUT}

Immunoblot images depicted in Fig. 2d

Lane 1 2 3 4 5 6 7 8 9

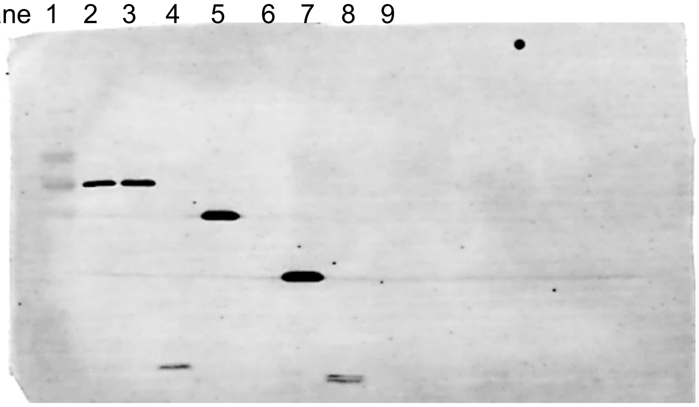

Cell lysate were pulldown with anti-HA and immunoblotted with anti-Flag.

Lane 1 2 3 4 5 6 7 8 9

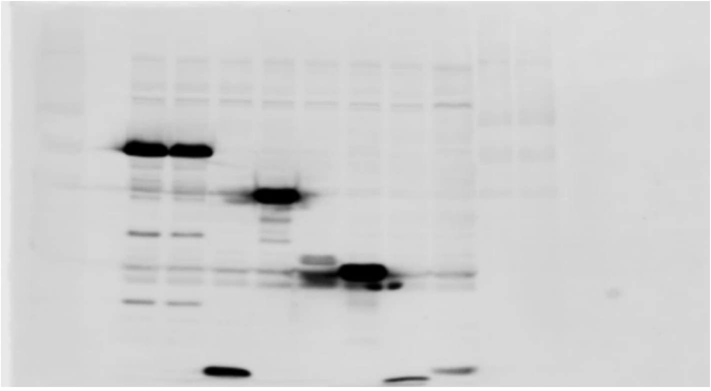

Whole cell lysate was immunoblotted with anti-Flag antibody

Lane 1 2 3 4 5 6 7 8 9

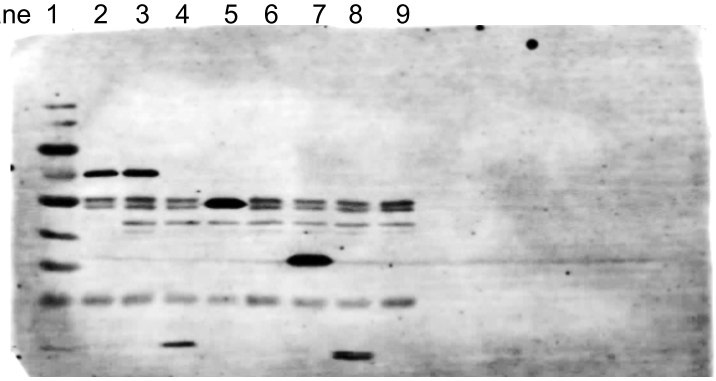

Whole cell lysate was immunoblotted with anti-HA antibody

Lane 1 2 3 4 5 6 7 8 9

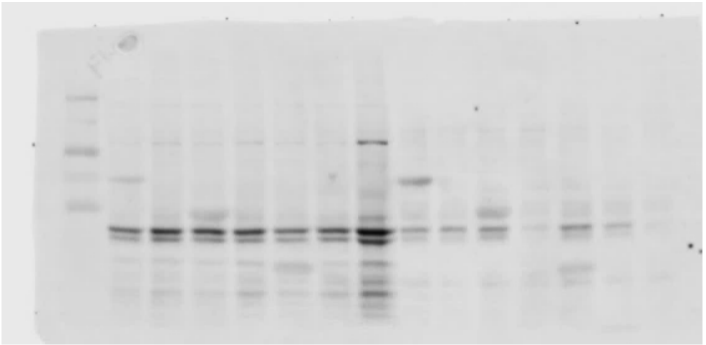

Cell lysate were pulldown with anti-HA and immunoblotted with anti-HA.

Lane 1 2 3 4 5 6 7 8 9

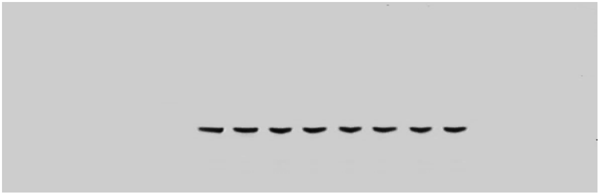

Whole cell lysate was immunoblotted with anti-β-actin antibody

Lane 1: Marker
HEK 293T cells were transfected with
Lane 2: HA-SPOP and Flag-G3BP1
Lane 3: HA-SPOP and Flag-G3BP1^{NTF2}
Lane 4: HA-SPOP and Flag-G3BP1^{M1}
Lane 5: HA-SPOP and Flag-G3BP1^{M2}
Lane 6: HA-SPOP and Flag-G3BP1^{M4}
Lane 7: HA-SPOP and Flag-G3BP1^{C}
Lane 8: HA-SPOP and Flag-G3BP1^{M3}

Immunoblot images depicted in Fig. 2e

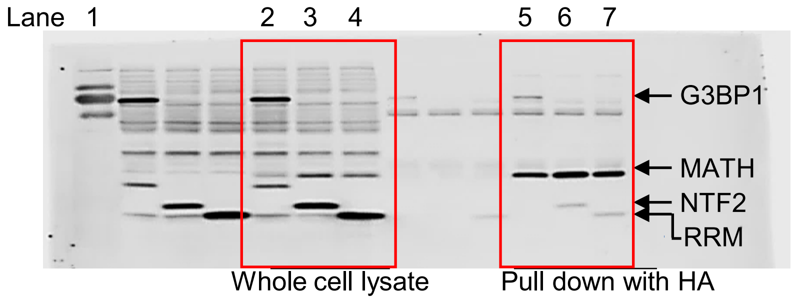

Low exposure

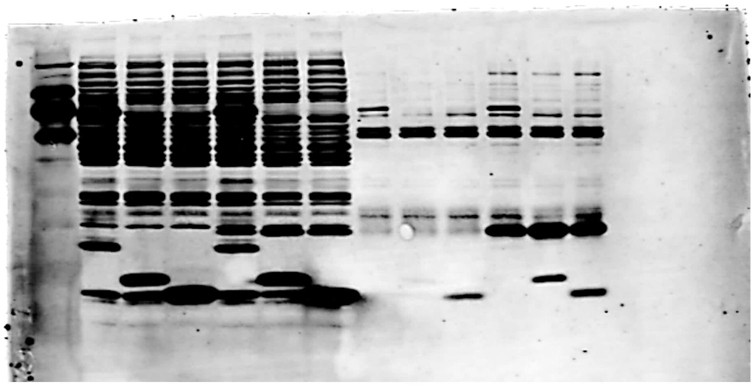

High exposure

Lane 1: Marker
HEK 293T cells were transfected with
Lane 2 and 5: HA-MATH and Flag-G3BP1
Lane 3 and 6: HA-MATH and Flag-G3BP1^{NTF2}
Lane 4 and 7: HA-MATH and Flag-G3BP1^{RRM}

Immunoblot images depicted in Fig. 2g

Whole Cell Lysate

Lane 1 2 3 4

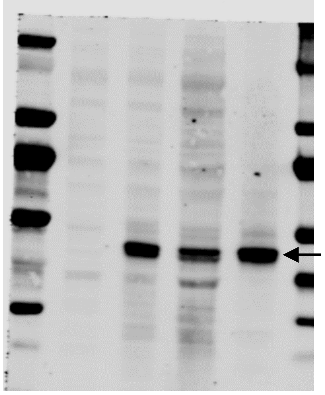

HA-SPOP

HA-SPOP

Whole Cell Lysate

Lane 1 2 3 4

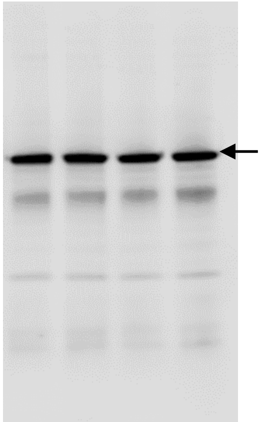

G3BP1

G3BP1

Whole Cell Lysate

Lane 1 2 3 4

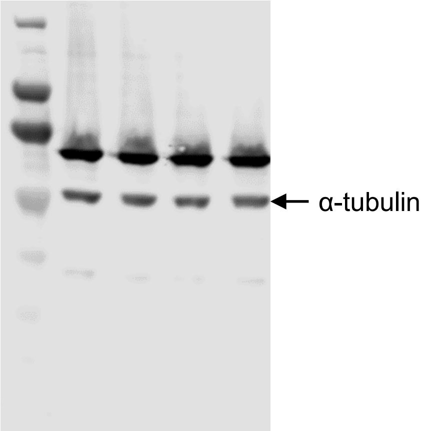

α-tubulin

α-tubulin

IP-G3BP1

Lane 1 2 3 4

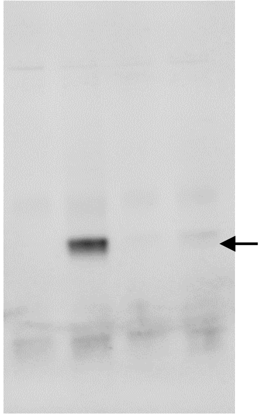

HA-SPOP

HA-SPOP

IP-G3BP1

Lane 1 2 3 4

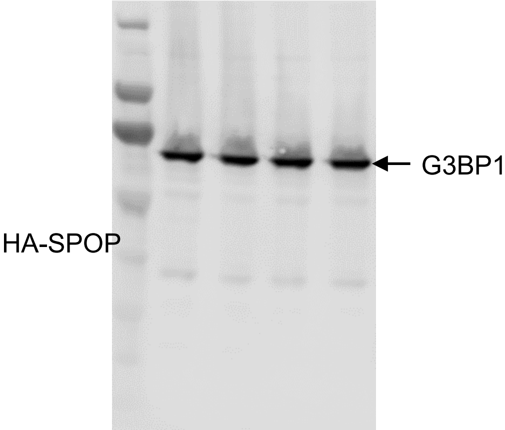

G3BP1

G3BP1

HEK293T cells were transfected with different plasmid and IP-western was performed

Lane 1: Not transfected

Lane 2 : HA-SPOP^{WT}

Lane 3: HA-SPOP^{F133V}

Lane 4: HA-SPOP^{F101C}

Immunoblot images depicted in Fig. 2h

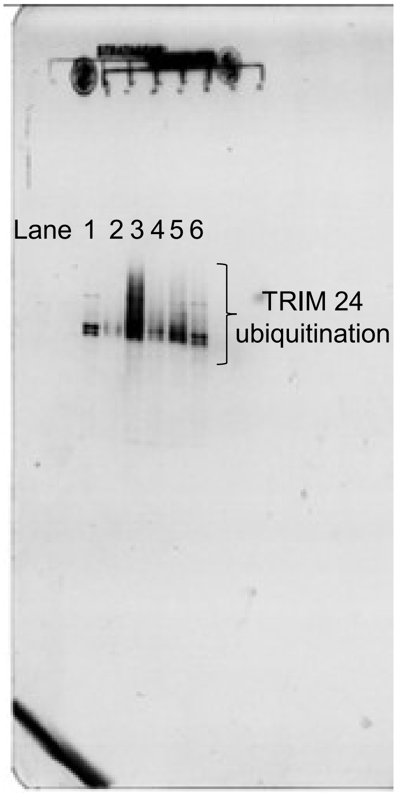

Cell lysate were pulldown and immunoblotted with anti-Flag.

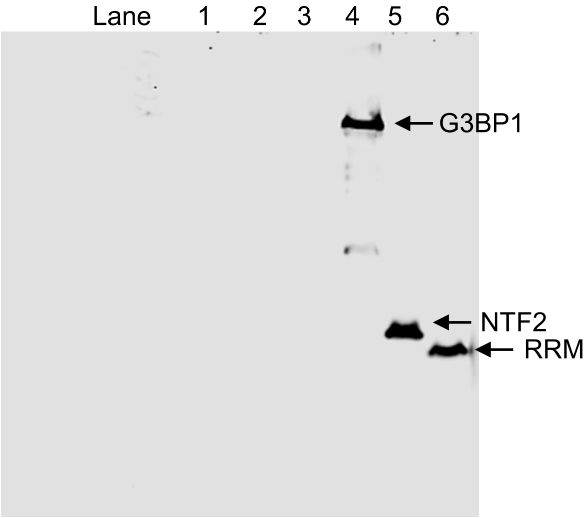

Cell lysate were immunoblotted with anti-Flag.

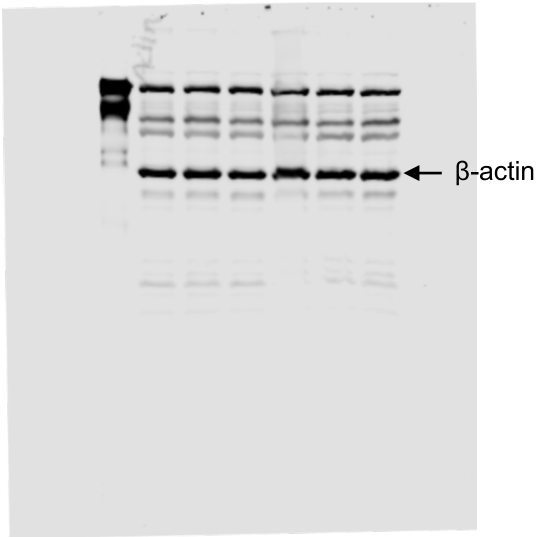

Cell lysate were immunoblotted with anti-β-actin.

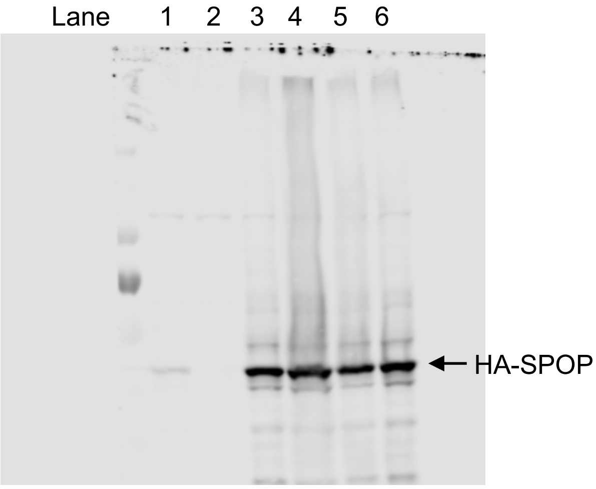

Cell lysate were immunoblotted with anti-HA.

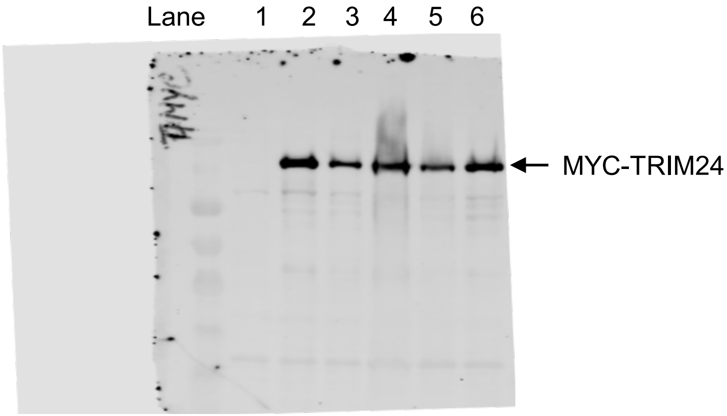

Cell lysate were immunoblotted with anti-MYC.

Immunoblot images depicted in Fig. 5c

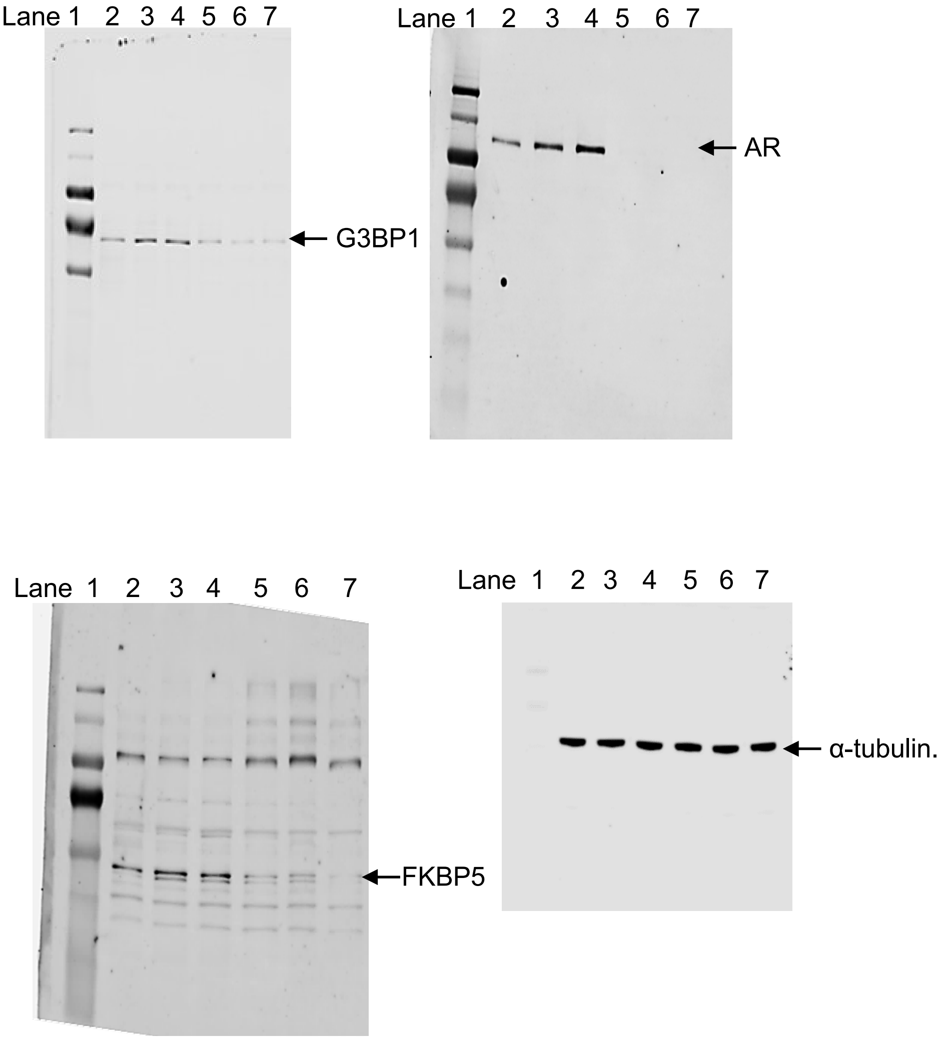

Lane 1: Marker
Lane 2 : control cell treated with DMSO
Lane 3 : control cell treated with 1nM DHT
Lane 4 : control cell treated with 10nM DHT
Lane 5 : AR KO cell treated with DMSO
Lane 6 : AR KO cell treated with 1nM DHT
Lane 7 : AR KO cell treated with 10nM DHT

Immunoblot images depicted in Fig. 5e

Nuclear extract

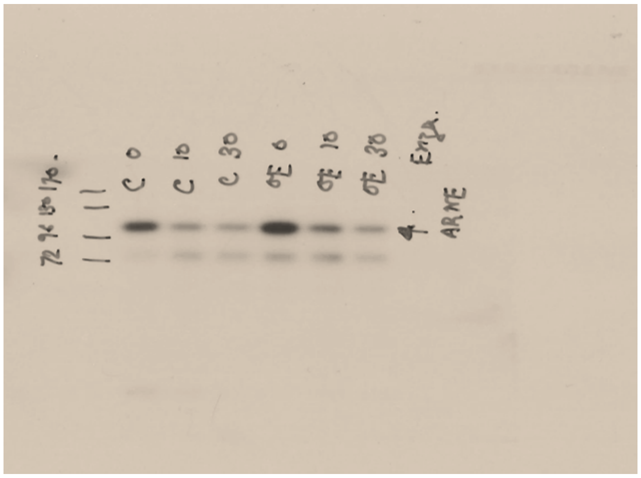

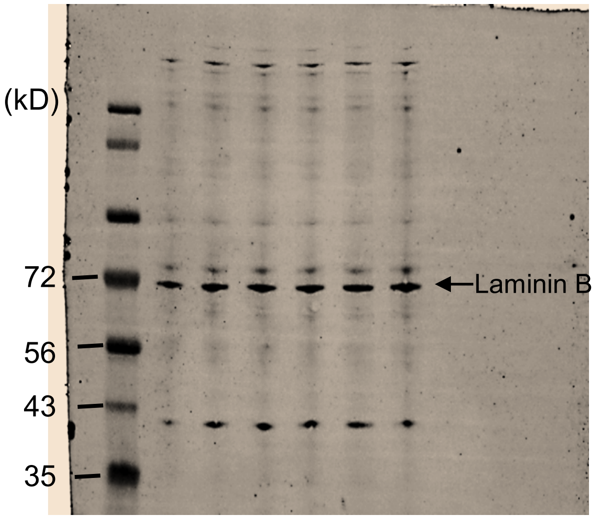

Whole cell lysate

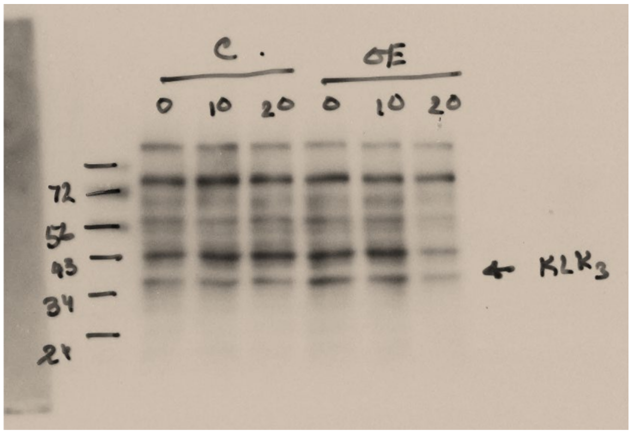

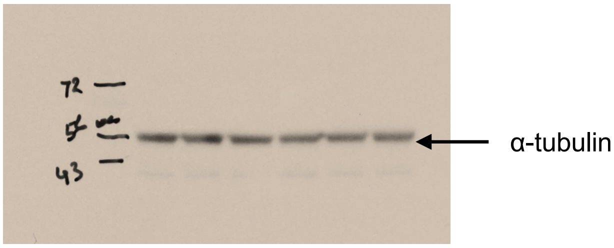

α-tubulin

Immunoblot images depicted in Fig. 7b

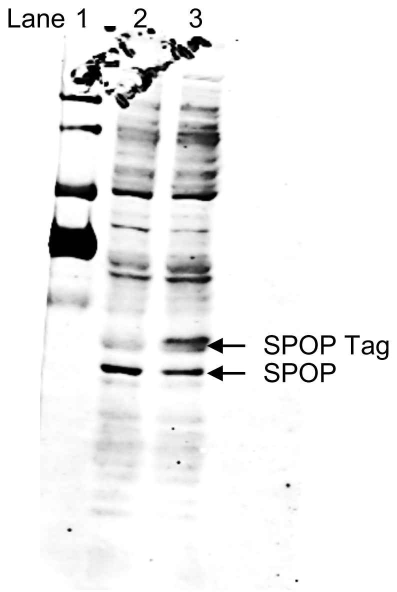

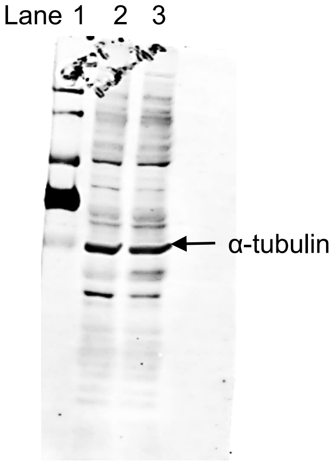

Lane 1: Marker
Lane 2 : control cell lysate
Lane 3 : SPOP^{Mut} cell lase

Immunoblot images depicted in Fig. 7c

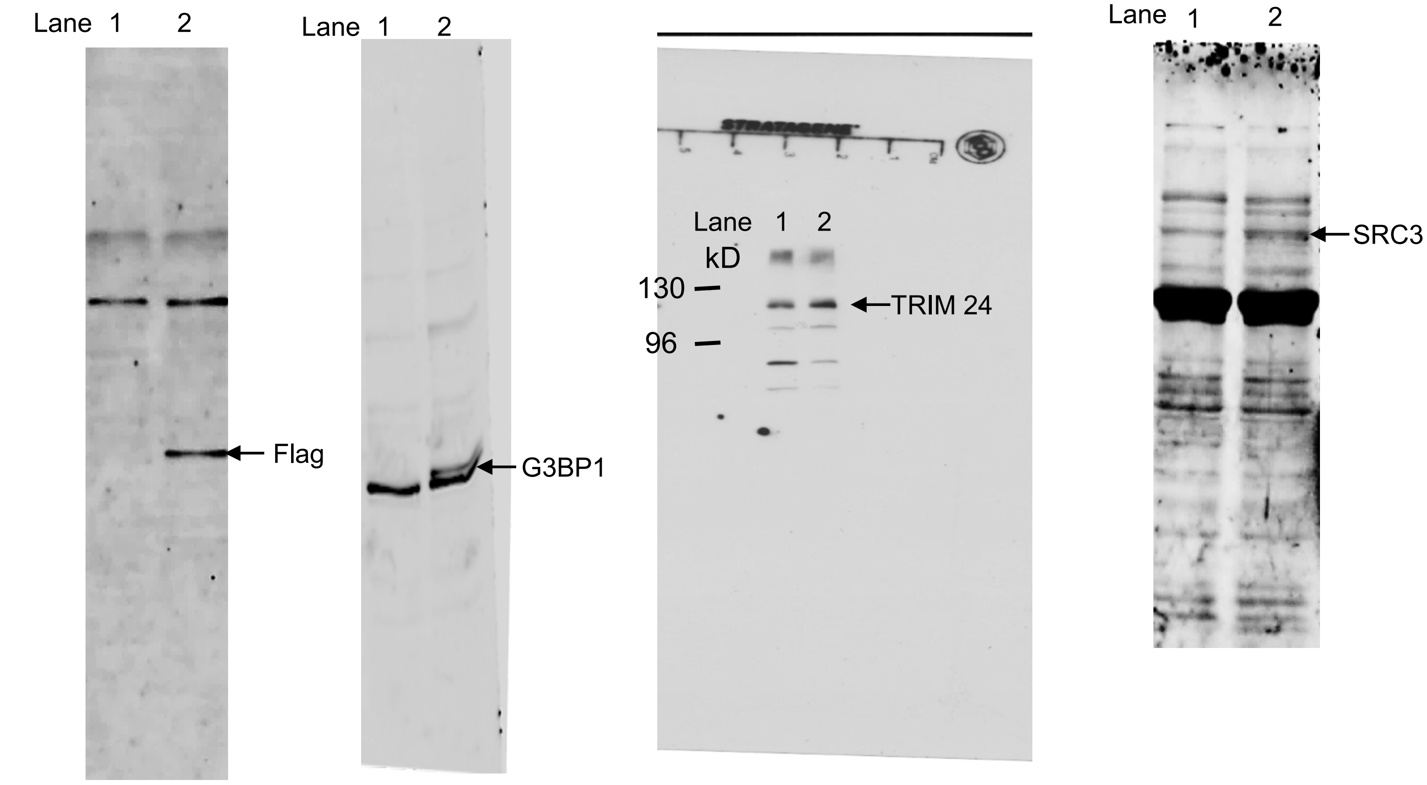

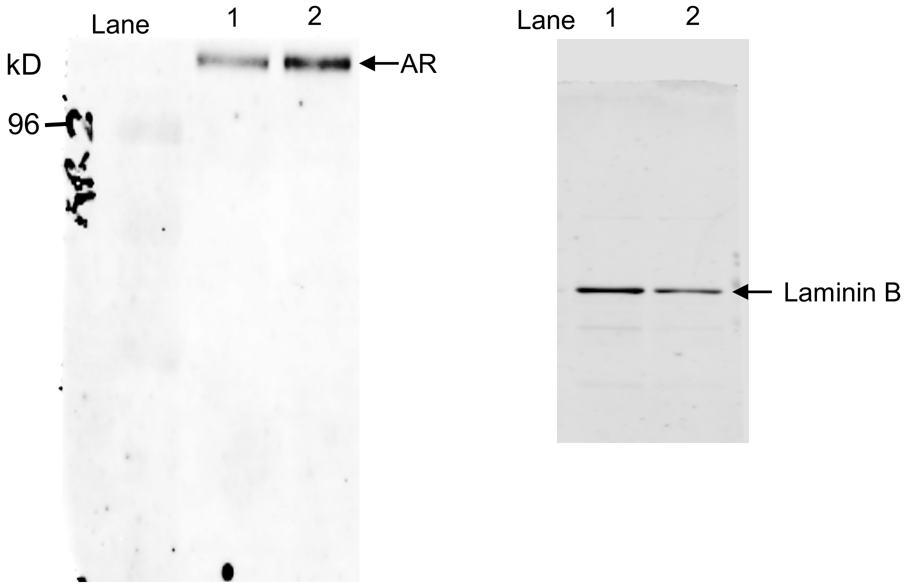

Lane 1: primary mouse prostate epithelial cell lysate (control)
Lane 2 : primary mouse prostate epithelial cell lysate (G3BP1 overexpressed)

Immunoblot images depicted in Fig. 7d

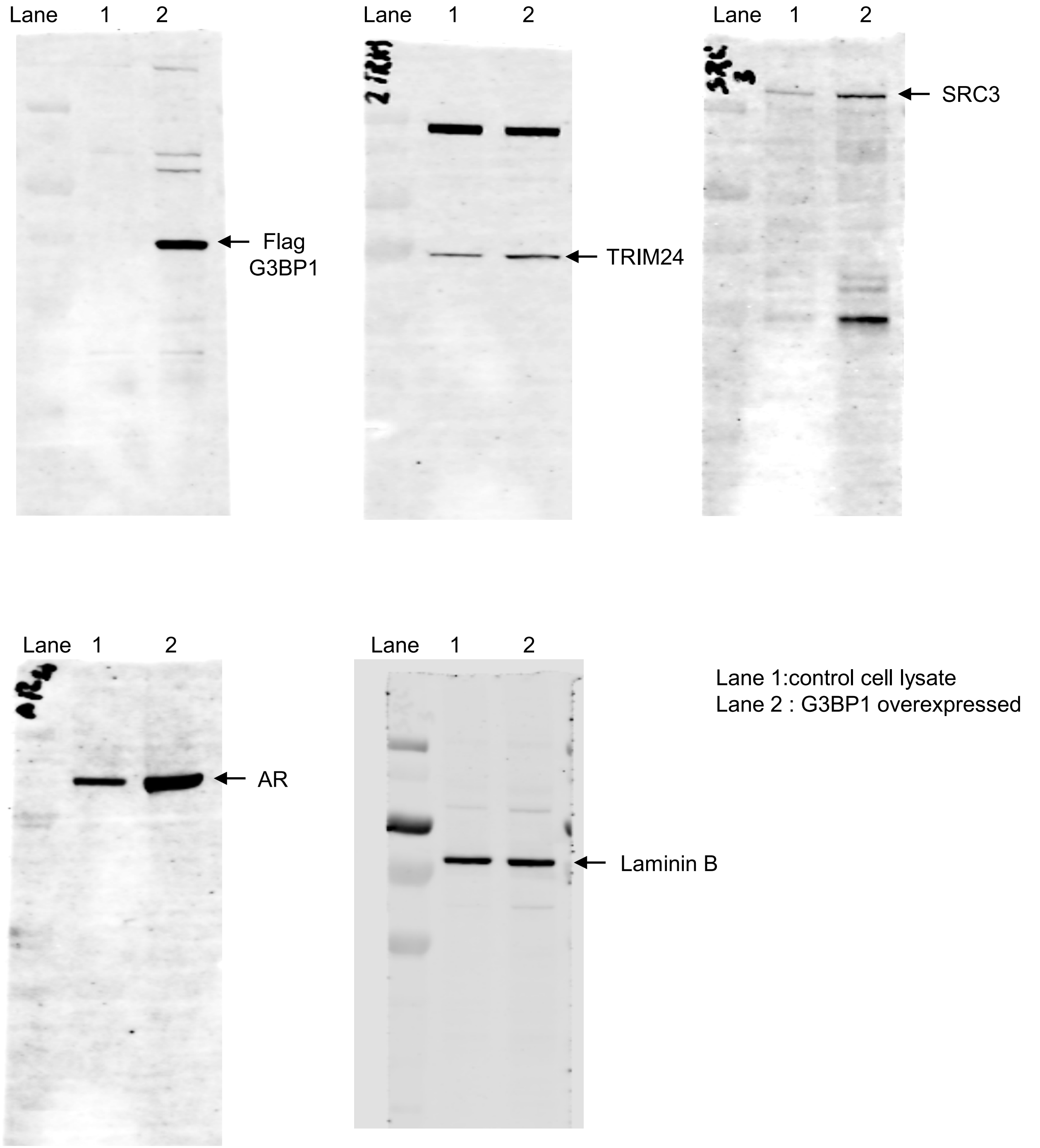

Immunoblot images depicted in Fig. 7f

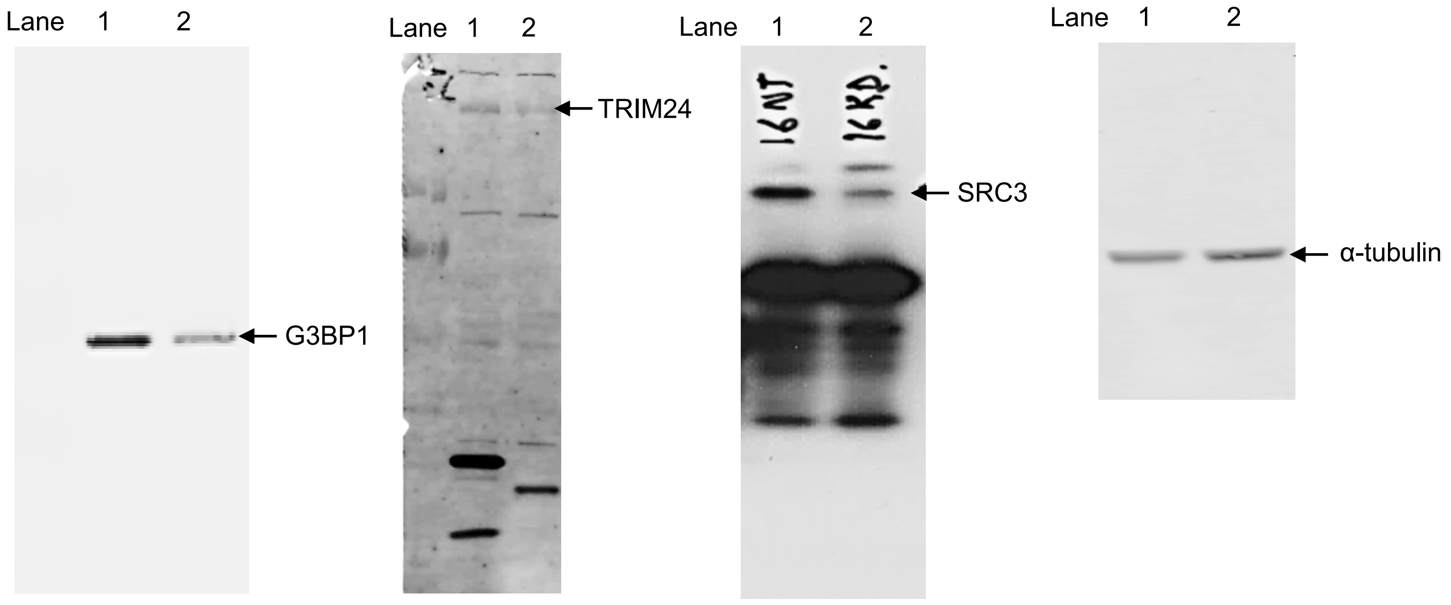

Lane 1:control cell lysate
Lane 2 : G3BP1KO cell lysate

Immunoblot images depicted in Fig. 7h

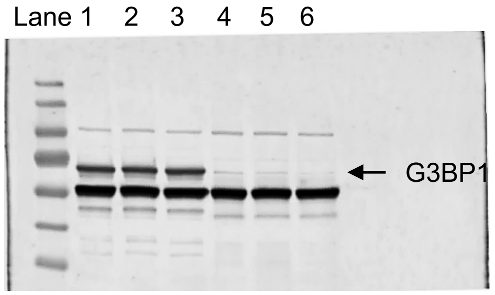

G3BP1

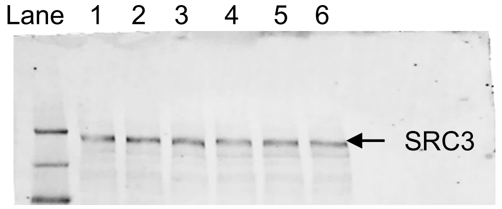

SRC3

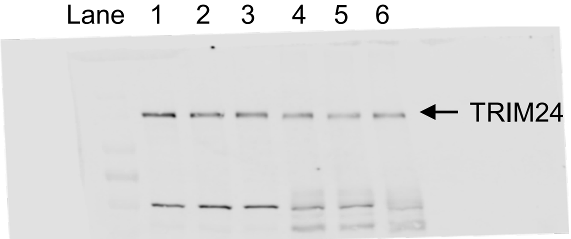

TRIM24

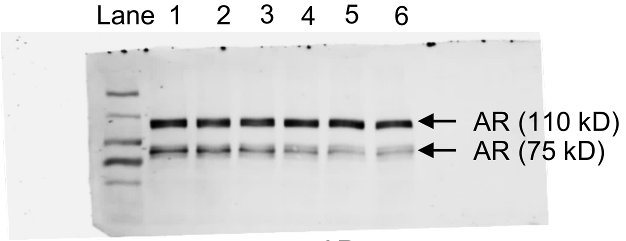

AR

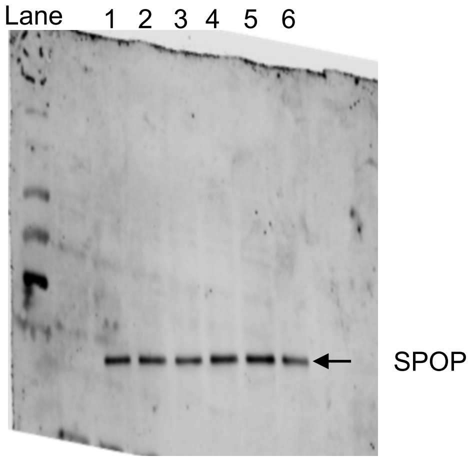

SPOP

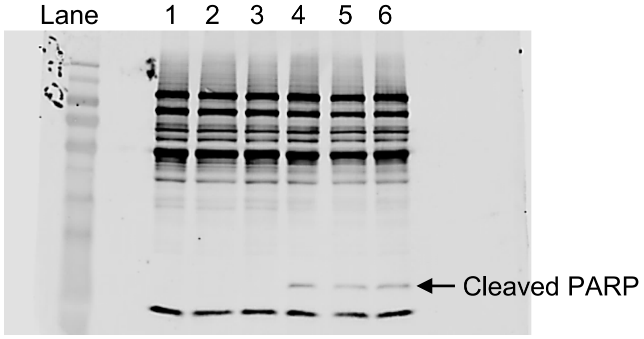

Cleaved PARP

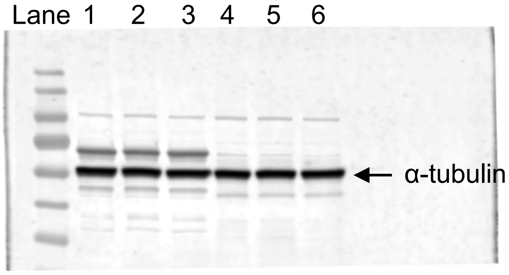

α-tubulin

Xenografted tumor lysate
Lane 1:Tumor 1 control
Lane 2 : Tumor 2 control
Lane 3: Tumor 3 control
Lane 4:G3BP1 KO Tumor 1
Lane 5 : G3BP1 KO Tumor 2
Lane 6: G3BP1 KO Tumor 3
